# Identification of long non-coding RNAs involved in neuronal development and intellectual disability

**DOI:** 10.1038/srep28396

**Published:** 2016-06-20

**Authors:** Eva D’haene, Eva Z. Jacobs, Pieter-Jan Volders, Tim De Meyer, Björn Menten, Sarah Vergult

**Affiliations:** 1 Center for Medical Genetics, Ghent University, Ghent University Hospital, Ghent, Belgium; 2 Bioinformatics Institute Ghent, Ghent University, Ghent, Belgium; 3 Dept. of Mathematical Modelling, Statistics and Bioinformatics, Ghent University, Ghent, Belgium

## Abstract

Recently, exome sequencing led to the identification of causal mutations in 16–31% of patients with intellectual disability (ID), leaving the underlying cause for many patients unidentified. In this context, the noncoding part of the human genome remains largely unexplored. For many long non-coding RNAs (lncRNAs) a crucial role in neurodevelopment and hence the human brain is anticipated. Here we aimed at identifying lncRNAs associated with neuronal development and ID. Therefore, we applied an integrated genomics approach, harnessing several public epigenetic datasets. We found that the presence of neuron-specific H3K4me3 confers the highest specificity for genes involved in neurodevelopment and ID. Based on the presence of this feature and GWAS hits for CNS disorders, we identified 53 candidate lncRNA genes. Extensive expression profiling on human brain samples and other tissues, followed by Gene Set Enrichment Analysis indicates that at least 24 of these lncRNAs are indeed implicated in processes such as synaptic transmission, nervous system development and neurogenesis. The bidirectional or antisense overlapping orientation relative to multiple coding genes involved in neuronal processes supports these results. In conclusion, we identified several lncRNA genes putatively involved in neurodevelopment and CNS disorders, providing a resource for functional studies.

Intellectual disability (ID) affects approximately 1–3% of the general population^1^ and can be caused by any condition that impairs the development and proper functioning of the human brain. Not only is ID a lifelong problem, it has a strong socio-economic impact on both patients and their families.

Both genetic and environmental factors play an important role in human cognition and hitherto, approximately 28% of ID cases can be explained by genetic factors^2^. The diagnostic yield has increased significantly over the years, first through the implementation of genomic microarrays^3^ and more recently by the use of exome sequencing. Recently it was shown that in approximately 16–31% of patients with ID, a causal mutation in a known ID gene can be identified using a trio based exome sequencing approach^4^,^5^. In an additional ~20% of patients, a *de novo* mutation was identified in a new candidate ID gene^4^,^5^,^6^. Notwithstanding this progress, for the majority of patients the underlying cause of ID remains unexplained, thus warranting further research. Although whole genome sequencing has been used to identify pathogenic mutations^7^, the subsequent analysis mainly targeted the coding part of the genome as our understanding of non-coding variation is still limited. As such, the non-coding part of the human genome remains largely unexplored. Recent evidence shows that a specific class of non-coding RNAs, so-called long non-coding RNAs (lncRNAs; defined as transcripts longer than 200 bp in length without protein coding potential) play important and diverse functions in gene regulation and protein interactions^8^,^9^,^10^,^11^,^12^. Of particular importance, many of these lncRNAs emerged recently during vertebrate and primate evolution and are anticipated to be of crucial importance in the most highly evolved and complex human organ, the brain^13^,^14^,^15^. Non-coding RNAs have indeed been linked to brain complexity and development with a possible role in brain cellular diversity, amongst others^16^,^17^,^18^,^19^,^20^.

Moreover, a substantial percentage of disease association signals of genome wide association studies (GWAS) performed for many central nervous system (CNS) disorders, map to such expressed non-coding regions in the human genome^21^. From several studies, it has become apparent that these CNS disorders (e.g. schizophrenia and bipolar disorder) have a fundamental overlap in biological pathways with ID^22^,^23^,^24^. These pathways affect synapse formation and maintenance, neurotransmission, as well as chromatin regulation and organization. The dysfunction of specific neuronal networks underlying the particular symptoms of each clinical condition most likely depends on additional genetic, epigenetic, and environmental factors that remain to be characterized.

Previous studies have used microarray or RNA-seq expression profiling to identify lncRNAs that are upregulated during neuronal development^25^,^26^ or differentially expressed in tissue samples of patients with autism spectrum disorders (ASD) or major depressive disorder (MDD)^27^,^28^,^29^. Additionally, in silico approaches have also been used to find noncoding antisense transcripts associated with ASD-genes^30^. In this study, we aimed to identify candidate lncRNAs associated with neuronal development and ID through an integrated genomics approach. By combining our in-house lncRNA database LNCipedia^31^ with publically available neuronal functional genomics data (H3K4me3 histon mark, REST binding and DNaseI hypersensitivity) we selected strong candidate genes for ID and neurodevelopmental disorders. These data respectively mark active promoters, neuronal genes silenced in nonneuronal tissues and transcriptionally active regions.

To test our hypothesis that these (epi) genetic features are relevant for the identification of candidate lncRNAs, we applied a validation strategy in which we selected RefSeq protein-coding genes and lncRNA transcripts characterized by these features. Subsequently, we performed an enrichment analysis of GWAS hits for CNS disorders and, for the former gene set, known and candidate ID genes. Identification of the most relevant feature resulted in a list of candidate lncRNAs. This analysis was further complemented by extensive expression profiling of all protein-coding genes and ca. 23,000 lncRNA transcripts in 15 human tissues, among which 8 brain samples. Next to providing insights into overall expression patterns of lncRNAs in human brain regions, this allowed us to construct coexpression profiles for the identified lncRNAs. Through subsequent Gene Set Enrichment Analysis (GSEA) and exploration of the genomic neighbourhood we assigned putative biological functions to the selected lncRNAs.

## Results

### Enrichment analysis shows that neuron-specific H3K4 trimethylation confers the highest specificity for genes involved in ID and neurodevelopment

**Figure 1:**
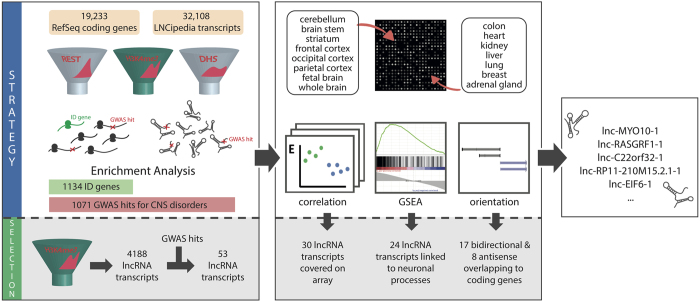
Overview of the strategy and selection process. Overview of the strategy followed in this paper to identify an appropriate filtering approach and select lncRNAs with a putative role in ID and neuronal development. Phase I: In order to identify an appropriate filtering strategy, an enrichment analysis of ID genes and GWAS hits was performed on sets of RefSeq protein-coding genes and LNCipedia lncRNA transcripts passing the following filters: set 1 = DNAse I hypersensitivity, set 2 = neuron-specific H3K4me3, set 3 = REST binding. Phase II: Microarray expression profiling of all protein-coding genes and 22,980 lncRNAs in 8 human brain samples and 7 other human tissues allowed us to calculate mRNA:lncRNA correlation for selected lncRNA transcripts. Subsequent Gene Set Enrichment Analysis and examination of the orientation relative to and function of cis coding genes provided insight into putative functions of the selected lncRNAs.

**Figure 2:**
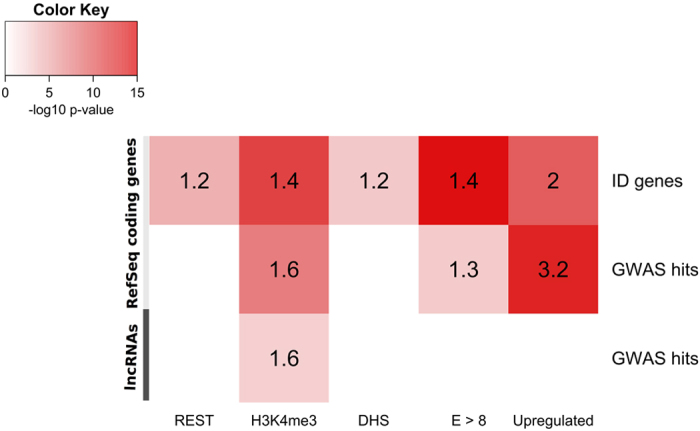
Neuron-specific H3K4me3 confers the highest specificity for ID genes and genes harbouring GWAS hits. Enrichment of ID genes and GWAS hits associated with CNS disorders in resulting sets of protein-coding genes and lncRNAs after applying REST binding sites, neuron-specific H3K4me3 marks, DNAseI hypersensitivity, upregulation in neuronal tissues & expression in at least one neuronal tissues (normalized log2 transformed expression value (E) >8) as filters. P-values were determined according to Fisher’s exact test. Odds ratios are indicated within the boxes.

Since the function of most lncRNAs remains elusive, we tried to define a strategy to identify lncRNAs with a putative role in neuronal development and ID by combining several (epi) genomic and transcriptomic datasets (Overview in Fig. 1). Specifically, we identified protein-coding genes and lncRNAs that present with one of the following features in the promoter region (see Materials and Methods). (1) As a first mark, enriched H3K4me3 peaks in neuronal samples compared to nonneuronal controls (short: neuron-specific H3K4me3), indicative for an active promoter region, were included. (2) Secondly, REST (RE1 Silencing Transcription factor) binding was included as one of the criteria, since REST is involved in silencing neuronal genes in non-neuronal tissues^32^. (3) Finally, we included DNAse 1 hypersensitivity in embryonic and neural cell lines as a mark for active transcription. The performance of these features to delineate genes involved in neuronal development and ID was assessed by selecting RefSeq protein-coding genes and LNCipedia lncRNA transcripts featured by these marks, followed by enrichment analysis for known and candidate ID genes in the former gene set (protein-coding genes), and for the presence of GWAS hits associated with CNS disorders in both resulting gene sets (protein-coding genes and lncRNAs) (Fig. 2).

Of the RefSeq coding genes (19,233 genes; 38,654 transcripts; UCSC February 2015) 12,696 presented with a REST binding motif and 7,003 with a neuron-specific H3K4me3 mark in the promoter region (Supplemental Table S3). 15,144 RefSeq genes showed DNAse 1 hypersensitivity in the promoter region (Supplemental Table S3). When performing the same analysis for lncRNAs (LNCipedia 2.1; 32,108 transcripts), 11,348 transcripts present with a REST binding site in their putative promoter (Supplemental Table S4). A neuron specific H3K4me3 mark is present in the promoter region of 4,188 lncRNA transcripts and DNAse 1 hypersensitivity was observed in the promoter region of 17,023 transcripts (Supplemental Table S4). Although a significant enrichment of ID genes was noted for all coding genes presenting with either a neuron-specific H3K4me3 modification (p-value = 6.252^*^10^−12^), REST binding sites (p-value = 2.701–10^−5^) or DNAse 1 hypersensitive regions (p-value = 5.646*10^−4^), the largest enrichment was observed for genes overlapping with neuron-specific H3K4me3 (Fig. 2 and Supplemental Table S3). When assessing genes containing selected GWAS hits, the H3K4me3 filter was the only one to result in a significant enrichment (p-value = 2.794*10^−8^) (Fig. 2 and Supplemental Table S3). Enrichment for both ID genes and GWAS hits did not improve significantly when combining the H3K4me3 mark with the two other features (Supplemental Table S3).

Taken together, these observations suggest that the neuron-specific H3K4me3 mark yields the highest specificity for genes involved in ID and neurodevelopment. This was also confirmed for the lncRNAs, since enrichment of lncRNAs harbouring a SNP associated with CNS disorders was only observed within the group of transcripts presenting with a neuron-specific H3K4me3 mark (p-value = 1.957*10^−3^) (Fig. 2 and Supplemental Table S4).

### The genomic neighbourhood of lncRNAs with a neuron-specific H3K4me3 mark

As many lncRNAs are known to perform their regulatory function in *cis*^33^, we subsequently examined the genomic neighbourhood of the resulting set of 4,188 lncRNAs with neuron-specifc H3K4me3 marks. Among these, 3,222 overlap with, or are transcribed within 5 kb from a protein-coding gene. Subsequently, we performed enrichment analysis of GO terms for these *cis* coding genes (geneontology.org). GO terms involved in positive regulation of biological processes and nervous system development are clearly represented (Supplemental Fig. S2, Supplemental Table S5). Additionally, among the 4,188 lncRNAs characterized by neuron-specific H3K4me3, 53 harbour a GWAS hit for CNS disorders within their sequence. As the presence of such a SNP directly implicates these loci in neuropathogenesis, we subsequently focussed our analyses on this set of 53 lncRNAs (list in Supplemental Table S6).

When zooming in on the genomic neighbourhood of these 53 lncRNAs, 44 overlap with, or are transcribed within 5 kb from, a protein-coding gene. Many of these are either transcribed bidirectionally from the same promoter region as the coding gene (28 transcripts) or in an antisense manner covering a large part of the coding gene body (12 transcripts) (Supplemental Table S7). On the other hand, three selected transcript clusters and one single transcript are situated more than 50 kb away from the nearest protein-coding gene (lnc-C22orf32-1, lnc-AC073043.2.1-1, lnc-USP25-2 & lnc-DPYD-4:1) and are transcribed from alone-standing promoter regions characterized by their own H3K4me3 marks, DHS regions, transcription factor clusters and CpG islands.

### Expression profiling of lncRNAs in neuronal and non-neuronal tissues

Extensive expression profiling of all protein-coding genes and ca. 23,000 lncRNA transcripts was performed for 15 different human tissues: heart, adrenal gland, breast, kidney, lung, colon and liver were used as non-neuronal controls, while 8 different brain samples were evaluated as neuronal tissues. Of the 4,188 lncRNA transcripts with a H3K4me3 mark, 2,636 were covered on the custom designed array (30/53 transcripts overlapping with both a H3K4me3 mark and a GWAS hit) (Supplemental Table S8). No unique probes could be designed for the other transcripts. First we assessed whether identifying upregulated genes in neuronal samples would confer sufficient specificity to delineate the lncRNAs involved in neurodevelopment and ID. The evaluation strategy we applied was the same as described above, i.e. assessing enrichment of ID genes in upregulated protein-coding genes and determining enrichment of genes harbouring a GWAS SNP for both upregulated protein-coding genes and lncRNAs.

RankProduct analysis of the protein-coding genes between neuronal and non-neuronal tissues revealed an upregulation of 1,290 and a downregulation of 790 genes (FDR 0.01) (Supplemental Table S8). Upregulated genes showed significant enrichment for both ID genes (124/1290, p = 3*10^−10^) and genes harbouring GWAS hits (65/1290, p = 7.5*10^−14^) (Fig. 2 and Supplemental Table S8). When considering lncRNAs, 731 and 237 transcripts were respectively up- and downregulated in neuronal tissues (FDR 0.01). However, only 4 upregulated lncRNAs harbour a GWAS hit (lnc-RP11-210M15.2.1-1:3, lnc-AC073043.2.1-1:1, lnc-APOB-8:1 & lnc-MYO10-1:1). Hence, when employing upregulation as a filter for the lncRNAs, no significant enrichment for transcripts comprising GWAS hits could be observed (4/731) (Fig. 2 and Supplemental Table S8).

**Figure 3:**
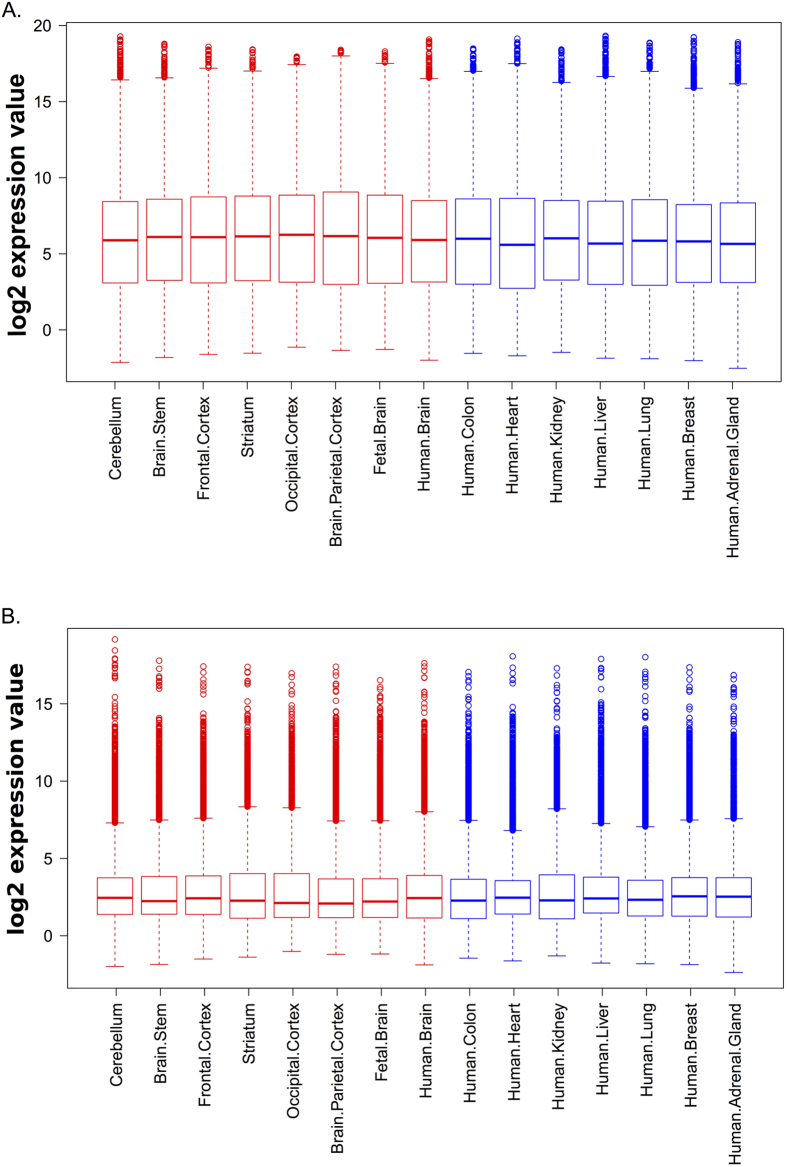
lncRNAs display lower overall expression compared to protein coding genes. mRNA vs lncRNA expression levels. Boxplots of normalized log2 expression values in the 15 tested tissues. (**A**) Coding genes (**B**) lncRNAs. The data for neural and nonneuronal tissues is respectively shown in red and blue.

Only 3 out of the 30 lncRNA transcripts presenting with a H3K4me3 mark and a GWAS associated SNP that were covered on the array, were significantly differentially expressed between brain regions and nonneuronal tissues. This might be explained by the fact that genes involved in neuronal development might have a different expression in fetal and adult whole brain as well as in different adult brain tissues. Moreover, it is well established that lncRNAs exhibit an overall lower expression than protein-coding genes (Fig. 3). This suggests that it might be more relevant to consider the expression profile of lncRNAs within single neuronal tissues instead of assessing differential expression between all brain tissues and nonbrain tissues. To account for this, we implemented a less stringent strategy consisting of a normalized log2 expression value >8 in at least one brain sample, which corresponds approximately to the upper quartile of protein-coding expression values (Fig. 3A). For protein-coding genes, this again resulted in an enrichment of ID genes (839/11472, p-value = 6.9*10^−15^) and genes harbouring GWAS hits (243/11472, p-value = 9*10^−4^) (Fig. 2 and Supplemental Table S8). However, again no significant enrichment for lncRNAs containing GWAS hits was observed for the remaining lncRNA transcripts (5/1350) (Fig. 2 and Supplemental Table S8).

### Predicting putative functions for lncRNAs with a neuron-specific H3K4me3 mark and harbouring a SNP associated with CNS disorders

To identify putative functions for the 30 selected lncRNAs with expression data, we employed a guilt-by-association approach based on correlation in expression profiles with all protein-coding genes.

**Figure 4:**
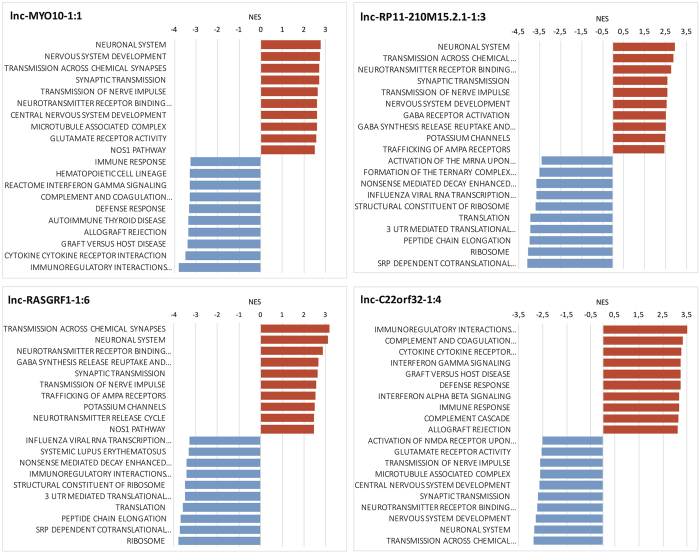
Gene set enrichment analysis links selected genes to neuronal processes. Normalized enrichment scores (NES) of the top 10 positively and negatively enriched genes sets (abs(NES) > 2 & FDR < 0.25) among protein-coding genes highly (anti-)correlated to lnc-MYO10-1:1, lnc-RASGRF1-1:6, lnc-RP11-210M15.2.1-1:3 & lnc-C22orf32-1:4. A positive NES reflects enrichment of the gene set at the top of the ranked list, i.e. genes highly, positively correlated to the lncRNA in question. Gene sets with a negative NES are overrepresented at the bottom of the gene list, i.e. among negatively correlated protein-coding genes. These results can also be found in Supplemental Table S10, which, next to the NES, also displays non-abbreviated gene set names, gene set sizes, enrichment scores and FDR q-values.

For each lncRNA, protein-coding genes were ranked according to their pairwise Spearman’s correlation coefficient. Subsequently, these ranked lists were used to perform a preranked Gene Set Enrichment Analysis (GSEA). For 19 out of the 30 selected lncRNAs, gene sets highly enriched (abs(NES)>2, FDR < 0.25) among the top positively correlated genes were linked to synaptic transmission, nervous system development or neurogenesis (example for the lncRNAs showing the strongest enrichment (lnc-MYO10-1:1, lnc-RASGRF1-1:6 & lnc-RP11-210M15.2.1-1:3) in Fig. 4, all GSEA results in Supplemental Fig. S1). Five lncRNAs negatively correlate to genes involved in these neuronal processes, suggesting that these lncRNAs may be involved in suppressive regulation of such genes (example for lnc-C22orf32-1:4 in Fig. 4). For an additional three lncRNAs, highly correlated genes were involved in mitochondrial energy metabolism (Supplemental Fig. S1).

**Figure 5:**
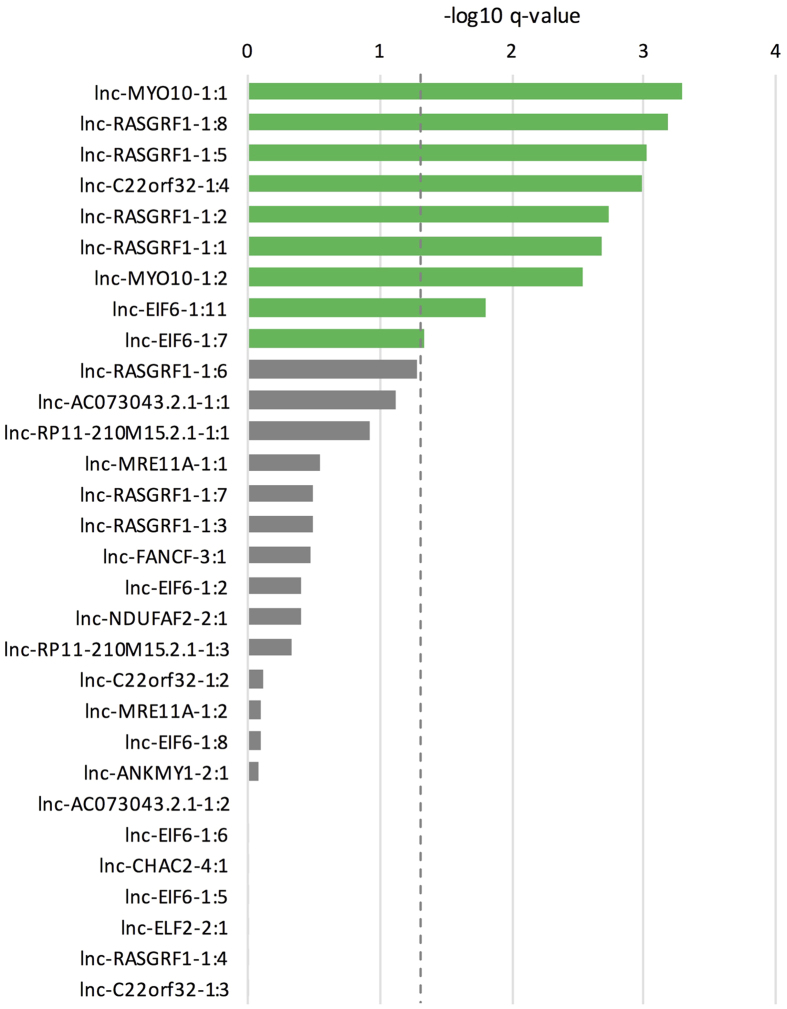
Highly correlated coding genes show enrichment for ID genes. Enrichment of ID genes among top correlated (abs(correlation) >0.7) protein-coding genes for each of the selected lncRNAs. The dotted line represents a q-value cut-off of 0.05.

For 9 of the lncRNAs (lnc-MYO10-1 transcripts, lnc-RASGRF1-1 transcripts, lnc-C22orf32-1:4 & lnc-EIF6-1 transcripts), the top ranked correlated protein-coding genes (absolute correlation coefficients higher than 0.7) were significantly enriched for (candidate) ID genes (Fig. 5 and Supplemental Table S9).

Among these 30 selected lncRNAs, 25 transcripts overlap with or are transcribed within 5 kb from the nearest protein-coding gene (Table 1). Ten out of the seventeen transcripts exhibiting bidirectional transcription show a significant, positive correlation (p < 0.05) in expression to their divergently transcribed, coding neighbour. Four out of the eight transcripts with an antisense overlapping transcription were significantly correlated; three in a negative fashion, one positively correlated. These observations suggest that bidirectionally transcribed and antisense overlapping lncRNAs are primarily involved in, respectively, positive and negative regulation of *cis*-genes.

## Discussion

Although literature states that lncRNAs might play an important role in neuronal development and/or intellectual disability, until now, only a handful of these lncRNAs have been identified and functionally validated^26^,^34^,^35^,^36^,^37^. With the advent of next-generation sequencing and consortia such as ENCODE, large (epi)genetic data sets have become available. Here we integrated several publically available datasets and generated extensive expression data to get a holistic view of the potential of lncRNAs in neuronal development and CNS disorders.

To develop a strategy that permits the identification of genes involved in neuronal development and ID, we first applied multiple epigenomic features for all RefSeq protein-coding genes. This resulted in the most significant enrichment of ID genes and genes associated with CNS disorders when applying neuron-specific H3K4me3 as a feature. A large proportion of the known and candidate ID genes (581/1134) fulfilled this criterion, including several of our recently reported candidate ID genes such as *MYT1L, DEAF1, CACNA2D1* and *POU3F3*^38^,^39^,^40^,^41^,^42^. For those that do not present with neuron-specific trimethylation of H3K4, an explanation can be found in the function of these genes, as several of them (e.g. *TGFBR1 & TWIST1)* give rise to syndromal forms of ID, indicating these genes also play important roles outside of the CNS.

Using the neuron-specific H3K4me3 mark to identify lncRNAs with putative involvement in neurodevelopment, resulted in a set of 4,188 lncRNA transcripts. This set includes almost all lncRNAs that have been demonstrated in literature to play a role in neuronal development, such as *MIAT, TUG1, DGCR5, MEG3,* and *TUNA*^26^,^37^,^43^,^44^,^45^,^46^. One lncRNA with validated function during neurogenesis that is notably absent in our candidate list is *RMST*^35^. Although brain specific expression, REST binding and DNAse 1 hypersensitivity were noted for *RMST*, no neuron specific H3K4me3 was observed. However, RMST plays an important role in neuronal differentiation through association with SOX2, which has a neuron specific H3K4me3 mark. Also the lncRNA PNKY^47^, although presenting with a neuron specific H3K4me3 mark, was not selected through our strategy as this transcript was not included in LNCipedia v2.1.

Although the expression data for neuronal and nonneuronal tissues were highly informative for the selection of protein-coding genes (upregulated genes showed very strong enrichment for ID genes and GWAS hit loci), enrichment analysis suggested them to be poor filters for lncRNAs. This might be explained by their overall lower and more region-specific expression profile, which renders differential expression between groupings of multiple regions/tissues both less pronounced and less relevant.

From our 30 selected lncRNAs measured on the microarray that present with a neuron-specific H3K4me3 mark and overlap with a SNP associated with CNS disorders, respectively 19 and 5 appear to have a highly correlated or anticorrelated expression profile with genes involved in neuronal development. As postulated by Mestdagh *et al.*, strong correlation in expression profiles is indicative for a role in the same cellular processes, implying that the lncRNAs selected here are indeed involved in networks related to neuronal development and functioning^48^. Additionally, of these 30, twenty-five candidates overlap with, or are transcribed within 5 kb from a protein-coding gene (Table 1). Among them, 17 are transcribed bidirectionally from the same promoter region as the neighbouring protein-coding gene. Interestingly, bidirectional transcription has been associated with genes exhibiting tissue-specific expression patterns^49^. Hence, it is unsurprising that a large portion of the selected lncRNAs exhibit such head-to-head transcription, as these were selected based on neuron-specific activity (predicted by neuron-specific H3K4me3). Transcription of ten out of the seventeen bidirectional candidates positively correlates with mRNA expression of the neighbouring coding gene (Table 1). This is in concordance with previous observations that bidirectionally transcribed ncRNAs show a coordinated expression with their mRNA counterpart^13^,^49^,^50^. This at least implies a coordinated regulation of both lncRNA and mRNA, and hence an involvement in the same biological processes. Moreover, this could point towards these lncRNAs functioning as transcriptional activators of the corresponding mRNA^51^,^52^. Clearly exemplifying this is the report by Boque-Sastre *et al.*, which showed that a bidirectionally transcribed lncRNA, *VIM-AS*, promotes *VIM* transcription through R-loop formation at the *VIM* TSS^52^.

**Figure 6:**
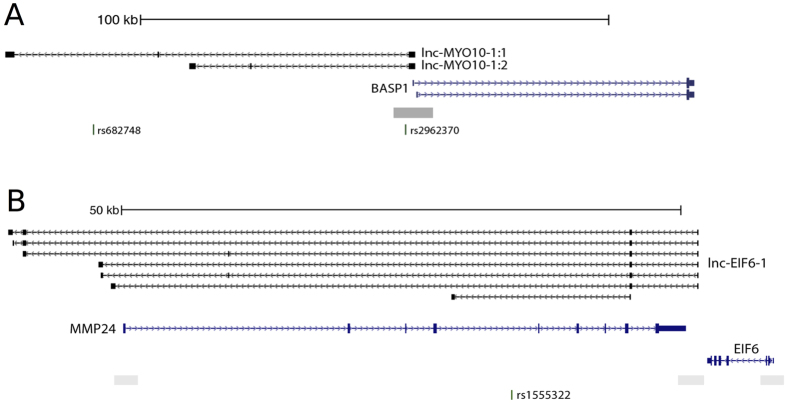
Orientation of lncRNAs relative to the nearest protein-coding gene. An example of bidirectionally transcribed and antisense overlapping lncRNAs. (**A**) lnc-MYO10-1:1 is transcribed bidirectionally to BASP1. (**B**) The lnc-EIF6-1 transcripts are transcribed in an antisense manner to MMP24 and overlap the entire MMP24 transcript body. The grey box represents the neuron-specific H3K4me3 mark. SNPs within this region are shown at the bottom.

Our bidirectionally transcribed candidates show divergent transcription to *ANKRD34C* (lnc-RASGRF1-1 transcripts), *SLC17A6* (lnc-FANCF-3:1), *NRXN1* (lnc-CHAC2-4:1), *ARNT2* (lnc-RP11-210M15.2.1-1 transcripts) & *BASP1* (lnc-MYO10-1 transcripts) (Fig. 6), suggesting a possible role in cis-regulation of these genes. The function of *ANKRD34C* is currently unknown, but for the other genes, a clear link with brain or neurodevelopment has been reported. SLC17A6 (Solute Carrier Family 17 member 6) is a vesicular glutamate transporter^53^. NRXN1 (neurexin 1) has been implicated in schizophrenia and plays an important role in the nervous system by mediating, among others, cell-cell interactions and signal transmission^54^. BASP1 (brain abundant, membrane attached signal protein 1) is specifically expressed in nervous tissue and ARNT2 (aryl-hydrocarbon receptor nuclear translocator 2) encodes a transcription factor involved in neurodevelopmental processes, neuronal connectivity and cellular responses to hypoxia^55^.

Overlapping antisense lncRNAs are traditionally associated with regulation through lncRNA-DNA or lncRNA-mRNA duplex formation, facilitating chromatin modulation, transcriptional interference, splicing inhibition, mRNA editing and stability control of the associated protein-coding gene^56^. Lnc-EIF6-1 transcripts overlap matrix metalloproteinase-24 (MMP24, a.k.a. MT5-MMP) in an antisense manner (Table 1 and Fig. 6). MMP24 is a key regulator of neural stem cell (NSC) quiescence and promotes NSC activation by cleavage of N-cadherin^57^. The two negatively correlated lnc-EIF6-1 transcripts (Table 1), suggest that lnc-EIF6-1 activation may play a role in promoting NSC quiescent state maintenance by downregulating MMP24.

In conclusion, we identified several lncRNAs putatively involved in neural development. Based on an integrated approach combining epigenetic marks, GWAS hits for CNS disorder, guilt-by-association through GSEA, correlation to ID genes and orientation relative to protein-coding genes with known function in neuronal development, several strong candidates for further functional validation were revealed. Moreover, while we did not perform an in depth analysis of the resulting sets of protein-coding genes, this strategy should also prove to be an interesting approach to identify new coding genes involved in neural development and intellectual disability.

## Methods

For all analyses regarding both coding and non-coding genes, the RefSeq coding genes catalogue (GRCh37/hg19; genome.ucsc.edu) and LNCipedia.2.1 (www.lncipedia.org) were used. The approach implemented here to select lncRNAs was also applied to RefSeq protein-coding genes as a validation strategy.

### Neuron specific histone modifications

Publically available data regarding maps of histone H3K4me3 in human brain were used. These data were generated using chromatin immunoprecipitation followed by high-throughput sequencing (ChIP-seq)^58^,^59^,^60^. Specifically, data from neuronal nuclei from the prefrontal cortex of 11 individuals and three lymphocyte control samples were used. 7947 regions, enriched in H3K4me3 in all 11 neuronal samples compared to the lymphocytes, are available through the UCSC genome browser as neuron-specific brain histone H3K4me3 peaks (UMMS Brain Hist Track; hg19)^58^. We called a neuron-specific H3K4me3 peak proximal to the promoter if it resided within 5 kb of the transcription start site (TSS).

### REST binding

ENCODE ChIP-seq data for the transcription factor REST was used through the UCSC track Integrated Regulation from ENCODE (hg19). REST ChIP-seq data from all 91 cell lines was consulted^61^,^62^,^63^. As for H3K4Me3, REST binding in the promoter region was called if, for at least one cell line, a REST peak was located within 5 kb of the TSS.

### DNAse 1 hypersensitivity

DNAse-seq data from twelve selected cell lines from the ENCODE project were used^64^,^65^. These twelve selected cell lines comprised 4 embryonic stem cell lines, 4 astrocyte cell lines, 3 neuroblastoma cell lines and 1 glioblastoma cell line. We only considered DNAse 1 hypersensitivity in the promoter region, again defined within 5 kb of the TSS in at least one of the twelve selected cell lines.

### Enrichment of ID genes and GWAS hits

Upon the identification of RefSeq coding genes and LNCipedia lncRNA transcripts featured by the epigenetic features mentioned above, these gene sets were subsequently interrogated for, respectively the presence of both ID genes and GWAS hits, and GWAS hits alone. Here we use the presence of ID genes and GWAS hits among RefSeq protein-coding genes as a validation strategy for the approach proposed here. As source of known and candidate ID genes, the gene lists from Gilissen *et al.*^7^ were used (Supplemental Table S1).

To evaluate the presence of GWAS hits, the full Catalogue of published genome wide association studies was downloaded (http://www.ebi.ac.uk/gwas/, version April 2015) and filtered for SNPs associated with central nervous system disorders such as autism, attention deficit hyperactivity disorder, bipolar disorder, epilepsy and schizophrenia, resulting in 1071 SNPs (Supplemental Table S2). A SNP is mentioned as overlapping with a coding gene or lncRNA, if it is located within the start and end position of a gene, regardless whether it is intronic or exonic.

Enrichment of the gene sets for ID genes and GWAS hits was statistically evaluated using Fisher’s exact test at the 0.05 significance level.

### Expression Profiling

Total RNA from 15 human fetal and adult tissues was obtained commercially from Stratagene Europe (Amsterdam, the Netherlands) and Agilent (Diegem, Belgium). Samples included whole brain, colon, heart, kidney, liver, lung, breast and adrenal gland (Stratagene Europe; all adult tissues); cerebellum, brain stem, striatum, frontal cortex, occipital cortex, parietal cortex (Agilent; adult tissues) and fetal whole brain (Agilent). Expression analysis was performed according to the manufacturer’s instructions with 100 ng RNA as input, using an in-house designed custom array (SurePrint G3 Human Gene Expression array v2 (AMADID 041648; Agilent Technologies, Santa Clara, CA, USA)) covering all protein coding genes and 22980 lncRNA transcripts (LNCipedia version 2.1). Normalization of these data was performed using the VSN package (v3.38) in R/Bioconductor (BioC v3.2). Normalized expression values were log2 transformed.

Differential expression analysis between brain and nonneuronal tissues was performed through Rank Product statistical analysis^66^ in R/Bioconductor, using the RankProd package (v2.42) with 1000 permutations and a false discovery rate (FDR) ≤ 0.01.

The expression data have been submitted to the GEO repository (accession number GSE81410).

### Predict putative functions of lncRNAs through guilt-by-association approach

To infer relevant biological pathways for identified lncRNAs, the microarray data were also used to create a correlation matrix by calculating Spearman’s Rank correlation coefficient for each lncRNA:mRNA pair. Subsequently, a ranked list of mRNAs was generated for each of these lncRNAs, based on the Spearman’s rho-value. These ranked lists were subsequently analyzed using preranked Gene Set Enrichment Analysis (http://www.broadinstitute.org/gsea/)^67^. GSEA was performed using the BioCarta, Kegg and Reactome Gene sets as well as the Gene Ontology gene sets from the Molecular Signature Database (MSigDB). Gene Sets with an absolute normalized enrichment score abs(NES) ≥2 and a false discovery rate FDR ≤ 0.25 were selected.

### Enrichment of ID genes among top correlated genes

For each of the selected lncRNAs, protein-coding genes with an absolute pairwise Spearman’s correlation coefficient >0.7 were used. Fisher’s exact test was used to determine enrichment of ID genes among these genes, q-values were calculated according to the method of Benjamini & Hochberg^68^. A q-value cut-off of 0.05 was used to call lncRNAs with a significant enrichment of ID genes among the top correlated genes.

### Exploration of the genomic neighbourhood of lncRNA genes

For all selected lncRNAs the nearest protein-coding gene was determined in R using the GenomicRanges package. For each overlapping protein-coding gene and the protein-coding genes that are transcribed within 5 kb of the lncRNA, a gene ontology (GO) enrichment using PANTHER^69^ was performed with visualisation using REVIGO^70^.

## Additional Information

**How to cite this article**: D’haene, E. *et al.* Identification of long non-coding RNAs involved in neuronal development and intellectual disability. *Sci. Rep.* **6**, 28396; doi: 10.1038/srep28396 (2016).

## Supplementary Material

Supplementary Information

## Figures and Tables

**Table 1:** Distance towards closest protein-coding gene for the 30 selected lncRNAs that were covered on the array.

lncRNA transcript	distance (bp)	protein-coding gene	correlation	orientation
lnc-NDUFAF2-2:1	0	SMIM15	NOT ON ARRAY	Bidirectional
lnc-RASGRF1-1:1	0	ANKRD34C	0.72	Bidirectional
lnc-RASGRF1-1:4	0	ANKRD34C	0.62	Bidirectional
lnc-RASGRF1-1:2	0	ANKRD34C	NOT SIGN	Bidirectional
lnc-RASGRF1-1:7	0	ANKRD34C	0.66	Bidirectional
lnc-RASGRF1-1:5	0	ANKRD34C	0.62	Bidirectional
lnc-RASGRF1-1:3	0	ANKRD34C	0.58	Bidirectional
lnc-RASGRF1-1:8	0	ANKRD34C	NOT SIGN	Bidirectional
lnc-RASGRF1-1:6	0	ANKRD34C	0.84	Bidirectional
lnc-MYO10-1:1	0	BASP1	0.79	Bidirectional
lnc-MYO10-1:2	0	BASP1	0.89	Bidirectional
lnc-MRE11A-1:1	0	FUT4	−0.64	Overlapping antisense
lnc-MRE11A-1:2	0	FUT4	NOT SIGN	Overlapping antisense
lnc-CHAC2-4:1	64	NRXN1	NOT SIGN	Bidirectional
lnc-C22orf32-1:2	54418	TCF20	−0.66	free-standing promoter region
lnc-C22orf32-1:4	59298	TCF20	NOT SIGN	free-standing promoter region
lnc-C22orf32-1:3	54426	TCF20	NOT SIGN	free-standing promoter region
lnc-EIF6-1:6	0	MMP24	−0.59	Overlapping antisense
lnc-EIF6-1:8	0	MMP24	NOT SIGN	Overlapping antisense
lnc-EIF6-1:11	0	MMP24	NOT SIGN	Overlapping antisense
lnc-EIF6-1:5	0	MMP24	NOT SIGN	Overlapping antisense
lnc-EIF6-1:2	0	MMP24	−0.52	Overlapping antisense
lnc-EIF6-1:7	0	MMP24	0.63	Overlapping antisense
lnc-ELF2-2:1	3112	CCRN4L	NOT SIGN	Bidirectional
lnc-RP11-210M15.2.1-1:1	236	ARNT2	0.77	Bidirectional
lnc-RP11-210M15.2.1-1:3	502	ARNT2	0.79	Bidirectional
lnc-FANCF-3:1	0	SLC17A6	NOT SIGN	Bidirectional
lnc-ANKMY1-2:1	16	CAPN10	NOT SIGN	Bidirectional
lnc-AC073043.2.1-1:2	60082	C2orf69	NOT SIGN	free-standing promoter region
lnc-AC073043.2.1-1:1	60079	C2orf69	0.61	free-standing promoter region

Also includes Spearman’s correlation coefficient (if associated protein-coding gene was covered on the array and correlation was significant) and orientation of transcription relative to the nearest protein-coding gene. NOT SIGN = not significant.
