# Supplementary material for: Identification of long non-coding RNAs involved in neuronal development and intellectual disability
Source: Sci Rep. 2016 Jun 20;6:28396. doi: 10.1038/srep28396 (PMC4913242; doi:10.1038/srep28396)
Supplement: Supplementary Information [file srep28396-s1.pdf]

Identification of long non-coding RNAs involved in neuronal development and intellectual disability

Eva D’haene, Eva Z Jacobs, Pieter-Jan Volders, Tim De Meyer, Björn Menten, Sarah Vergult

SUPPLEMENTARY INFORMATION

Supplemental Figure S1. Gene set enrichment analysis. Normalized enrichment scores (NES) of the top 10 positively and negatively enriched genes sets (abs(NES)>2 & FDR<0.25) among protein-coding genes highly correlated to the 30 selected lncRNAs that were covered on the array (except lnc-MYO10-1:1, lnc-RASGRF1-1:6, lnc-RP11-210M15.2.1-1:3 & lnc-C22orf32-1:4 that are shown in Figure 5). These results can also be found in Supplemental Table S10, which, next to the NES, also displays non-abbreviated gene set names, gene set sizes, enrichment scores and FDR q-values.

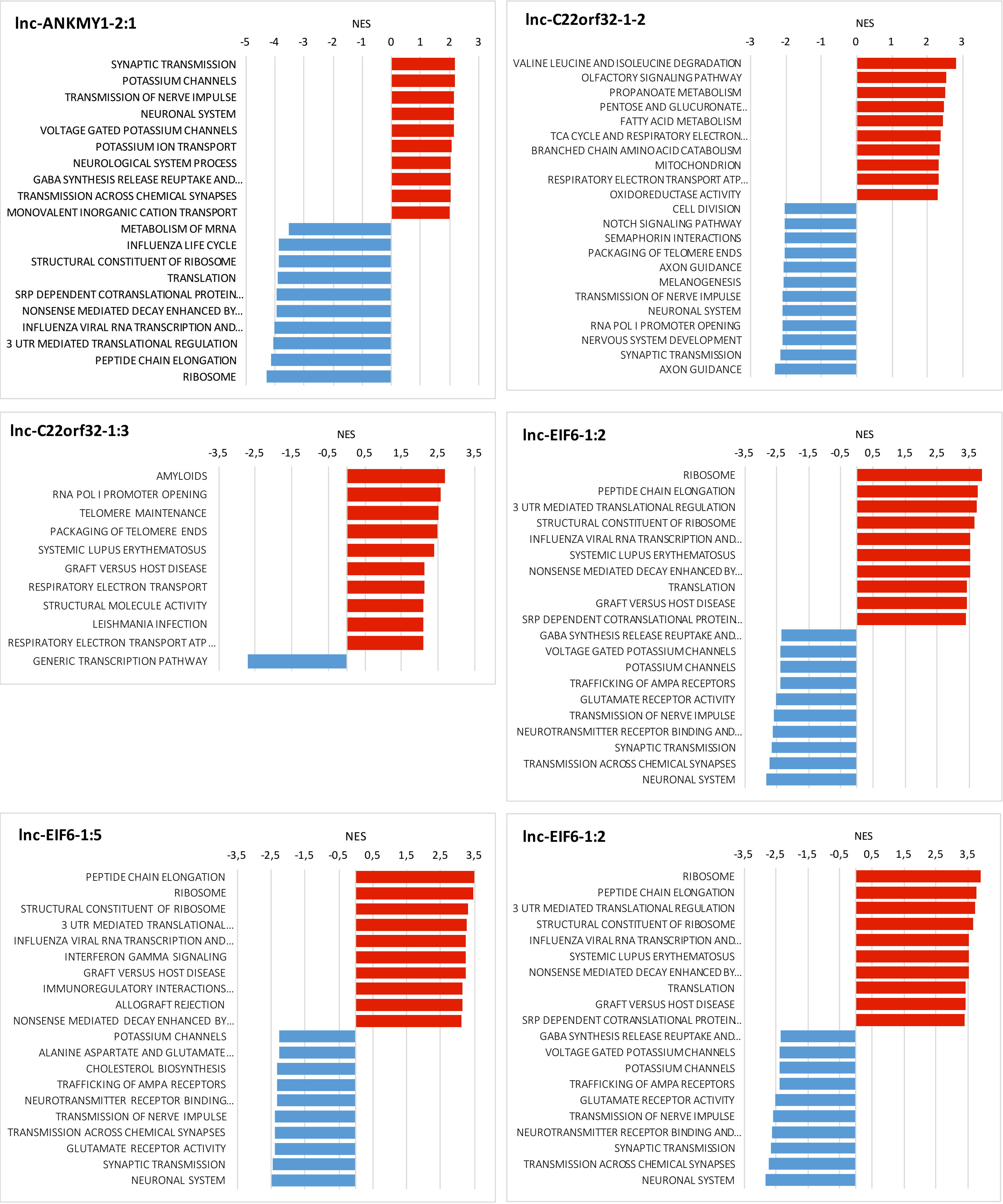

lnc-ANKMY1-2:1
NES
-5 -4 -3 -2 -1 0 1 2 3
SYNAPTIC TRANSMISSION
POTASSIUM CHANNELS
TRANSMISSION OF NERVE IMPULSE
NEURONAL SYSTEM
VOLTAGE GATED POTASSIUM CHANNELS
POTASSIUM ION TRANSPORT
NEUROLOGICAL SYSTEM PROCESS
GABA SYNTHESIS RELEASE REUPTAKE AND...
TRANSMISSION ACROSS CHEMICAL SYNAPSES
MONOVALENT INORGANIC CATION TRANSPORT
METABOLISM OF MRNA
INFLUENZA LIFE CYCLE
STRUCTURAL CONSTITUENT OF RIBOSOME
TRANSLATION
SRP DEPENDENT COTRANSLATIONAL PROTEIN...
NONSENSE MEDIATED DECAY ENHANCED BY...
INFLUENZA VIRAL RNA TRANSCRIPTION AND...
3 UTR MEDIATED TRANSLATIONAL REGULATION
PEPTIDE CHAIN ELONGATION
RIBOSOME
lnc-C22orf32-1-2
NES
-3 -2 -1 0 1 2 3
VALINE LEUCINE AND ISOLEUCINE DEGRADATION
OLFACTORY SIGNALING PATHWAY
PROPANOATE METABOLISM
PENTOSE AND GLUCURONATE...
FATTY ACID METABOLISM
TCA CYCLE AND RESPIRATORY ELECTRON...
BRANCHED CHAIN AMINO ACID CATABOLISM
MITOCHONDRION
RESPIRATORY ELECTRON TRANSPORT ATP...
OXIDOREDUCTASE ACTIVITY
CELL DIVISION
NOTCH SIGNALING PATHWAY
SEMAPHORIN INTERACTIONS
PACKAGING OF TELOMERE ENDS
AXON GUIDANCE
MELANOGENESIS
TRANSMISSION OF NERVE IMPULSE
NEURONAL SYSTEM
RNA POL I PROMOTER OPENING
NERVOUS SYSTEM DEVELOPMENT
SYNAPTIC TRANSMISSION
AXON GUIDANCE
lnc-C22orf32-1:3
NES
-3,5 -2,5 -1,5 -0,5 0,5 1,5 2,5 3,5
AMYLOIDS
RNA POL I PROMOTER OPENING
TELOMERE MAINTENANCE
PACKAGING OF TELOMERE ENDS
SYSTEMIC LUPUS ERYTHEMATOSUS
GRAFT VERSUS HOST DISEASE
RESPIRATORY ELECTRON TRANSPORT
STRUCTURAL MOLECULE ACTIVITY
LEISHMANIA INFECTION
RESPIRATORY ELECTRON TRANSPORT ATP ...
GENERIC TRANSCRIPTION PATHWAY
lnc-EIF6-1:2
NES
-3,5 -2,5 -1,5 -0,5 0,5 1,5 2,5 3,5
RIBOSOME
PEPTIDE CHAIN ELONGATION
3 UTR MEDIATED TRANSLATIONAL REGULATION
STRUCTURAL CONSTITUENT OF RIBOSOME
INFLUENZA VIRAL RNA TRANSCRIPTION AND...
SYSTEMIC LUPUS ERYTHEMATOSUS
NONSENSE MEDIATED DECAY ENHANCED BY...
TRANSLATION
GRAFT VERSUS HOST DISEASE
SRP DEPENDENT COTRANSLATIONAL PROTEIN...
GABA SYNTHESIS RELEASE REUPTAKE AND...
VOLTAGE GATED POTASSIUM CHANNELS
POTASSIUM CHANNELS
TRAFFICKING OF AMPA RECEPTORS
GLUTAMATE RECEPTOR ACTIVITY
TRANSMISSION OF NERVE IMPULSE
NEUROTRANSMITTER RECEPTOR BINDING AND...
SYNAPTIC TRANSMISSION
TRANSMISSION ACROSS CHEMICAL SYNAPSES
NEURONAL SYSTEM
lnc-EIF6-1:5
NES
-3,5 -2,5 -1,5 -0,5 0,5 1,5 2,5 3,5
PEPTIDE CHAIN ELONGATION
RIBOSOME
STRUCTURAL CONSTITUENT OF RIBOSOME
3 UTR MEDIATED TRANSLATIONAL...
INFLUENZA VIRAL RNA TRANSCRIPTION AND...
INTERFERON GAMMA SIGNALING
GRAFT VERSUS HOST DISEASE
IMMUNOREGULATORY INTERACTIONS...
ALLOGRAFT REJECTION
NONSENSE MEDIATED DECAY ENHANCED BY...
POTASSIUM CHANNELS
ALANINE ASPARTATE AND GLUTAMATE...
CHOLESTEROL BIOSYNTHESIS
TRAFFICKING OF AMPA RECEPTORS
NEUROTRANSMITTER RECEPTOR BINDING...
TRANSMISSION OF NERVE IMPULSE
TRANSMISSION ACROSS CHEMICAL SYNAPSES
GLUTAMATE RECEPTOR ACTIVITY
SYNAPTIC TRANSMISSION
NEURONAL SYSTEM
lnc-EIF6-1:2
NES
-3,5 -2,5 -1,5 -0,5 0,5 1,5 2,5 3,5
RIBOSOME
PEPTIDE CHAIN ELONGATION
3 UTR MEDIATED TRANSLATIONAL REGULATION
STRUCTURAL CONSTITUENT OF RIBOSOME
INFLUENZA VIRAL RNA TRANSCRIPTION AND...
SYSTEMIC LUPUS ERYTHEMATOSUS
NONSENSE MEDIATED DECAY ENHANCED BY...
TRANSLATION
GRAFT VERSUS HOST DISEASE
SRP DEPENDENT COTRANSLATIONAL PROTEIN...
GABA SYNTHESIS RELEASE REUPTAKE AND...
VOLTAGE GATED POTASSIUM CHANNELS
POTASSIUM CHANNELS
TRAFFICKING OF AMPA RECEPTORS
GLUTAMATE RECEPTOR ACTIVITY
TRANSMISSION OF NERVE IMPULSE
NEUROTRANSMITTER RECEPTOR BINDING AND...
SYNAPTIC TRANSMISSION
TRANSMISSION ACROSS CHEMICAL SYNAPSES
NEURONAL SYSTEM

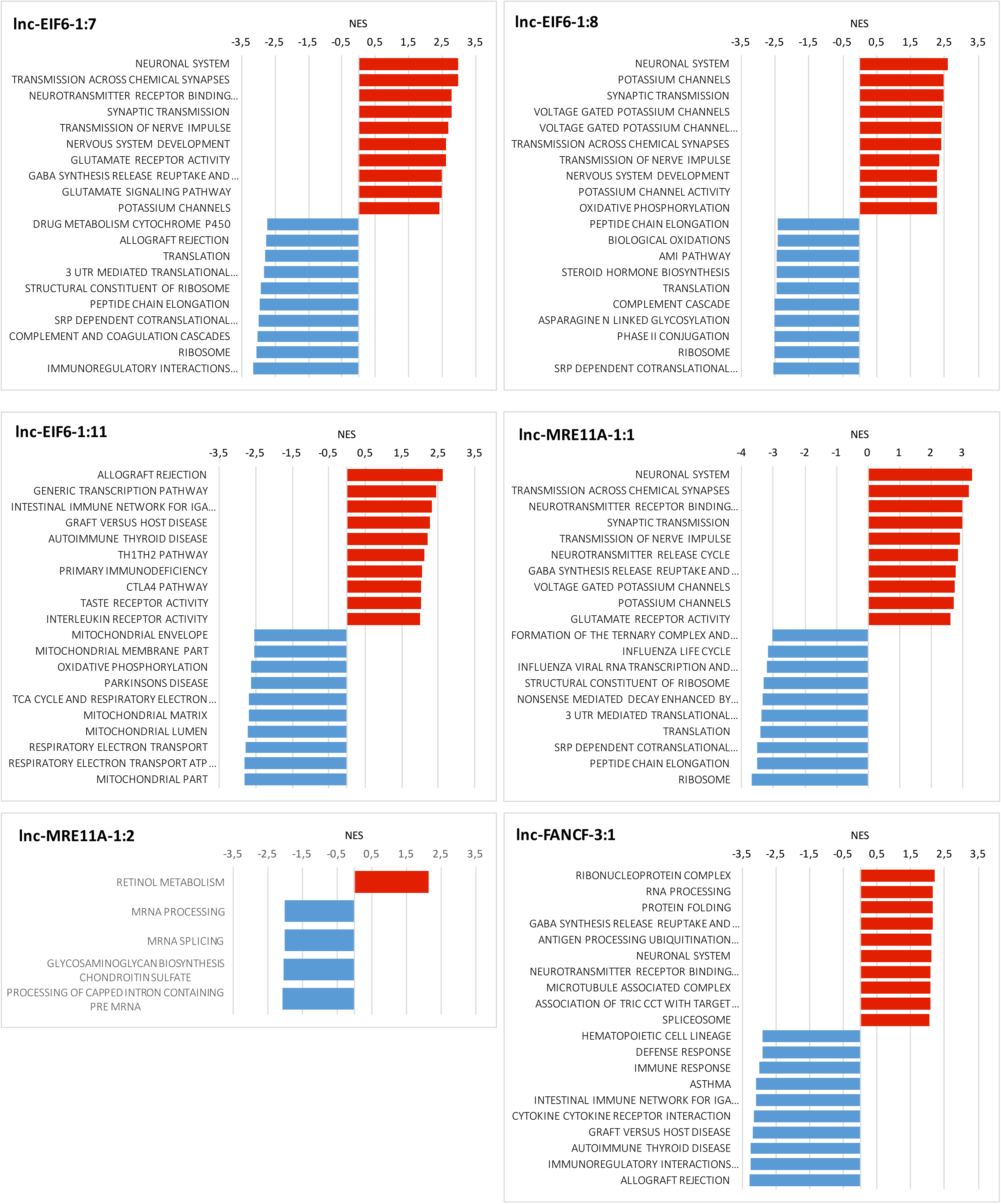
lnc-EIF6-1:7
NES
-3,5 -2,5 -1,5 -0,5 0,5 1,5 2,5 3,5
NEURONAL SYSTEM
TRANSMISSION ACROSS CHEMICAL SYNAPSES
NEUROTRANSMITTER RECEPTOR BINDING...
SYNAPTIC TRANSMISSION
TRANSMISSION OF NERVE IMPULSE
NERVOUS SYSTEM DEVELOPMENT
GLUTAMATE RECEPTOR ACTIVITY
GABA SYNTHESIS RELEASE REUPTAKE AND ...
GLUTAMATE SIGNALING PATHWAY
POTASSIUM CHANNELS
DRUG METABOLISM CYTOCHROME P450
ALLOGRAFT REJECTION
TRANSLATION
3 UTR MEDIATED TRANSLATIONAL...
STRUCTURAL CONSTITUENT OF RIBOSOME
PEPTIDE CHAIN ELONGATION
SRP DEPENDENT COTRANSLATIONAL...
COMPLEMENT AND COAGULATION CASCADES
RIBOSOME
IMMUNOREGULATORY INTERACTIONS...
lnc-EIF6-1:8
NES
-3,5 -2,5 -1,5 -0,5 0,5 1,5 2,5 3,5
NEURONAL SYSTEM
POTASSIUM CHANNELS
SYNAPTIC TRANSMISSION
VOLTAGE GATED POTASSIUM CHANNELS
VOLTAGE GATED POTASSIUM CHANNEL...
TRANSMISSION ACROSS CHEMICAL SYNAPSES
TRANSMISSION OF NERVE IMPULSE
NERVOUS SYSTEM DEVELOPMENT
POTASSIUM CHANNEL ACTIVITY
OXIDATIVE PHOSPHORYLATION
PEPTIDE CHAIN ELONGATION
BIOLOGICAL OXIDATIONS
AMI PATHWAY
STEROID HORMONE BIOSYNTHESIS
TRANSLATION
COMPLEMENT CASCADE
ASPARAGINE N LINKED GLYCOSYLATION
PHASE II CONJUGATION
RIBOSOME
SRP DEPENDENT COTRANSLATIONAL...
lnc-EIF6-1:11
NES
-3,5 -2,5 -1,5 -0,5 0,5 1,5 2,5 3,5
ALLOGRAFT REJECTION
GENERIC TRANSCRIPTION PATHWAY
INTESTINAL IMMUNE NETWORK FOR IGA...
GRAFT VERSUS HOST DISEASE
AUTOIMMUNE THYROID DISEASE
TH1TH2 PATHWAY
PRIMARY IMMUNODEFICIENCY
CTLA4 PATHWAY
TASTE RECEPTOR ACTIVITY
INTERLEUKIN RECEPTOR ACTIVITY
MITOCHONDRIAL ENVELOPE
MITOCHONDRIAL MEMBRANE PART
OXIDATIVE PHOSPHORYLATION
PARKINSONS DISEASE
TCA CYCLE AND RESPIRATORY ELECTRON ...
MITOCHONDRIAL MATRIX
MITOCHONDRIAL LUMEN
RESPIRATORY ELECTRON TRANSPORT
RESPIRATORY ELECTRON TRANSPORT ATP ...
MITOCHONDRIAL PART
lnc-MRE11A-1:1
NES
-4 -3 -2 -1 0 1 2 3
NEURONAL SYSTEM
TRANSMISSION ACROSS CHEMICAL SYNAPSES
NEUROTRANSMITTER RECEPTOR BINDING...
SYNAPTIC TRANSMISSION
TRANSMISSION OF NERVE IMPULSE
NEUROTRANSMITTER RELEASE CYCLE
GABA SYNTHESIS RELEASE REUPTAKE AND ...
VOLTAGE GATED POTASSIUM CHANNELS
POTASSIUM CHANNELS
GLUTAMATE RECEPTOR ACTIVITY
FORMATION OF THE TERNARY COMPLEX AND...
INFLUENZA LIFE CYCLE
INFLUENZA VIRAL RNA TRANSCRIPTION AND...
STRUCTURAL CONSTITUENT OF RIBOSOME
NONSENSE MEDIATED DECAY ENHANCED BY...
3 UTR MEDIATED TRANSLATIONAL...
TRANSLATION
SRP DEPENDENT COTRANSLATIONAL...
PEPTIDE CHAIN ELONGATION
RIBOSOME
lnc-MRE11A-1:2
NES
-3,5 -2,5 -1,5 -0,5 0,5 1,5 2,5 3,5
RETINOL METABOLISM
MRNA PROCESSING
MRNA SPLICING
GLYCOSAMINOGLYCAN BIOSYNTHESIS CHONDROITIN SULFATE
PROCESSING OF CAPPED INTRON CONTAINING PRE MRNA
lnc-FANCF-3:1
NES
-3,5 -2,5 -1,5 -0,5 0,5 1,5 2,5 3,5
RIBONUCLEOPROTEIN COMPLEX
RNA PROCESSING
PROTEIN FOLDING
GABA SYNTHESIS RELEASE REUPTAKE AND ...
ANTIGEN PROCESSING UBIQUITINATION...
NEURONAL SYSTEM
NEUROTRANSMITTER RECEPTOR BINDING...
MICROTUBULE ASSOCIATED COMPLEX
ASSOCIATION OF TRIC CCT WITH TARGET ...
SPLICEOSOME
HEMATOPOIETIC CELL LINEAGE
DEFENSE RESPONSE
IMMUNE RESPONSE
ASTHMA
INTESTINAL IMMUNE NETWORK FOR IGA...
CYTOKINE CYTOKINE RECEPTOR INTERACTION
GRAFT VERSUS HOST DISEASE
AUTOIMMUNE THYROID DISEASE
IMMUNOREGULATORY INTERACTIONS...
ALLOGRAFT REJECTION

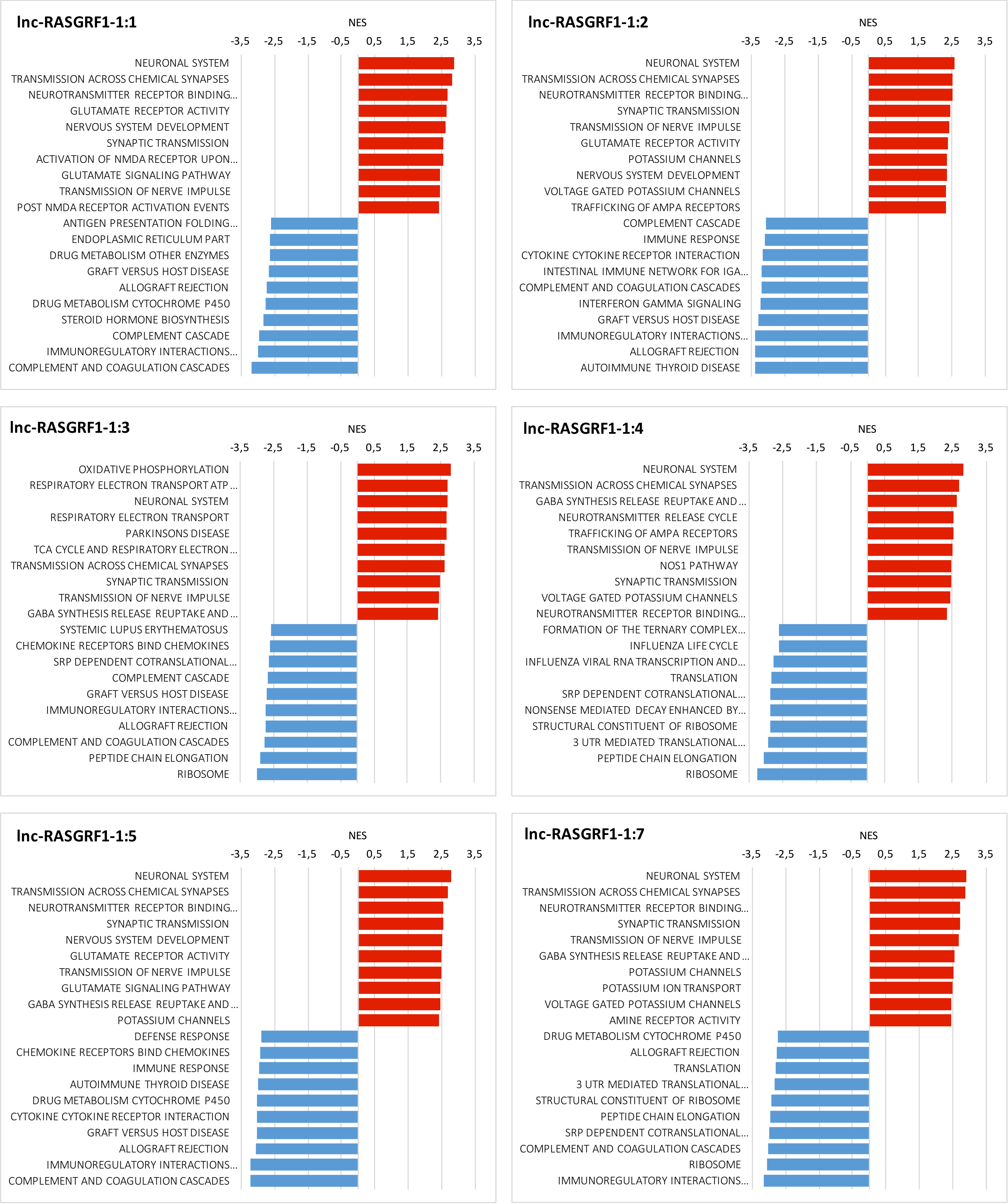
lnc-RASGRF1-1:1
NES
-3,5 -2,5 -1,5 -0,5 0,5 1,5 2,5 3,5
NEURONAL SYSTEM
TRANSMISSION ACROSS CHEMICAL SYNAPSES
NEUROTRANSMITTER RECEPTOR BINDING...
GLUTAMATE RECEPTOR ACTIVITY
NERVOUS SYSTEM DEVELOPMENT
SYNAPTIC TRANSMISSION
ACTIVATION OF NMDA RECEPTOR UPON...
GLUTAMATE SIGNALING PATHWAY
TRANSMISSION OF NERVE IMPULSE
POST NMDA RECEPTOR ACTIVATION EVENTS
ANTIGEN PRESENTATION FOLDING...
ENDOPLASMIC RETICULUM PART
DRUG METABOLISM OTHER ENZYMES
GRAFT VERSUS HOST DISEASE
ALLOGRAFT REJECTION
DRUG METABOLISM CYTOCHROME P450
STEROID HORMONE BIOSYNTHESIS
COMPLEMENT CASCADE
IMMUNOREGULATORY INTERACTIONS...
COMPLEMENT AND COAGULATION CASCADES
lnc-RASGRF1-1:2
NES
-3,5 -2,5 -1,5 -0,5 0,5 1,5 2,5 3,5
NEURONAL SYSTEM
TRANSMISSION ACROSS CHEMICAL SYNAPSES
NEUROTRANSMITTER RECEPTOR BINDING...
SYNAPTIC TRANSMISSION
TRANSMISSION OF NERVE IMPULSE
GLUTAMATE RECEPTOR ACTIVITY
POTASSIUM CHANNELS
NERVOUS SYSTEM DEVELOPMENT
VOLTAGE GATED POTASSIUM CHANNELS
TRAFFICKING OF AMPA RECEPTORS
COMPLEMENT CASCADE
IMMUNE RESPONSE
CYTOKINE CYTOKINE RECEPTOR INTERACTION
INTESTINAL IMMUNE NETWORK FOR IGA...
COMPLEMENT AND COAGULATION CASCADES
INTERFERON GAMMA SIGNALING
GRAFT VERSUS HOST DISEASE
IMMUNOREGULATORY INTERACTIONS...
ALLOGRAFT REJECTION
AUTOIMMUNE THYROID DISEASE
lnc-RASGRF1-1:3
NES
-3,5 -2,5 -1,5 -0,5 0,5 1,5 2,5 3,5
OXIDATIVE PHOSPHORYLATION
RESPIRATORY ELECTRON TRANSPORT ATP...
NEURONAL SYSTEM
RESPIRATORY ELECTRON TRANSPORT
PARKINSONS DISEASE
TCA CYCLE AND RESPIRATORY ELECTRON...
TRANSMISSION ACROSS CHEMICAL SYNAPSES
SYNAPTIC TRANSMISSION
TRANSMISSION OF NERVE IMPULSE
GABA SYNTHESIS RELEASE REUPTAKE AND...
SYSTEMIC LUPUS ERYTHEMATOSUS
CHEMOKINE RECEPTORS BIND CHEMOKINES
SRP DEPENDENT COTRANSLATIONAL...
COMPLEMENT CASCADE
GRAFT VERSUS HOST DISEASE
IMMUNOREGULATORY INTERACTIONS...
ALLOGRAFT REJECTION
COMPLEMENT AND COAGULATION CASCADES
PEPTIDE CHAIN ELONGATION
RIBOSOME
lnc-RASGRF1-1:4
NES
-3,5 -2,5 -1,5 -0,5 0,5 1,5 2,5 3,5
NEURONAL SYSTEM
TRANSMISSION ACROSS CHEMICAL SYNAPSES
GABA SYNTHESIS RELEASE REUPTAKE AND...
NEUROTRANSMITTER RELEASE CYCLE
TRAFFICKING OF AMPA RECEPTORS
TRANSMISSION OF NERVE IMPULSE
NOS1 PATHWAY
SYNAPTIC TRANSMISSION
VOLTAGE GATED POTASSIUM CHANNELS
NEUROTRANSMITTER RECEPTOR BINDING...
FORMATION OF THE TERNARY COMPLEX...
INFLUENZA LIFE CYCLE
INFLUENZA VIRAL RNA TRANSCRIPTION AND...
TRANSLATION
SRP DEPENDENT COTRANSLATIONAL...
NONSENSE MEDIATED DECAY ENHANCED BY...
STRUCTURAL CONSTITUENT OF RIBOSOME
3 UTR MEDIATED TRANSLATIONAL...
PEPTIDE CHAIN ELONGATION
RIBOSOME
lnc-RASGRF1-1:5
NES
-3,5 -2,5 -1,5 -0,5 0,5 1,5 2,5 3,5
NEURONAL SYSTEM
TRANSMISSION ACROSS CHEMICAL SYNAPSES
NEUROTRANSMITTER RECEPTOR BINDING...
SYNAPTIC TRANSMISSION
NERVOUS SYSTEM DEVELOPMENT
GLUTAMATE RECEPTOR ACTIVITY
TRANSMISSION OF NERVE IMPULSE
GLUTAMATE SIGNALING PATHWAY
GABA SYNTHESIS RELEASE REUPTAKE AND...
POTASSIUM CHANNELS
DEFENSE RESPONSE
CHEMOKINE RECEPTORS BIND CHEMOKINES
IMMUNE RESPONSE
AUTOIMMUNE THYROID DISEASE
DRUG METABOLISM CYTOCHROME P450
CYTOKINE CYTOKINE RECEPTOR INTERACTION
GRAFT VERSUS HOST DISEASE
ALLOGRAFT REJECTION
IMMUNOREGULATORY INTERACTIONS...
COMPLEMENT AND COAGULATION CASCADES
lnc-RASGRF1-1:7
NES
-3,5 -2,5 -1,5 -0,5 0,5 1,5 2,5 3,5
NEURONAL SYSTEM
TRANSMISSION ACROSS CHEMICAL SYNAPSES
NEUROTRANSMITTER RECEPTOR BINDING...
SYNAPTIC TRANSMISSION
TRANSMISSION OF NERVE IMPULSE
GABA SYNTHESIS RELEASE REUPTAKE AND...
POTASSIUM CHANNELS
POTASSIUM ION TRANSPORT
VOLTAGE GATED POTASSIUM CHANNELS
AMINE RECEPTOR ACTIVITY
DRUG METABOLISM CYTOCHROME P450
ALLOGRAFT REJECTION
TRANSLATION
3 UTR MEDIATED TRANSLATIONAL...
STRUCTURAL CONSTITUENT OF RIBOSOME
PEPTIDE CHAIN ELONGATION
SRP DEPENDENT COTRANSLATIONAL...
COMPLEMENT AND COAGULATION CASCADES
RIBOSOME
IMMUNOREGULATORY INTERACTIONS...

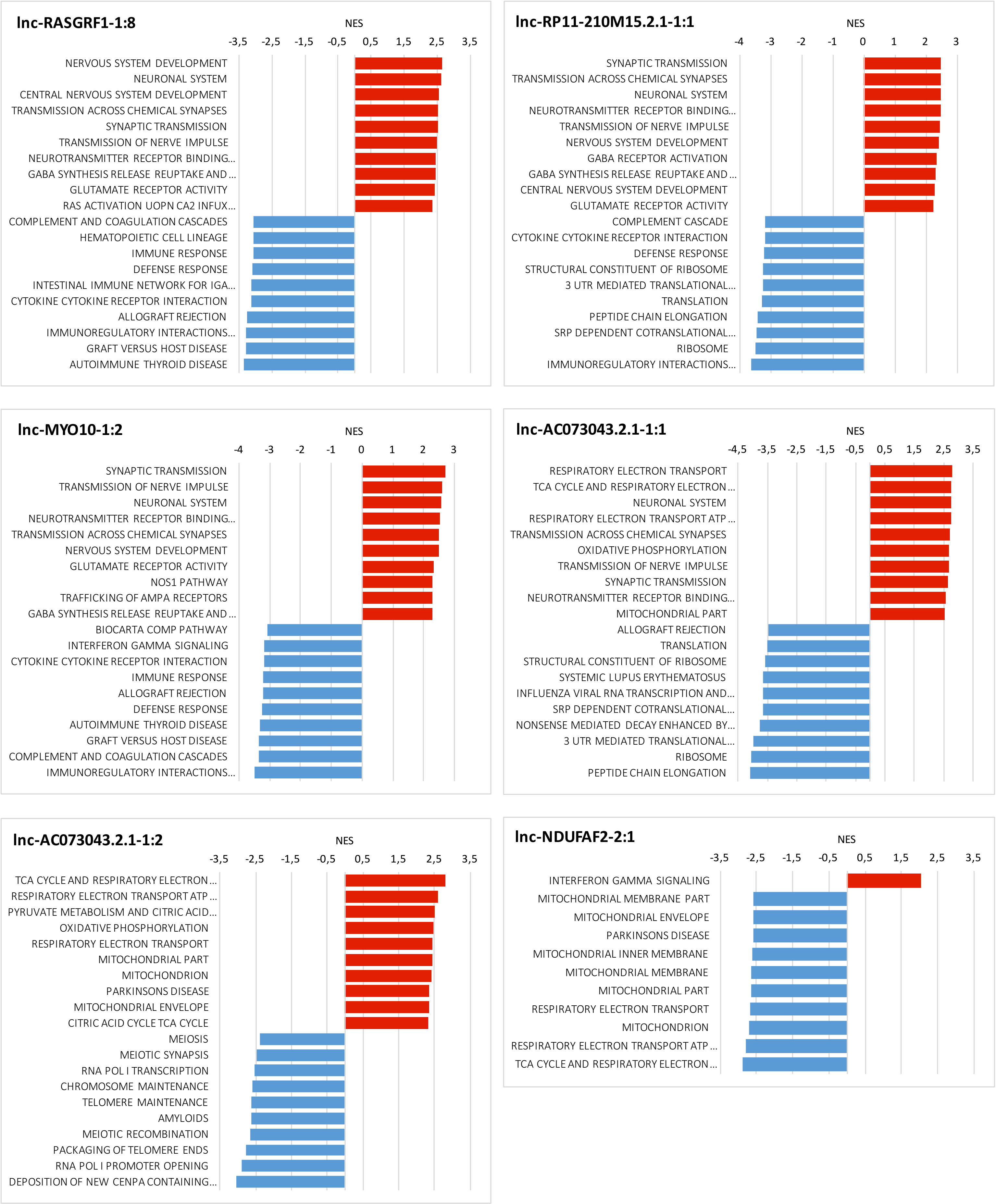
lnc-RASGRF1-1:8
NES
-3,5 -2,5 -1,5 -0,5 0,5 1,5 2,5 3,5
NERVOUS SYSTEM DEVELOPMENT
NEURONAL SYSTEM
CENTRAL NERVOUS SYSTEM DEVELOPMENT
TRANSMISSION ACROSS CHEMICAL SYNAPSES
SYNAPTIC TRANSMISSION
TRANSMISSION OF NERVE IMPULSE
NEUROTRANSMITTER RECEPTOR BINDING...
GABA SYNTHESIS RELEASE REUPTAKE AND ...
GLUTAMATE RECEPTOR ACTIVITY
RAS ACTIVATION UOPN CA2 INFUX...
COMPLEMENT AND COAGULATION CASCADES
HEMATOPOIETIC CELL LINEAGE
IMMUNE RESPONSE
DEFENSE RESPONSE
INTESTINAL IMMUNE NETWORK FOR IGA...
CYTOKINE CYTOKINE RECEPTOR INTERACTION
ALLOGRAFT REJECTION
IMMUNOREGULATORY INTERACTIONS...
GRAFT VERSUS HOST DISEASE
AUTOIMMUNE THYROID DISEASE
lnc-RP11-210M15.2.1-1:1
NES
-4 -3 -2 -1 0 1 2 3
SYNAPTIC TRANSMISSION
TRANSMISSION ACROSS CHEMICAL SYNAPSES
NEURONAL SYSTEM
NEUROTRANSMITTER RECEPTOR BINDING...
TRANSMISSION OF NERVE IMPULSE
NERVOUS SYSTEM DEVELOPMENT
GABA RECEPTOR ACTIVATION
GABA SYNTHESIS RELEASE REUPTAKE AND ...
CENTRAL NERVOUS SYSTEM DEVELOPMENT
GLUTAMATE RECEPTOR ACTIVITY
COMPLEMENT CASCADE
CYTOKINE CYTOKINE RECEPTOR INTERACTION
DEFENSE RESPONSE
STRUCTURAL CONSTITUENT OF RIBOSOME
3 UTR MEDIATED TRANSLATIONAL...
TRANSLATION
PEPTIDE CHAIN ELONGATION
SRP DEPENDENT COTRANSLATIONAL...
RIBOSOME
IMMUNOREGULATORY INTERACTIONS...
lnc-MYO10-1:2
NES
-4 -3 -2 -1 0 1 2 3
SYNAPTIC TRANSMISSION
TRANSMISSION OF NERVE IMPULSE
NEURONAL SYSTEM
NEUROTRANSMITTER RECEPTOR BINDING...
TRANSMISSION ACROSS CHEMICAL SYNAPSES
NERVOUS SYSTEM DEVELOPMENT
GLUTAMATE RECEPTOR ACTIVITY
NOS1 PATHWAY
TRAFFICKING OF AMPA RECEPTORS
GABA SYNTHESIS RELEASE REUPTAKE AND ...
BIOCARTA COMP PATHWAY
INTERFERON GAMMA SIGNALING
CYTOKINE CYTOKINE RECEPTOR INTERACTION
IMMUNE RESPONSE
ALLOGRAFT REJECTION
DEFENSE RESPONSE
AUTOIMMUNE THYROID DISEASE
GRAFT VERSUS HOST DISEASE
COMPLEMENT AND COAGULATION CASCADES
IMMUNOREGULATORY INTERACTIONS...
lnc-AC073043.2.1-1:1
NES
-4,5 -3,5 -2,5 -1,5 -0,5 0,5 1,5 2,5 3,5
RESPIRATORY ELECTRON TRANSPORT
TCA CYCLE AND RESPIRATORY ELECTRON ...
NEURONAL SYSTEM
RESPIRATORY ELECTRON TRANSPORT ATP ...
TRANSMISSION ACROSS CHEMICAL SYNAPSES
OXIDATIVE PHOSPHORYLATION
TRANSMISSION OF NERVE IMPULSE
SYNAPTIC TRANSMISSION
NEUROTRANSMITTER RECEPTOR BINDING...
MITOCHONDRIAL PART
ALLOGRAFT REJECTION
TRANSLATION
STRUCTURAL CONSTITUENT OF RIBOSOME
SYSTEMIC LUPUS ERYTHEMATOSUS
INFLUENZA VIRAL RNA TRANSCRIPTION AND...
SRP DEPENDENT COTRANSLATIONAL...
NONSENSE MEDIATED DECAY ENHANCED BY...
3 UTR MEDIATED TRANSLATIONAL...
RIBOSOME
PEPTIDE CHAIN ELONGATION
lnc-AC073043.2.1-1:2
NES
-3,5 -2,5 -1,5 -0,5 0,5 1,5 2,5 3,5
TCA CYCLE AND RESPIRATORY ELECTRON ...
RESPIRATORY ELECTRON TRANSPORT ATP ...
PYRUVATE METABOLISM AND CITRIC ACID...
OXIDATIVE PHOSPHORYLATION
RESPIRATORY ELECTRON TRANSPORT
MITOCHONDRIAL PART
MITOCHONDRION
PARKINSONS DISEASE
MITOCHONDRIAL ENVELOPE
CITRIC ACID CYCLE TCA CYCLE
MEIOSIS
MEIOTIC SYNAPSIS
RNA POL I TRANSCRIPTION
CHROMOSOME MAINTENANCE
TELOMERE MAINTENANCE
AMYLOIDS
MEIOTIC RECOMBINATION
PACKAGING OF TELOMERE ENDS
RNA POL I PROMOTER OPENING
DEPOSITION OF NEW CENPA CONTAINING...
lnc-NDUFAF2-2:1
NES
-3,5 -2,5 -1,5 -0,5 0,5 1,5 2,5 3,5
INTERFERON GAMMA SIGNALING
MITOCHONDRIAL MEMBRANE PART
MITOCHONDRIAL ENVELOPE
PARKINSONS DISEASE
MITOCHONDRIAL INNER MEMBRANE
MITOCHONDRIAL MEMBRANE
MITOCHONDRIAL PART
RESPIRATORY ELECTRON TRANSPORT
MITOCHONDRION
RESPIRATORY ELECTRON TRANSPORT ATP ...
TCA CYCLE AND RESPIRATORY ELECTRON ...

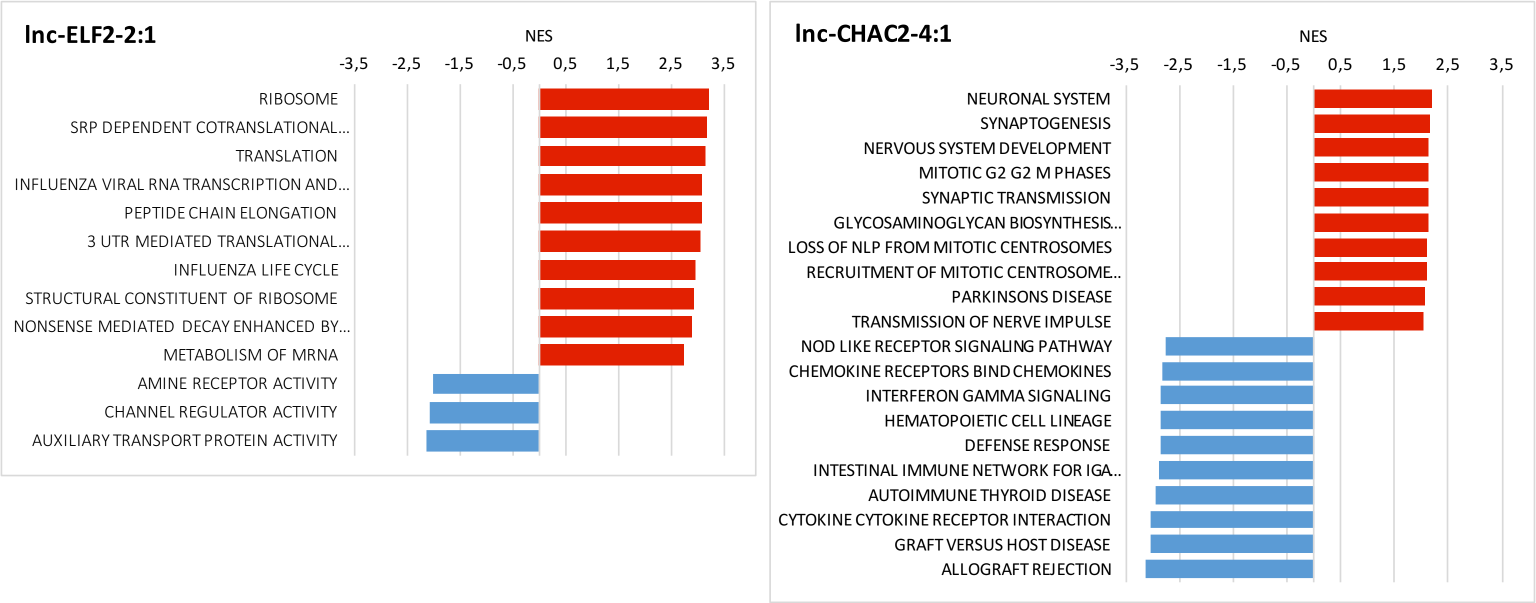

lnc-ELF2-2:1
NES
-3,5 -2,5 -1,5 -0,5 0,5 1,5 2,5 3,5
RIBOSOME
SRP DEPENDENT COTRANSLATIONAL...
TRANSLATION
INFLUENZA VIRAL RNA TRANSCRIPTION AND...
PEPTIDE CHAIN ELONGATION
3 UTR MEDIATED TRANSLATIONAL...
INFLUENZA LIFE CYCLE
STRUCTURAL CONSTITUENT OF RIBOSOME
NONSENSE MEDIATED DECAY ENHANCED BY...
METABOLISM OF MRNA
AMINE RECEPTOR ACTIVITY
CHANNEL REGULATOR ACTIVITY
AUXILIARY TRANSPORT PROTEIN ACTIVITY
lnc-CHAC2-4:1
NES
-3,5 -2,5 -1,5 -0,5 0,5 1,5 2,5 3,5
NEURONAL SYSTEM
SYNAPTOGENESIS
NERVOUS SYSTEM DEVELOPMENT
MITOTIC G2 G2 M PHASES
SYNAPTIC TRANSMISSION
GLYCOSAMINOGLYCAN BIOSYNTHESIS...
LOSS OF NLP FROM MITOTIC CENTROSOMES
RECRUITMENT OF MITOTIC CENTROSOME...
PARKINSONS DISEASE
TRANSMISSION OF NERVE IMPULSE
NOD LIKE RECEPTOR SIGNALING PATHWAY
CHEMOKINE RECEPTORS BIND CHEMOKINES
INTERFERON GAMMA SIGNALING
HEMATOPOIETIC CELL LINEAGE
DEFENSE RESPONSE
INTESTINAL IMMUNE NETWORK FOR IGA...
AUTOIMMUNE THYROID DISEASE
CYTOKINE CYTOKINE RECEPTOR INTERACTION
GRAFT VERSUS HOST DISEASE
ALLOGRAFT REJECTION

Supplemental Figure S2. Genomic neighbourhood of 4188 lncRNAs featured by neuron-specific H3K4me3. Enriched GO terms of neighbouring (transcribed within 5 kb) or overlapping protein-coding genes. A forebrain development cluster is clearly represented here (brown).

REVIGO Gene Ontology treemap

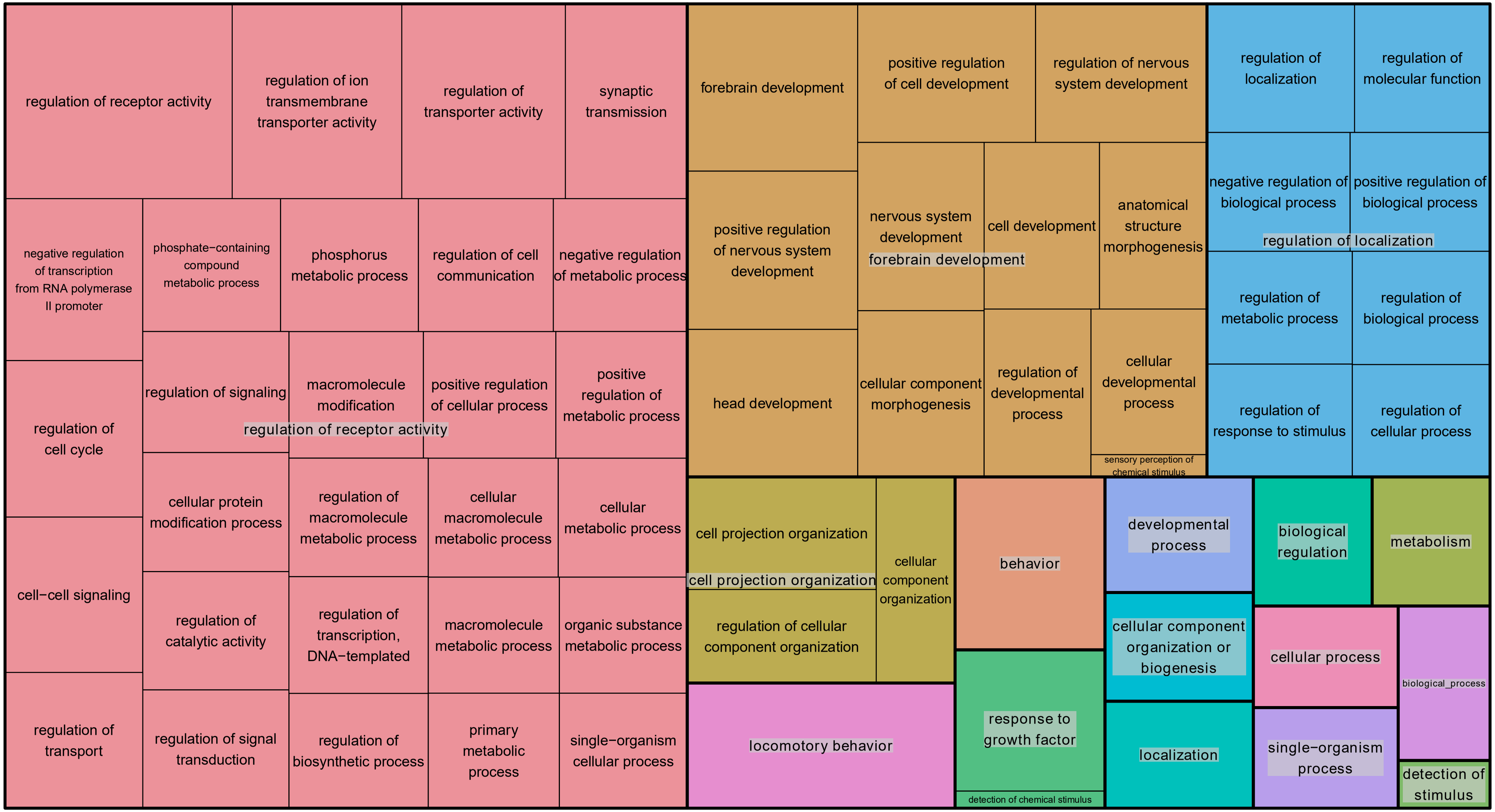

Supplemental Table S1. List of known and candidate ID genes

Known ID genes

ABCC9	B3GALTL	DCX	GALE	KCNJ10	MTRR	PEX1	RYR1	SUOX
ABCD1	BBS1	DDHD2	GALT	KCNJ11	MUT	PEX10	RYR2	SURF1
ABCD4	BBS10	DHCR24	GAMT	KCNQ2	MVK	PEX13	SALL1	SYN1
ABHD5	BBS12	DHCR7	GATM	KCNQ3	MYCN	PEX26	SATB2	SYNE1
ACAD9	BBS2	DISC1	GCH1	KCNT1	MYH9	PEX5	SBF1	SYNGAP1
ACOX1	BBS4	DKC1	GDI1	KCTD7	MYO1E	PEX7	SCN1A	SYP
ACSF3	BBS5	DLD	GFAP	KDM5C	MYO3A	PGAP2	SCN2A	TAT
ACTB	BBS7	DLG3	GJC2	KDM6A	MYT1L	PGK1	SCN8A	TBC1D24
ACTG1	BBS9	DMD	GK	KIAA1279	NAGA	PHF21A	SCO2	TBR1
ACVR1	BCKDHA	DNAH5	GLDC	KIF11	NAGLU	PHF6	SDHA	TCF4
ADAR	BCKDHB	DNMT3B	GLI2	KIF7	NBN	PHF8	SERAC1	TFAP2A
ADCK3	BCOR	DOCK8	GLI3	KIRREL3	NDE1	PHGDH	SETBP1	TGFBR1
ADSL	BCS1L	DPAGT1	GLRA1	KIT	NDP	PIGV	SHANK2	TGFBR2
AFF2	BLM	DPM1	GMPPB	KNCQ2	NDUFS1	PLCB1	SHANK3	TGIF1
AGA	BRAF	DPYD	GNAS	KRAS	NDUFS2	PLP1	SHH	THRB
AGPAT2	BSCL2	DYM	GNPAT	L1CAM	NDUFS4	PMM2	SHOC2	TIMM8A
AGTR2	BUB1B	DYNC1H1	GNRHR	L2HGDH	NDUFS8	PNKP	SHOX	TMEM237
AHCY	C5orf42	DYRK1A	GNS	LAMA2	NDUFV1	PNP	SHROOM4	TMEM67
AHI1	MPLKIP	EFTUD2	GP1BB	LAMC3	NEDD4L	POLG	SIL1	TNK2
AK1	CA2	EHMT1	GPC3	LAMP2	NEU1	POLR3A	SIX3	TOR1A
AKT3	CACNA1A	EIF2AK3	GPHN	LARGE	NF1	POLR3B	SKI	TPK1
ALDH18A1	CACNA1C	EIF4G1	GPR56	LCT	NFIA	POMGNT1	SLC12A6	TPO
ALDH3A2	CASK	ELOVL4	GRIA3	LIG4	NFIX	POMT1	SLC16A2	TRAPPC9
ALDH5A1	CBS	EP300	GRIN2A	LRP1	NHS	POMT2	SLC17A5	TREX1
ALG1	CC2D2A	EPHA5	GRIN2B	LRP2	NIPBL	PORCN	SLC25A15	TRIM32
ALG12	CCBE1	EPHB2	GRM1	MAN2B1	NKX2-1	POU1F1	SLC26A9	TRPM6
ALG3	CCDC22	ERBB4	GSS	MANBA	NLGN1	POU3F4	SLC2A1	TSC1
ALG6	CCDC39	ERCC2	GUSB	MAP2K1	NLGN4X	PPM1D	SLC33A1	TSC2
AMT	CDH15	ERCC3	HAX1	MAP2K2	NLRP3	PPOX	SLC35C1	TSPAN7
ANK2	CDK5RAP2	ERCC5	HCCS	MAPT	NOG	PRODH	SLC4A4	TTC8
ANK3	CDKL5	ERCC6	HCFC1	MAT1A	NOTCH3	PRPS1	SLC6A3	TTN
ANKH	CDON	ERCC8	HDAC4	MBD5	NPHP1	PSEN1	SLC6A8	TUBA1A
ANKRD11	CELSR1	ESCO2	HDAC8	MBTPS2	NR0B1	PTCH1	SLC7A7	TUBB2B
ANO5	CENPJ	ETFB	HESX1	MCCC1	NR4A2	PTCHD1	SLC9A6	TUSC3
ANTXR1	CEP152	ETHE1	HLCS	MCCC2	NRXN1	PTDSS1	SMAD4	TWIST1
AP1S2	CEP290	EXOSC3	HMGA2	MCOLN1	nrxn1b	PTEN	SMARCA2	UBE2A
AP3B1	CEP41	EXT1	HNRNPU	MCPH1	NSD1	PTPN11	SMARCA4	UBE3A
APAF1	CHAT	AMER1	HOXA1	MECP2	NSDHL	PVRL1	SMARCB1	UBR1
APTX	CHD2	FANCB	HPD	mecp2e1	NSUN2	PYCR1	SMARCE1	UPB1
ARFGEF2	CHD7	FANCD2	HPRT1	MED12	NTRK1	RAB18	SMC1A	UPF3B
ARHGEF9	CHKB	FBN1	HRAS	MEF2C	OBSL1	RAB27A	SMOC1	VLDLR
ARID1A	CHRND	FGD1	HSD17B10	MGAT2	OCA2	RAB3GAP1	SMPD1	VPS13B
ARID1B	CLCN7	FGFR2	HUWE1	MID1	OCLN	RAB3GAP2	SMS	VPS39
ARL6	CLN8	FGFR3	IDS	MKKS	OCRL	RAF1	SNAP29	WDR11
ARSE	CNTNAP2	FH	IDUA	MLH1	OFD1	RAI1	SOS1	WDR19
ARX	COG8	FKRP	IFT172	KMT2A	OPHN1	RARS2	SOX10	WDR45
ASL	COL4A1	FKTN	IGF1	KMT2D	ORC1	RASGEF1B	SOX2	WDR62
ASPA	COL4A2	FLG	IKBKG	KMT2C	OTC	RBFOX1	SOX3	XPA

ASPM	COLEC11	FLNA	IL1RAPL1	MLYCD	PAFAH1B1	RFT1	SOX5	YWHAE
ASXL1	COQ2	FMR1	INPP5E	MMAA	PAK3	RFX3	SPG7	ZDHHC9
ATP1A2	CREBBP	FOXG1	IQSEC2	MMACHC	PANK2	RNASEH2A	SPRED1	ZEB2
ATP2A2	CTNNB1	FOXP1	IRS1	MMADHC	PARP1	RNASEH2B	SPTAN1	ZFHX4
ATP6V0A2	CUBN	FOXP2	ISPD	MOCS1	PAX6	RNASEH2C	SRCAP	ZFYVE26
ATP7A	CUL3	FRAS1	ITGB3	MOCS2	PC	ROGDI	SRD5A3	ZIC2
ATR	CUL4B	FTO	ITPR1	MPDU1	PCDH19	RP2	SRGAP3	ZNF41
ATRX	CYB5R3	FTSJ1	KANK1	MPDZ	PCNT	RPGRIP1L	STRA6	ZNF674
ATXN1	D2HGDH	FUCA1	KANSL1	MSH6	PDE4D	RPS6KA3	STS	
AUH	DARS2	GABRA1	KAT6B	MTMR2	PDHA1	RTEL1	STXBP1	
AUTS2	DBT	GABRB3	KCNA1	MTR	PEPD	RUNX1	SUCLA2	

Candidate ID genes

ABCA2	CDS2	GAD1	MED23	PTPRM	SZT2	BRWD3	KCNB1	RNF38
ABCG4	CELF2	GAS2	MEGF11	PTPRR	TAAR2	BTN1A1	KCNH1	RREB1
ABI3BP	CEP135	GATAD2B	MEOX2	PTPRT	TMLHE	C10orf11	KCNK12	RRP1B
ACACB	CHAMP1	GCSH	METTL14	PUS1	TMPRSS12	C11orf46	KCNK9	RTN4RL1
ACBD6	CHD1	GIMAP8	MGAT4C	PWWP2A	TNKS2	C12orf57	KDM1A	RUNX1T1
ACO2	CHD3	GNAO1	MIB1	RAB2A	TNPO2	C12orf65	KDM1B	RUVBL1
ACSL4	CHD6	GOLGA3	MKLN1	RAB33B	TNR	C15orf38	KDM2B	RXFP1
ADAT3	CHL1	GON4L	MLL4	RAB39B	TOP1	C15orf62	KDM5A	S100G
ADCY7	CHRNA7	GPD2	MLL5	RAB40AL	TRAK1	CNTN5	KDM5B	SAP30BP
ADK	CIC	GPR115	MMP27	RAB5C	TRAPPC11	COG1	KDM6B	SC5DL
ADNP	CIT	GPR153	NDUFA1	RAD21	TRIM29	COG6	KIAA0100	SCAF4
ADRA2B	CLIC2	GPR84	NDUFA11	RAD21L1	TRIM8	COG7	KIAA0182	SCP2
AGPAT3	CLK2	GPS1	NDUFA12	RALGAPB	TRIO	COL25A1	KIAA0232	SDCBP2
AIFM1	CNKSR1	GRB14	NDUFS3	RALGDS	TRIP12	COL4A3BP	MMP8	SEMA4G
AIMP1	CNKSR2	GRIA1	NDUFS7	RALGPS1	TRMT1	COLEC12	MOB4	SETD5
AKR1C4	CNOT1	GRIA2	NFASC	RAPGEF1	TRMT10A	COQ5	MPHOSPH8	SFPQ
ALG2	CNOT3	GRIK2	NGEF	RARG	TRPC5	COX15	MPP6	SLC16A3
ALG9	CNOT4	GRIN1	NISCH	RARS	TRPM3	CRADD	MRPS22	SLC1A2
ALS2CL	DDX11	GRM5	NLGN2	RASGRP1	TRPM5	CRBN	MRS2	TAF1
ALX1	DDX20	GRM7	NLRC5	RASIP1	TRPM7	CRK	MTF1	TAF2
AMY2B	DDX3X	GSPT2	NOTUM	RB1CC1	TRRAP	CRTAC1	MTMR12	TAF7L
ANKRD12	DDX50	GTF2H5	NPAS4	RBM10	TSPAN17	CSDE1	MTOR	TANC2
ANO10	DEAF1	GTPBP8	NPRL2	RBM28	TSPYL5	CSNK1E	MYH10	TBC1D14
AP2A2	DENR	H2AFV	NR2F1	RBMS3	TTI2	CTCF	MYO5A	TBC1D7
AP3B2	DGCR14	HARS	NRN1	RECK	TUBGCP6	CTDP1	MYO7B	TBCE
AP3M1	DGCR2	HDGFRP2	NTNG1	RELN	UBE3B	CTTNBP2	MYOF	TBL1XR1
AP4B1	DGKH	HDLBP	NUAK1	REPS2	UBE3C	CTTNBP2NL	MYT1	TBX18
AP4E1	DHDDS	HDX	NUP54	REST	UBN2	CUL5	N6AMT1	TCF7L1
AP4S1	DHFR	HECTD1	NXF5	SLC25A22	UBQLN1	CUX2	NAA10	TECPR2
APH1A	DHRS4L1	HERC2	OMG	SLC25A39	UBR3	CYP4F3	NAA40	TECR
ARHGAP30	DHTKD1	HIPK3	OPLAH	SLC25A5	UBR5	CYTH1	NACA	TFDP1
ARHGEF10	DIAPH3	HIST1H1E	OPRL1	SLC31A1	UBR7	DBR1	NALCN	TGM3
ARHGEF6	DIP2B	HIST1H2AE	OR10S1	SLC35A2	UBTF	DCAF4	NAPRT1	THBS1

ARIH1	DIP2C	HIST1H2AG	OR5M1	SLC4A10	UGGT1	ELP2	NAV2	THOC2
ARL13B	DLG1	HIST3H3	OSBPL5	SLC4A8	UNC13C	EMILIN3	NBEA	THOC6
ARMC9	DLG4	HIVEP2	PACS1	SLC6A1	UNC80	EMX2	NCAPD2	TLK2
ASAH2	DLL1	KIAA0317	PACS2	SLC6A13	UPF2	ENTPD1	NCKAP1	TMC4
ASB1	DLX3	KIAA1033	PAFAH1B3	SMARCC2	URB2	EPB41L1	NDST1	TMCO1
ASCC3	DMPK	KIAA1109	PALLD	SMC3	USP15	EPC2	PECR	TMEM135
ASCL1	DNAH17	KIAA1324L	PAX1	SMG9	USP46	ERLIN2	PEX11B	TMEM165
ASH1L	DNAH7	KIAA1462	PAX5	SMURF1	UTP14C	ESAM	PGRMC1	TMEM231
ASNS	DNAJB9	KIAA2018	PBRM1	SNIP1	VCX3A	EXOC6B	PHACTR1	TMEM41A
ATP10D	DNAJC19	KIAA2022	PCDH11X	SNX3	VPS37A	EYA4	PHF19	TMEM85
ATP12A	DNAJC6	KIF14	PCDH18	SOBP	WAC	FAAH2	PHF2	WDR45L
C16orf62	DNM1	KIF1A	PCDHA13	SP7	WDFY3	FAM102A	PHIP	WDR62
C17orf53	DNMT3A	KIF5C	PCDHB13	SPAG17	WDR13	FAM116B	PIAS1	WHSC1L1
C1QTNF6	DOCK9	KLF12	PCDHB4	SPATA13	ZNF480	FAM129B	PIGL	XPO1
C6orf174	DPP3	KLF8	PCNX	SPATA5	ZNF526	FAM13C	PIGN	XPO5
C7orf43	DPP7	KLHL11	PCOLCE	SRBD1	ZNF565	FAM45A	PIGO	XPR1
C9orf86	DRD4	KPNA1	PDCD1	SRPR	ZNF592	FAM63B	PIGT	XYLT1
CA8	DST	KRT80	PDIA6	SRPX2	ZNF673	FAM8A1	PIK3R2	YWHAG
CACNA1E	DUS1L	LAMA1	PDIK1L	SSBP3	ZNF711	FAM91A1	PIK3R3	YY1
CACNA1G	DUSP15	LARP7	PDSS1	ST3GAL3	ZNF81	FASN	PIWIL4	ZBTB40
CACNG2	EBAG9	LINS	PDSS2	ST3GAL5	ATP1B1	FASTKD5	PLA1A	ZBTB41
CAMK2G	EDA2R	LRPPRC	PPP2R2C	ST3GAL6	ATP2B4	FAT1	PLCL2	ZC3H12B
CAP1	EEF1A2	LTN1	PPP2R5D	ST5	ATP6AP2	FBXW9	PLP2	ZC3H14
CARKD	EEF1B2	LZTR1	PQBP1	STAG1	ATP8A2	FCRL6	PNPLA7	ZCCHC12
CASP2	EFHC2	MADD	PRDM12	STAP2	B3GNT4	FETUB	POC1A	ZCCHC8
CC2D1A	EFR3A	MAGEL2	PRDX6	STIL	B4GALT1	HTR7	POGZ	ZDHHC15
CCDC137	EIF2C1	MAGT1	PRKCA	STK36	B4GALT7	IER3IP1	POLR2A	ZFHX3
CCDC18	EIF2S3	MAN1B1	PRKCB	STT3A	BCAP31	IFT81	POLR2M	ZMYM3
CCDC78	ELK1	MAOA	PRKRA	STT3B	BCORL1	IGBP1	POLRMT	ZMYM6
CCDC8	FGD3	MAPK10	PRMT10	STX1B	BEST3	inpp4a	PPP1R15B	ZNF238
CCDC84	FGF22	MAPK8IP1	PROX2	STXBP3	BIRC6	IQGAP2	RGMA	ZNF292
CCNT1	FLVCR1	MBTPS1	PRPF39	SUPT16H	BMP1	IQSEC1	RGS14	ZNF385B
CDC42BPB	FMN2	MCAM	PRSS12	SV2B	BRAT1	ITGA5	RGS7	ZNF407
CDK11A	FREM3	MCM3AP	PSMA7	SVIL	BRD4	JAM3	RIMS1	ZNF44
CDK5R1	FRMPD4	MED13	PSMG4	SYNCRIP	BRPF1	JARID2	RIMS2	ZNF451
CDK6	FRY	MED13L	PTGR1	SYNRG	BRSK2	KANSL2	RIOK3	
CDKL3	GABRB1	MED17	PTPRK	SYT14	BRWD1	KATNAL2	RMND1	

Supplemental Table S2. Overview of selected GWAS hits associated with CNS disorders

chrom	*chromStart*	*chromEnd*	*ID*	*chrom*	*chromStart*	*chromEnd*	*ID*
chr1	30431559	30431560	rs1009080	chr1	30525713	30525714	rs2860031
chr1	218705813	218705814	rs1018040	chr1	61059258	61059259	rs2989476
chr1	244390563	244390564	rs10429924	chr1	88226328	88226329	rs35214987
chr1	167903078	167903079	rs10489202	chr1	115052707	115052708	rs3827735
chr1	115263684	115263685	rs10489525	chr1	24431955	24431956	rs3934861
chr1	234583617	234583618	rs10489896	chr1	61815798	61815799	rs41350144
chr1	73824908	73824909	rs10789369	chr1	61824388	61824389	rs41453448
chr1	183852913	183852914	rs10797919	chr1	69579251	69579252	rs4147141
chr1	115073875	115073876	rs10858047	chr1	115875644	115875645	rs4332358
chr1	79263804	79263805	rs10873998	chr1	59325625	59325626	rs4601609
chr1	186632316	186632317	rs10911902	chr1	79238014	79238015	rs4650608
chr1	245733607	245733608	rs10924245	chr1	41839821	41839822	rs4660531
chr1	115040931	115040932	rs11102800	chr1	61095557	61095558	rs472913
chr1	115061583	115061584	rs11102807	chr1	11788563	11788564	rs4846033
chr1	74339166	74339167	rs11210359	chr1	61822282	61822283	rs4915737
chr1	61831889	61831890	rs1125777	chr1	30432218	30432219	rs4949526
chr1	160630142	160630143	rs11265461	chr1	34186192	34186193	rs528059
chr1	224538939	224538940	rs11579964	chr1	34189428	34189429	rs544991
chr1	114959539	114959540	rs11582563	chr1	147083113	147083114	rs583583
chr1	114973689	114973690	rs11585926	chr1	37162351	37162352	rs589249
chr1	115079657	115079658	rs11587400	chr1	193921547	193921548	rs606149
chr1	150324283	150324284	rs11587682	chr1	157130565	157130566	rs6427356
chr1	114996323	114996324	rs11589568	chr1	114948280	114948281	rs6537825
chr1	98552831	98552832	rs1198588	chr1	110119731	110119732	rs6537837
chr1	70355980	70355981	rs12037173	chr1	212392162	212392163	rs6540731
chr1	110031187	110031188	rs12049330	chr1	115039118	115039119	rs6661053
chr1	239970096	239970097	rs12059546	chr1	222923350	222923351	rs6683071
chr1	97113914	97113915	rs12071951	chr1	30437267	30437268	rs6694545
chr1	38247152	38247153	rs12117544	chr1	243608966	243608967	rs6703335
chr1	107463642	107463643	rs12125971	chr1	230674825	230674826	rs701157
chr1	177722906	177722907	rs12140439	chr1	114999276	114999277	rs7511633
chr1	204572070	204572071	rs12143943	chr1	196042661	196042662	rs7512769
chr1	227667789	227667790	rs12410462	chr1	210536024	210536025	rs7527939
chr1	220658233	220658234	rs12563333	chr1	34181829	34181830	rs7533254
chr1	61831937	61831938	rs12568010	chr1	80134122	80134123	rs7533906
chr1	186870071	186870072	rs12720541	chr1	97164342	97164343	rs7544736
chr1	79254761	79254762	rs12730292	chr1	112856569	112856570	rs7555668
chr1	145645605	145645606	rs12744221	chr1	61821818	61821819	rs7556462
chr1	93401836	93401837	rs12745968	chr1	34750935	34750936	rs771390
chr1	243663892	243663893	rs14403	chr1	115259598	115259599	rs8453
chr1	43688549	43688550	rs150404479	chr1	5354843	5354844	rs912988
chr1	243544826	243544827	rs1538774	chr1	115239815	115239816	rs926938
chr1	98502933	98502934	rs1625579	chr1	16393356	16393357	rs9442235
chr1	160421915	160421916	rs16832015	chr2	206060636	206060637	rs10153620
chr1	34180841	34180842	rs16835742	chr2	193742172	193742173	rs10174573
chr1	61820071	61820072	rs17121983	chr2	146071852	146071853	rs10180522
chr1	115099091	115099092	rs1877455	chr2	104587350	104587351	rs10193430

chr1	147226790	147226791	rs1891498
chr1	101938476	101938477	rs1948368
chr1	88802011	88802012	rs2179965
chr1	30627711	30627712	rs2180233
chr1	8422675	8422676	rs2252865
chr1	34359857	34359858	rs2281597
chr1	200826768	200826769	rs2292096
chr1	200960306	200960307	rs2297909
chr1	217200882	217200883	rs2377360
chr1	234662889	234662890	rs271738
chr1	114715583	114715584	rs2774292
chr2	51667738	51667739	rs1206397
chr2	104580502	104580503	rs12613775
chr2	99083891	99083892	rs12617721
chr2	99239930	99239931	rs12618769
chr2	145141540	145141541	rs12991836
chr2	124515040	124515041	rs13006237
chr2	236795342	236795343	rs13025591
chr2	57934054	57934055	rs13026414
chr2	44135313	44135314	rs13387221
chr2	146092755	146092756	rs13407123
chr2	79539987	79539988	rs13409348
chr2	181076748	181076749	rs13418455
chr2	185778427	185778428	rs1344706
chr2	116241185	116241186	rs1375144
chr2	226237909	226237910	rs1517484
chr2	202949850	202949851	rs1521882
chr2	127330953	127330954	rs1550404
chr2	22576099	22576100	rs1605834
chr2	232318753	232318754	rs16828074
chr2	231420400	231420401	rs17275498
chr2	123641610	123641611	rs17367118
chr2	193984620	193984621	rs17662626
chr2	146044051	146044052	rs1822881
chr2	19134747	19134748	rs1876040
chr2	91697807	91697808	rs191753171
chr2	156888499	156888500	rs1918172
chr2	207121781	207121782	rs2058710
chr2	104573004	104573005	rs2119507
chr2	194872182	194872183	rs2176528
chr2	1925992	1925993	rs2241685
chr2	208994044	208994045	rs2242073
chr2	97405439	97405440	rs2271893
chr2	102663627	102663628	rs2310173
chr2	58222927	58222928	rs2312147
chr2	97413487	97413488	rs2314398
chr2	201582192	201582193	rs2348114
chr2	37592627	37592628	rs2373000
chr2	201571142	201571143	rs2540051
chr2	60762501	60762502	rs2556378
chr2	104578532	104578533	rs1036736
chr2	134005568	134005569	rs10496702
chr2	145359908	145359909	rs10496964
chr2	213609251	213609252	rs10497985
chr2	12568995	12568996	rs10929808
chr2	241522266	241522267	rs1133353
chr2	206063297	206063298	rs11681930
chr2	146045564	146045565	rs11687420
chr2	124983055	124983056	rs1170612
chr2	166943276	166943277	rs11890028
chr2	104589137	104589138	rs11903187
chr2	11220561	11220562	rs6739054
chr2	97410948	97410949	rs6746896
chr2	104667737	104667738	rs6758152
chr2	67942479	67942480	rs724568
chr2	201576283	201576284	rs7563911
chr2	20261684	20261685	rs7565124
chr2	48649704	48649705	rs7565792
chr2	104983266	104983267	rs7570682
chr2	134040518	134040519	rs7577925
chr2	99382891	99382892	rs7578035
chr2	55418934	55418935	rs7588591
chr2	201580953	201580954	rs7589014
chr2	78075243	78075244	rs7595103
chr2	185533579	185533580	rs7597593
chr2	233743108	233743109	rs778371
chr2	29280760	29280761	rs882632
chr2	224023295	224023296	rs895767
chr2	42981238	42981239	rs930421
chr2	137338939	137338940	rs934299
chr3	185686740	185686741	rs10049246
chr3	52821010	52821011	rs1042779
chr3	60289841	60289842	rs10510837
chr3	182121572	182121573	rs10513788
chr3	61413813	61413814	rs10514718
chr3	52718279	52718280	rs10865974
chr3	62064768	62064769	rs11130874
chr3	20108545	20108546	rs1124376
chr3	5705051	5705052	rs11710433
chr3	2895683	2895684	rs11713158
chr3	21955197	21955198	rs11719664
chr3	21248641	21248642	rs11720452
chr3	54632546	54632547	rs13064588
chr3	36842622	36842623	rs13072940
chr3	174029044	174029045	rs13074924
chr3	151763700	151763701	rs1351267
chr3	20615272	20615273	rs1395103
chr3	140676799	140676800	rs141220432
chr3	140681912	140681913	rs150888153
chr3	116761659	116761660	rs1521418

chr2	120321816	120321817	rs2587695	chr3	3658839	3658840	rs1601875
chr2	234584323	234584324	rs2602381	chr3	191196591	191196592	rs16822582
chr2	233736243	233736244	rs2675968	chr3	140979410	140979411	rs16851254
chr2	66258700	66258701	rs2699783	chr3	43230722	43230723	rs17075286
chr2	58094872	58094873	rs2717068	chr3	119084330	119084331	rs17203055
chr2	116655794	116655795	rs272000	chr3	2886526	2886527	rs17584516
chr2	200715387	200715388	rs2949006	chr3	71020489	71020490	rs17651978
chr2	241515595	241515596	rs2953145	chr3	55571759	55571760	rs1795648
chr2	240112971	240112972	rs3791556	chr3	180551213	180551214	rs1879248
chr2	65758524	65758525	rs3845817	chr3	183876511	183876512	rs1969253
chr2	12037491	12037492	rs4027132	chr3	178341581	178341582	rs2054399
chr2	185811939	185811940	rs4380187	chr3	122329159	122329160	rs2173763
chr2	48047334	48047335	rs4381823	chr3	52855228	52855229	rs2239547
chr2	315214	315215	rs4643574	chr3	52833218	52833219	rs2535629
chr2	171822465	171822466	rs4668356	chr3	52815904	52815905	rs2710323
chr2	206086170	206086171	rs4675502	chr3	1897972	1897973	rs2727943
chr2	176239848	176239849	rs4972755	chr3	121344139	121344140	rs3772130
chr2	149865249	149865250	rs6435387	chr3	21706368	21706369	rs3821396
chr2	99465501	99465502	rs6733011	chr3	194758009	194758010	rs3892715
chr2	34480074	34480075	rs6733379	chr3	32329496	32329497	rs4380451
chr3	111423128	111423129	rs4450776	chr4	55482817	55482818	rs2537859
chr3	13736999	13737000	rs4450798	chr4	183137397	183137398	rs2726807
chr3	191818641	191818642	rs4585146	chr4	152908168	152908169	rs360932
chr3	36862979	36862980	rs4624519	chr4	188191305	188191306	rs4478239
chr3	32347823	32347824	rs4627791	chr4	41284710	41284711	rs4861096
chr3	2893273	2893274	rs4629318	chr4	125415533	125415534	rs6534441
chr3	52838401	52838402	rs4687552	chr4	160282717	160282718	rs6536413
chr3	182870435	182870436	rs514636	chr4	180687999	180688000	rs6844851
chr3	34865596	34865597	rs559356	chr4	176008217	176008218	rs72698613
chr3	193583709	193583710	rs604222	chr4	29903051	29903052	rs7442317
chr3	170163473	170163474	rs6444931	chr4	83499312	83499313	rs7658637
chr3	177291696	177291697	rs644695	chr4	189587078	189587079	rs7659062
chr3	36864488	36864489	rs6550435	chr4	189590858	189590859	rs7662358
chr3	52733105	52733106	rs6765687	chr4	145196205	145196206	rs7690204
chr3	2881574	2881575	rs6781149	chr4	102589282	102589283	rs7700191
chr3	180550701	180550702	rs6782299	chr4	139809180	139809181	rs77803164
chr3	60771107	60771108	rs6791644	chr4	2395296	2395297	rs959770
chr3	35982129	35982130	rs6799705	chr4	89220943	89220944	rs9995093
chr3	159644296	159644297	rs6799788	chr5	25902341	25902342	rs10038113
chr3	182869817	182869818	rs683395	chr5	178570913	178570914	rs10039254
chr3	140370011	140370012	rs72977016	chr5	51217850	51217851	rs10042348
chr3	52835353	52835354	rs736408	chr5	56666499	56666500	rs10052004
chr3	184945848	184945849	rs7374394	chr5	159007777	159007778	rs10065906
chr3	163761963	163761964	rs7427021	chr5	107281620	107281621	rs10070308
chr3	175223895	175223896	rs74619861	chr5	101726769	101726770	rs10073892
chr3	177596988	177596989	rs7612209	chr5	123192433	123192434	rs1021769
chr3	52279593	52279594	rs7618915	chr5	127110485	127110486	rs10463832
chr3	184876782	184876783	rs7647854	chr5	127122311	127122312	rs10463833
chr3	140417445	140417446	rs79436609	chr5	9623621	9623622	rs10513025
chr3	39553787	39553788	rs816488	chr5	6834166	6834167	rs11134178

chr3	39555579	39555580	rs864643
chr3	14530720	14530721	rs9036
chr3	142982898	142982899	rs9810857
chr3	36856029	36856030	rs9834970
chr3	32842100	32842101	rs9845475
chr3	7529554	7529555	rs9870680
chr3	25112170	25112171	rs993804
chr4	111026926	111026927	rs10011926
chr4	150725211	150725212	rs10030601
chr4	59008411	59008412	rs10517437
chr4	39286948	39286949	rs11096990
chr4	95146134	95146135	rs11097407
chr4	118646906	118646907	rs11098403
chr4	131131415	131131416	rs11099040
chr4	67733856	67733857	rs1155865
chr4	189861250	189861251	rs11731175
chr4	166147711	166147712	rs12500138
chr4	24740957	24740958	rs12500612
chr4	126426694	126426695	rs12639834
chr4	131132899	131132900	rs13113376
chr4	75224589	75224590	rs1350666
chr4	126359455	126359456	rs1395241
chr4	180655624	180655625	rs17746001
chr4	82462596	82462597	rs1822818
chr4	95834033	95834034	rs1859156
chr4	131133490	131133491	rs1908038
chr4	131127632	131127633	rs1908039
chr4	125265312	125265313	rs2125000
chr4	103457417	103457418	rs230529
chr5	115568756	115568757	rs253959
chr5	38051592	38051593	rs270545
chr5	152540353	152540354	rs2910032
chr5	17215443	17215444	rs2962370
chr5	82889909	82889910	rs310501
chr5	143411345	143411346	rs391760
chr5	66152257	66152258	rs39861
chr5	143422944	143422945	rs409220
chr5	152288452	152288453	rs4262150
chr5	25967702	25967703	rs4307059
chr5	112242967	112242968	rs469727
chr5	97888735	97888736	rs4703129
chr5	127054277	127054278	rs4835929
chr5	3939475	3939476	rs492478
chr5	156945147	156945148	rs58873874
chr5	146225243	146225244	rs609412
chr5	3276695	3276696	rs61670327
chr5	78110531	78110532	rs6453417
chr5	89051856	89051857	rs6864869
chr5	130413980	130413981	rs6867265
chr5	101769725	101769726	rs6878284

chr5	156942284	156942285	rs11740562
chr5	40626752	40626753	rs11955175
chr5	31917241	31917242	rs11957407
chr5	27611430	27611431	rs12054895
chr5	123832651	123832652	rs12513663
chr5	127059941	127059942	rs12513840
chr5	127061970	127061971	rs12523164
chr5	125338474	125338475	rs13169113
chr5	64534640	64534641	rs13183791
chr5	92343194	92343195	rs147721431
chr5	101859316	101859317	rs1502844
chr5	127139615	127139616	rs1515641
chr5	133203595	133203596	rs1644305
chr5	141210752	141210753	rs166040
chr5	87034351	87034352	rs1673101
chr5	60499130	60499131	rs171748
chr5	10811473	10811474	rs17176973
chr5	64433399	64433400	rs17206232
chr5	112580289	112580290	rs17323670
chr5	111107114	111107115	rs173896
chr5	94154587	94154588	rs17418283
chr5	152654478	152654479	rs17504622
chr5	2335580	2335581	rs17586674
chr5	16812637	16812638	rs17651119
chr5	149036975	149036976	rs17710780
chr5	7519297	7519298	rs17826816
chr5	71410355	71410356	rs2199161
chr5	106724587	106724588	rs252817
chr5	11298222	11298223	rs2530215
chr6	93615449	93615450	rs2506933
chr6	30165272	30165273	rs2523722
chr6	29899676	29899677	rs2524005
chr6	100756686	100756687	rs2841307
chr6	41650735	41650736	rs2842643
chr6	32358269	32358270	rs3117099
chr6	32172992	32172993	rs3131296
chr6	30913457	30913458	rs3132581
chr6	32171074	32171075	rs3132935
chr6	30080368	30080369	rs34704616
chr6	44837355	44837356	rs3799977
chr6	9030335	9030336	rs438259
chr6	32581888	32581889	rs4530903
chr6	168484084	168484085	rs4708431
chr6	164900597	164900598	rs4709845
chr6	39539206	39539207	rs4714261
chr6	52835894	52835895	rs492146
chr6	87466491	87466492	rs493187
chr6	113160343	113160344	rs62421103
chr6	42731114	42731115	rs6458307
chr6	14596364	14596365	rs6914079

chr5	107282347	107282348	rs72795979
chr5	107282348	107282349	rs72795980
chr5	175244033	175244034	rs7448069
chr5	60731457	60731458	rs7709645
chr5	162083517	162083518	rs7711337
chr5	78829248	78829249	rs7713917
chr5	127125761	127125762	rs7722425
chr5	4720584	4720585	rs7727102
chr5	7228046	7228047	rs7729273
chr5	79099463	79099464	rs7735699
chr5	156970143	156970144	rs77372450
chr5	146415215	146415216	rs9325032
chr6	166155456	166155457	rs1039002
chr6	72482457	72482458	rs10455248
chr6	31602966	31602967	rs1046089
chr6	16256042	16256043	rs10484358
chr6	113134475	113134476	rs10782174
chr6	136545272	136545273	rs113508841
chr6	84226594	84226595	rs1171113
chr6	89732100	89732101	rs12201676
chr6	98576222	98576223	rs12202969
chr6	475488	475489	rs12210050
chr6	10176035	10176036	rs12210761
chr6	27143882	27143883	rs13194053
chr6	121339178	121339179	rs1343075
chr6	32913245	32913246	rs1480380
chr6	28227603	28227604	rs1635
chr6	37174744	37174745	rs1680005
chr6	33851172	33851173	rs16869652
chr6	27278019	27278020	rs16897515
chr6	130019353	130019354	rs17057640
chr6	108323758	108323759	rs17069122
chr6	152738753	152738754	rs17082664
chr6	37487043	37487044	rs1757171
chr6	27710164	27710165	rs17693963
chr6	30174130	30174131	rs2021722
chr6	90640490	90640491	rs2289577
chr6	6612466	6612467	rs2326810
chr7	71751315	71751316	rs12699131
chr7	94006993	94006994	rs13221576
chr7	96362343	96362344	rs1464807
chr7	70473004	70473005	rs1525293
chr7	31155346	31155347	rs1558477
chr7	21504426	21504427	rs17144465
chr7	12937254	12937255	rs17166499
chr7	153535688	153535689	rs2110267
chr7	153549295	153549296	rs2192271
chr7	28762962	28762963	rs2237349
chr7	29041189	29041190	rs2252521
chr7	22985009	22985010	rs2286492

chr6	124342662	124342663	rs6917824
chr6	50352135	50352136	rs6921059
chr6	27248930	27248931	rs6932590
chr6	113124300	113124301	rs6934970
chr6	44065310	44065311	rs7742824
chr6	152791473	152791474	rs7747960
chr6	91207350	91207351	rs806276
chr6	30032521	30032522	rs8321
chr6	30782001	30782002	rs886424
chr6	37451695	37451696	rs904251
chr6	32431961	32431962	rs9268895
chr6	32602268	32602269	rs9272219
chr6	32675108	32675109	rs9275524
chr6	168490567	168490568	rs9364220
chr6	152790572	152790573	rs9371601
chr6	104593866	104593867	rs9377619
chr6	31327700	31327701	rs9378249
chr6	23841131	23841132	rs9466930
chr7	35610161	35610162	rs1003247
chr7	112628372	112628373	rs10229603
chr7	70045940	70045941	rs10237317
chr7	136146535	136146536	rs10250997
chr7	137858765	137858766	rs10255295
chr7	94000472	94000473	rs10262915
chr7	130900120	130900121	rs10265216
chr7	1920825	1920826	rs10275045
chr7	110467220	110467221	rs10279573
chr7	7399403	7399404	rs10486158
chr7	144841530	144841531	rs10487524
chr7	156048648	156048649	rs10949808
chr7	131815342	131815343	rs10954361
chr7	2041431	2041432	rs1107592
chr7	105222450	105222451	rs116979167
chr7	86821979	86821980	rs11773103
chr7	125896747	125896748	rs117982730
chr7	38835034	38835035	rs11984145
chr7	2004420	2004421	rs12666575
chr7	153558948	153558949	rs12671878
chr8	11276541	11276542	rs2002030
chr8	138194868	138194869	rs2077233
chr8	125697672	125697673	rs2116081
chr8	2040103	2040104	rs2235121
chr8	73633027	73633028	rs2247572
chr8	144657599	144657600	rs2290416
chr8	15665611	15665612	rs240657
chr8	34236991	34236992	rs2609653
chr8	4483140	4483141	rs2616984
chr8	116633698	116633699	rs2721937
chr8	94169349	94169350	rs278567
chr8	134668676	134668677	rs2860223

chr7	81786219	81786220	rs2367911	chr8	13260535	13260536	rs289585
chr7	21861111	21861112	rs2390593	chr8	30498858	30498859	rs2978263
chr7	2298226	2298227	rs2398668	chr8	107844446	107844447	rs2981205
chr7	45982459	45982460	rs2462686	chr8	143312932	143312933	rs4129585
chr7	20862301	20862302	rs2709736	chr8	90342185	90342186	rs4397449
chr7	82450034	82450035	rs2715148	chr8	36941541	36941542	rs4739466
chr7	24692551	24692552	rs2721800	chr8	5461752	5461753	rs4875598
chr7	1950808	1950809	rs4332037	chr8	102944862	102944863	rs517811
chr7	83772414	83772415	rs447	chr8	4238138	4238139	rs6558872
chr7	2036668	2036669	rs4721295	chr8	104118565	104118566	rs6983777
chr7	94008534	94008535	rs4729127	chr8	121635395	121635396	rs6986718
chr7	110047963	110047964	rs4730430	chr8	34126947	34126948	rs6990255
chr7	57699150	57699151	rs4870684	chr8	89760310	89760311	rs7004633
chr7	157510194	157510195	rs6459804	chr8	10022937	10022938	rs7017212
chr7	2017444	2017445	rs6461049	chr8	142359549	142359550	rs7386474
chr7	94003259	94003260	rs6465411	chr8	23101619	23101620	rs7463256
chr7	153553063	153553064	rs6947495	chr8	143691837	143691838	rs7465272
chr7	1886534	1886535	rs6952808	chr8	142300314	142300315	rs7827290
chr7	105166852	105166853	rs6963495	chr8	72561741	72561742	rs7834018
chr7	110047470	110047471	rs6968385	chr8	106142332	106142333	rs9297357
chr7	137529577	137529578	rs6978230	chr8	101919320	101919321	rs931812
chr7	103404814	103404815	rs7341475	chr8	59708308	59708309	rs960089
chr7	116577123	116577124	rs7782376	chr8	138905295	138905296	rs9657451
chr7	8142926	8142927	rs7791362	chr9	17895104	17895105	rs10810865
chr7	94009633	94009634	rs7792596	chr9	6784725	6784726	rs10815468
chr7	151546588	151546589	rs7795096	chr9	8235632	8235633	rs10815798
chr7	2278225	2278226	rs7799006	chr9	138275775	138275776	rs10858396
chr7	26606004	26606005	rs7800418	chr9	117260833	117260834	rs10982256
chr7	83609038	83609039	rs797820	chr9	119333682	119333683	rs10983238
chr7	145959242	145959243	rs802568	chr9	137285502	137285503	rs11102986
chr8	94085538	94085539	rs1027730	chr9	135618282	135618283	rs11243897
chr8	124831833	124831834	rs10481151	chr9	121359285	121359286	rs11789399
chr8	4180843	4180844	rs10503253	chr9	15283096	15283097	rs12003180
chr8	4214178	4214179	rs10503256	chr9	135632492	135632493	rs12552369
chr8	20066048	20066049	rs1106634	chr9	12582564	12582565	rs1325154
chr8	139504269	139504270	rs11166827	chr9	36998992	36998993	rs1329573
chr8	93219720	93219721	rs116951791	chr9	90159312	90159313	rs1421001
chr8	54555199	54555200	rs11773966	chr9	108427061	108427062	rs1463984
chr8	49053164	49053165	rs11778329	chr9	121346416	121346417	rs1572299
chr8	73070734	73070735	rs11994034	chr9	90031668	90031669	rs1806864
chr8	89592082	89592083	rs11995572	chr9	93409356	93409357	rs1831521
chr8	70809260	70809261	rs12680109	chr9	17899605	17899606	rs1977882
chr8	27076597	27076598	rs1446682	chr9	33799369	33799370	rs216345
chr8	9986654	9986655	rs1484642	chr9	15309927	15309928	rs2245641
chr8	38031344	38031345	rs16887244	chr9	130976556	130976557	rs2502731
chr8	116394074	116394075	rs17658378	chr9	126926106	126926107	rs2807580
chr8	58840923	58840924	rs1992045	chr9	135845034	135845035	rs2905072
chr9	137269455	137269456	rs34312136	chr10	72982984	72982985	rs16928529
chr9	137280938	137280939	rs35079168	chr10	72462993	72462994	rs17600642
chr9	955793	955794	rs364477	chr10	18734527	18734528	rs17691888

chr9	71943001	71943002	rs3750552	chr10	32309004	32309005	rs1775715
chr9	36997416	36997417	rs3758171	chr10	34257611	34257612	rs1780436
chr9	75764564	75764565	rs3758354	chr10	119181870	119181871	rs181500
chr9	37000686	37000687	rs3824344	chr10	5348336	5348337	rs1987511
chr9	130107963	130107964	rs4130590	chr10	24634955	24634956	rs2484873
chr9	19664080	19664081	rs4258076	chr10	3284006	3284007	rs2764980
chr9	137284922	137284923	rs4501664	chr10	18601927	18601928	rs2799573
chr9	2194226	2194227	rs4741652	chr10	114874018	114874019	rs290475
chr9	78028345	78028346	rs489332	chr10	21567564	21567565	rs3847375
chr9	112521125	112521126	rs4978848	chr10	116139028	116139029	rs4751674
chr9	111799562	111799563	rs643410	chr10	62185493	62185494	rs4948418
chr9	37002114	37002115	rs7020413	chr10	105966403	105966404	rs515910
chr9	113197262	113197263	rs7042161	chr10	6946262	6946263	rs6602217
chr9	26935995	26935996	rs7045881	chr10	80955066	80955067	rs703965
chr9	10505223	10505224	rs72700966	chr10	80953135	80953136	rs703970
chr9	124098534	124098535	rs767770	chr10	105297769	105297770	rs7069733
chr9	22819575	22819576	rs7849973	chr10	104628872	104628873	rs7085104
chr9	94822539	94822540	rs7872515	chr10	5349870	5349871	rs7475343
chr9	33217265	33217266	rs830407	chr10	5354676	5354677	rs7896729
chr9	82310897	82310898	rs914715	chr10	20792333	20792334	rs7897194
chrX	151655561	151655562	rs10856240	chr10	104662457	104662458	rs7897654
chrX	153006494	153006495	rs11156606	chr10	68598291	68598292	rs7902091
chrX	82535168	82535169	rs1410530	chr10	104775907	104775908	rs7914558
chrX	32225254	32225255	rs1456737	chr10	5353689	5353690	rs9423406
chrX	82711941	82711942	rs213443	chr10	130248925	130248926	rs9804317
chrX	147381020	147381021	rs2159767	chr11	24741756	24741757	rs1021261
chrX	153207544	153207545	rs2269372	chr11	134088681	134088682	rs1031381
chrX	990179	990180	rs4129148	chr11	43086093	43086094	rs10501293
chrX	148567450	148567451	rs530501	chr11	63991800	63991801	rs1059440
chrX	139166682	139166683	rs5907577	chr11	32522006	32522007	rs10767942
chrX	11048691	11048692	rs5934953	chr11	94667963	94667964	rs10831284
chrX	89288226	89288227	rs5941436	chr11	24739803	24739804	rs10834449
chrX	139314055	139314056	rs5955415	chr11	130830747	130830748	rs10894294
chrX	95079869	95079870	rs5990417	chr11	66551001	66551002	rs10896135
chrX	144141318	144141319	rs6627057	chr11	33868606	33868607	rs11032423
chrX	53974053	53974054	rs7065696	chr11	44843133	44843134	rs11038167
chrX	8394252	8394253	rs7892812	chr11	125323964	125323965	rs11220082
chr10	18388433	18388434	rs10508558	chr11	79962148	79962149	rs11231991
chr10	62085336	62085337	rs10761482	chr11	80562737	80562738	rs11232369
chr10	98135504	98135505	rs10786284	chr11	63963946	63963947	rs11607165
chr10	44827196	44827197	rs10900020	chr11	22312565	22312566	rs11827962
chr10	62179811	62179812	rs10994336	chr11	18265798	18265799	rs12282742
chr10	62181127	62181128	rs10994338	chr11	79083619	79083620	rs12290811
chr10	62222106	62222107	rs10994359	chr11	63979642	63979643	rs12575642
chr10	62279123	62279124	rs10994397	chr11	79077192	79077193	rs12576775
chr10	62322033	62322034	rs10994415	chr11	120809459	120809460	rs12797755
chr10	64223382	64223383	rs10995170	chr11	24741658	24741659	rs12798374
chr10	104660003	104660004	rs11191454	chr11	124606284	124606285	rs12807809
chr10	104906210	104906211	rs11191580	chr11	131320069	131320070	rs1550976
chr10	45123378	45123379	rs11239177	chr11	126619189	126619190	rs1557488

chr10	6407736	6407737	rs11258317	chr11	28009462	28009463	rs1568889
chr10	114880550	114880551	rs12772424	chr11	29162135	29162136	rs1602565
chr10	34088052	34088053	rs1412115	chr11	10740365	10740366	rs2018368
chr10	29291897	29291898	rs1612122	chr11	107756550	107756551	rs2186903
chr10	73422283	73422284	rs1625975	chr11	94312322	94312323	rs2212361
chr10	62346637	62346638	rs16915157	chr11	66335307	66335308	rs2242663
chr11	98125403	98125404	rs2509843	chr12	70331826	70331827	rs789560
chr11	24740433	24740434	rs2716458	chr12	33701165	33701166	rs9300212
chr11	76823483	76823484	rs3781684	chr12	65252117	65252118	rs939876
chr11	120520628	120520629	rs4245040	chr12	69370755	69370756	rs9943849
chr11	17160147	17160148	rs4356203	chr13	42653436	42653437	rs1012053
chr11	13331225	13331226	rs4757144	chr13	108816224	108816225	rs10492664
chr11	88741659	88741660	rs5016282	chr13	81357670	81357671	rs11149178
chr11	79065100	79065101	rs530965	chr13	29431337	29431338	rs1161463
chr11	124537994	124537995	rs544368	chr13	51066622	51066623	rs1262778
chr11	125461708	125461709	rs548181	chr13	108668546	108668547	rs12871532
chr11	10390580	10390581	rs6484218	chr13	43727848	43727849	rs1324015
chr11	86055900	86055901	rs6592284	chr13	97508738	97508739	rs16953622
chr11	125313598	125313599	rs7930295	chr13	79359423	79359424	rs17070284
chr11	130817578	130817579	rs7940866	chr13	85665878	85665879	rs17079247
chr11	116400381	116400382	rs7941534	chr13	28929710	28929711	rs17086609
chr11	110243921	110243922	rs7945071	chr13	43834269	43834270	rs17538444
chr11	118341648	118341649	rs7948661	chr13	101708309	101708310	rs2044117
chr11	19569562	19569563	rs874426	chr13	61965302	61965303	rs2323266
chr11	131336073	131336074	rs992564	chr13	72768708	72768709	rs4083578
chr12	2345294	2345295	rs1006737	chr13	23692977	23692978	rs4770394
chr12	126143345	126143346	rs1043607	chr13	26037959	26037960	rs4770837
chr12	99498186	99498187	rs10860392	chr13	106651660	106651661	rs4996815
chr12	73307581	73307582	rs10879517	chr13	54435236	54435237	rs6561750
chr12	13519995	13519996	rs11055387	chr13	111030577	111030578	rs7319311
chr12	119818508	119818509	rs11064768	chr13	21209511	21209512	rs7326068
chr12	120455763	120455764	rs11064994	chr13	111470037	111470038	rs767210
chr12	48403764	48403765	rs11168351	chr13	70455660	70455661	rs7984606
chr12	68956184	68956185	rs114588203	chr13	100555037	100555038	rs7992643
chr12	123731422	123731423	rs11532322	chr13	94408505	94408506	rs7995215
chr12	68957163	68957164	rs116075389	chr13	40948150	40948151	rs913246
chr12	125546574	125546575	rs12300899	chr13	28077143	28077144	rs9512730
chr12	68532076	68532077	rs12321565	chr13	28429737	28429738	rs9512900
chr12	125033932	125033933	rs12423712	chr13	94341095	94341096	rs9561428
chr12	81843127	81843128	rs12426725	chr13	70846202	70846203	rs9572423
chr12	13641705	13641706	rs1457614	chr13	80169003	80169004	rs9601248
chr12	68955571	68955572	rs150516896	chr13	88762345	88762346	rs969962
chr12	84564067	84564068	rs1545843	chr14	58119195	58119196	rs10134944
chr12	5131552	5131553	rs16932667	chr14	64899054	64899055	rs10498514
chr12	29872317	29872318	rs16934812	chr14	58200724	58200725	rs1092015
chr12	108742819	108742820	rs17040430	chr14	104509075	104509076	rs11622475
chr12	17662234	17662235	rs1706631	chr14	24381514	24381515	rs12436436
chr12	107704704	107704705	rs1820460	chr14	50877982	50877983	rs1265879
chr12	114705585	114705586	rs1920592	chr14	58385364	58385365	rs1335515
chr12	3913426	3913427	rs2058350	chr14	62678302	62678303	rs1514928

chr12	49218170	49218171	rs2070615	chr14	27406891	27406892	rs17111920
chr12	13787845	13787846	rs2160519	chr14	51084234	51084235	rs17122693
chr12	15735441	15735442	rs2300290	chr14	21725653	21725654	rs17197037
chr12	41247741	41247742	rs312273	chr14	97171074	97171075	rs17244419
chr12	133417801	133417802	rs3741489	chr14	50973631	50973632	rs17718580
chr12	106950694	106950695	rs3891355	chr14	27260042	27260043	rs1951082
chr12	4118316	4118317	rs4238010	chr14	77684841	77684842	rs2287375
chr12	4416303	4416304	rs4625554	chr14	73767213	73767214	rs2333194
chr12	2349583	2349584	rs4765905	chr14	49372800	49372801	rs2352904
chr12	2419895	2419896	rs4765913	chr14	76812490	76812491	rs2360997
chr12	104192823	104192824	rs4964805	chr14	77684669	77684670	rs4467006
chr12	28228566	28228567	rs522958	chr14	33476265	33476266	rs4982029
chr12	19801594	19801595	rs6486986	chr14	78786158	78786159	rs6574433
chr12	97434219	97434220	rs6538761	chr14	88559991	88559992	rs6574988
chr12	80062229	80062230	rs7297018	chr14	102360744	102360745	rs7142002
chr14	48564955	48564956	rs7151223	chr16	89741495	89741496	rs12443954
chr14	63322346	63322347	rs8012941	chr16	7753703	7753704	rs12444931
chr14	33480921	33480922	rs8015959	chr16	73501535	73501536	rs12446956
chr14	51170878	51170879	rs8020441	chr16	20056992	20056993	rs12596252
chr14	32433857	32433858	rs915071	chr16	20126973	20126974	rs12919130
chr15	74012436	74012437	rs1038094	chr16	6910384	6910385	rs12921846
chr15	36313964	36313965	rs10520045	chr16	12077631	12077632	rs12922317
chr15	88723711	88723712	rs1104918	chr16	20094545	20094546	rs12924103
chr15	69650052	69650053	rs11072089	chr16	20059676	20059677	rs12926725
chr15	51352248	51352249	rs1124769	chr16	20100369	20100370	rs12926729
chr15	70369377	70369378	rs11630316	chr16	20060829	20060830	rs12931939
chr15	68892988	68892989	rs11856323	chr16	52912298	52912299	rs1344484
chr15	61870942	61870943	rs12592967	chr16	63955446	63955447	rs1381102
chr15	38995490	38995491	rs12899449	chr16	20674491	20674492	rs151222
chr15	38986367	38986368	rs12912251	chr16	71965195	71965196	rs16973500
chr15	30898331	30898332	rs143536437	chr16	57024316	57024317	rs17290922
chr15	38511982	38511983	rs16966460	chr16	20057660	20057661	rs1902813
chr15	86984239	86984240	rs16977195	chr16	27056454	27056455	rs2203512
chr15	93889458	93889459	rs17541406	chr16	23640466	23640467	rs249954
chr15	23647748	23647749	rs183418565	chr16	20122289	20122290	rs2608200
chr15	40595626	40595627	rs1869901	chr16	87678575	87678576	rs34975147
chr15	54341464	54341465	rs1897031	chr16	83757327	83757328	rs3784962
chr15	80694921	80694922	rs2278702	chr16	82379952	82379953	rs4082514
chr15	79224682	79224683	rs2289700	chr16	84213683	84213684	rs4150167
chr15	39003762	39003763	rs2643217	chr16	23634025	23634026	rs420259
chr15	91450440	91450441	rs2677744	chr16	20680205	20680206	rs433598
chr15	59719168	59719169	rs28890483	chr16	77916190	77916191	rs435746
chr15	57762792	57762793	rs2934442	chr16	81156521	81156522	rs4889240
chr15	62312839	62312840	rs4143844	chr16	20123514	20123515	rs6497436
chr15	68198910	68198911	rs448720	chr16	20094885	20094886	rs7185264
chr15	97262426	97262427	rs4533251	chr16	13061610	13061611	rs7192086
chr15	58635582	58635583	rs4775031	chr16	20094053	20094054	rs7201408
chr15	61840102	61840103	rs4775413	chr16	69135048	69135049	rs8047014
chr15	36293604	36293605	rs4923705	chr16	86104141	86104142	rs8050326
chr15	70480613	70480614	rs6494849	chr16	82692811	82692812	rs8057927

chr15	95511836	95511837	rs6496074
chr15	100299238	100299239	rs6598266
chr15	68715031	68715032	rs7164335
chr15	24062914	24062915	rs7164923
chr15	61336441	61336442	rs7172342
chr15	68597126	68597127	rs7174755
chr15	94036687	94036688	rs7175404
chr15	59174538	59174539	rs7179456
chr15	53279377	53279378	rs719714
chr15	49192790	49192791	rs8023445
chr15	79552378	79552379	rs8025118
chr15	93044338	93044339	rs8040009
chr15	26973085	26973086	rs8043440
chr15	33505066	33505067	rs974379
chr16	84446383	84446384	rs10514604
chr16	20089753	20089754	rs10521114
chr16	20090644	20090645	rs10521115
chr16	10633164	10633165	rs11074889
chr16	78466703	78466704	rs11150078
chr16	20109652	20109653	rs11642377
chr16	82746936	82746937	rs11646411
chr16	20095627	20095628	rs11647507
chr16	8517744	8517745	rs11861787
chr16	9675245	9675246	rs12325410
chr18	65483811	65483812	rs11661646
chr18	40872272	40872273	rs12457996
chr18	52949656	52949657	rs1261117
chr18	52752016	52752017	rs12966547
chr18	11494198	11494199	rs1455244
chr18	66314071	66314072	rs1704734
chr18	65285278	65285279	rs17077540
chr18	66292258	66292259	rs17232800
chr18	53194960	53194961	rs17512836
chr18	53058237	53058238	rs17594526
chr18	73192445	73192446	rs1865721
chr18	42090666	42090667	rs2048485
chr18	65017099	65017100	rs2124349
chr18	10923807	10923808	rs264272
chr18	26676671	26676672	rs4145170
chr18	75912921	75912922	rs4798896
chr18	52752699	52752700	rs4801131
chr18	278795	278796	rs7226677
chr18	77392378	77392379	rs7233060
chr18	55434201	55434202	rs7236632
chr18	65471517	65471518	rs74789784
chr18	65472042	65472043	rs75687134
chr18	40288292	40288293	rs8085804
chr18	52821123	52821124	rs9951150
chr18	53155001	53155002	rs9960767
chr19	19361734	19361735	rs1064395
chr16	9911385	9911386	rs8058295
chr16	57058581	57058582	rs821470
chr16	87461968	87461969	rs9646303
chr17	47564011	47564012	rs1035050
chr17	55866286	55866287	rs12938916
chr17	64917032	64917033	rs17645023
chr17	3664974	3664975	rs220470
chr17	41965199	41965200	rs231513
chr17	46720564	46720565	rs2326017
chr17	75211207	75211208	rs3744064
chr17	6093950	6093951	rs3744728
chr17	39250638	39250639	rs4006360
chr17	2208898	2208899	rs4523957
chr17	6781412	6781413	rs58630086
chr17	36026334	36026335	rs6607284
chr17	46840540	46840541	rs7219021
chr17	46123003	46123004	rs72823592
chr17	79024636	79024637	rs8067235
chr17	63642541	63642542	rs8074751
chr17	56847944	56847945	rs9303401
chr18	57314806	57314807	rs11152166
chr18	53066327	53066328	rs11152369
chr18	65470748	65470749	rs114535501
chr18	65470133	65470134	rs11660238
chr20	60106872	60106873	rs116896199
chr20	55683995	55683996	rs11699237
chr20	51778122	51778123	rs118174081
chr20	47088153	47088154	rs13043694
chr20	594779	594780	rs1533087
chr20	33849178	33849179	rs1555322
chr20	4352103	4352104	rs159788
chr20	31447367	31447368	rs17123726
chr20	52610495	52610496	rs2276498
chr20	24717084	24717085	rs2424635
chr20	3776174	3776175	rs3761218
chr20	14747470	14747471	rs4141463
chr20	47089524	47089525	rs4402823
chr20	47089536	47089537	rs4458264
chr20	46400712	46400713	rs4810685
chr20	47088199	47088200	rs4810796
chr20	19865955	19865956	rs4814920
chr20	42854133	42854134	rs6017291
chr20	36957836	36957837	rs6024905
chr20	13696128	13696129	rs6042314
chr20	16451641	16451642	rs6043979
chr20	16459308	16459309	rs6044001
chr20	16460714	16460715	rs6044003
chr20	19852502	19852503	rs6046396
chr20	20848967	20848968	rs6047116
chr20	25019098	25019099	rs6050267

chr19	31801129	31801130	rs1078373	chr20	8836770	8836771	rs6056209
chr19	45403215	45403216	rs115881343	chr20	31384879	31384880	rs6057648
chr19	6080481	6080482	rs11880706	chr20	31410336	31410337	rs6057651
chr19	40229408	40229409	rs12611334	chr20	31419808	31419809	rs6057652
chr19	56265663	56265664	rs17634917	chr20	31445146	31445147	rs6057659
chr19	12225371	12225372	rs17638629	chr20	8114703	8114704	rs6118083
chr19	33089430	33089431	rs17692896	chr20	31376994	31376995	rs6119285
chr19	11330941	11330942	rs17699030	chr20	31395476	31395477	rs6119286
chr19	3251218	3251219	rs17764205	chr20	49375241	49375242	rs6122972
chr19	19443985	19443986	rs2011503	chr20	43721492	43721493	rs6124684
chr19	45395618	45395619	rs2075650	chr20	31437606	31437607	rs7270085
chr19	32917454	32917455	rs2111504	chr20	31446857	31446858	rs8123073
chr19	49228271	49228272	rs2287921	chr20	47088731	47088732	rs910191
chr19	48525506	48525507	rs2303690	chr20	16455773	16455774	rs932541
chr19	34320853	34320854	rs2546057	chr21	27002376	27002377	rs17001239
chr19	48531215	48531216	rs2560966	chr21	16340288	16340289	rs2229741
chr19	58770882	58770883	rs260461	chr21	44445291	44445292	rs234720
chr19	19473444	19473445	rs2905424	chr21	11002010	11002011	rs240444
chr19	48522868	48522869	rs3815908	chr21	17828290	17828291	rs2823819
chr19	48527581	48527582	rs3936340	chr21	20536648	20536649	rs2825388
chr19	34321257	34321258	rs398426	chr21	21885301	21885302	rs2826340
chr19	45422945	45422946	rs4420638	chr21	25143118	25143119	rs2828520
chr19	42066278	42066279	rs4803480	chr21	30141020	30141021	rs2832077
chr19	52338640	52338641	rs62110082	chr21	31013158	31013159	rs363598
chr19	35973288	35973289	rs6510489	chr21	34734509	34734510	rs9980664
chr19	12691184	12691185	rs7247513	chr22	26403598	26403599	rs1001021
chr19	18467885	18467886	rs7248363	chr22	34823827	34823828	rs1034394
chr19	1811602	1811603	rs7250872	chr22	32024979	32024980	rs11703808
chr19	14713492	14713493	rs7254215	chr22	32027448	32027449	rs12627933
chr19	45410001	45410002	rs769449	chr22	34859792	34859793	rs130575
chr19	42521107	42521108	rs8099939	chr22	42670964	42670965	rs134882
chr19	38912763	38912764	rs892055	chr22	50218610	50218611	rs138880
chr20	51583982	51583983	rs10485813	chr22	27343453	27343454	rs16983214
chr22	40996366	40996367	rs17002034	chr22	48923458	48923459	rs80088139
chr22	25875264	25875265	rs1930961	chr22	39955872	39955873	rs9611198
chr22	22307518	22307519	rs412050	chr22	32049916	32049917	rs9621305
chr22	27011419	27011420	rs4822752	chr22	48284513	48284514	rs9627183
chr22	32229648	32229649	rs5994434				
chr22	32025912	32025913	rs761746				

Supplemental Table S3. RefSeq protein-coding genes presenting with neuron specific H3K4me3, REST binding or DNAse 1 hypersensitivity at their transcription start site. For each mark or combination of marks, the number of ID genes or genes harbouring a GWAS hit for CNS disorders, presenting with this mark is shown. Using Fisher's exact test it was determined whether these ID genes and GWAS hits were significantly enriched in the resulting gene sets (odds ratios (OR) between brackets). In the second half of the table, the values following REF (*) represent enrichment using the H3K4me3 sets as reference.

	Total	*ID genes*	*Fisher p-val (& OR)*	*+ GWAS hit*	*Fisher p-val (& OR)*
**RefSeq coding genes**	19233	1134	= REF	380	= REF
+ REST binding	12696	893	2.701e-05 (1.21)	253	0.4728 (1.01)
+ H3K4me3 (*)	7003	581	6.252e-12 (1.44)	221	2.794e-08 (1.62)
+ DNAseI hypersens.	15144	1024	0.0005646 (1.16)	311	0.3181 (1.04)
+ H3K4me3 & REST	5537	484	1.616e-13 (1.53)	157	0.0001037 (1.45)
			REF (*): 0.1962 (1.06)		REF (*): 0.8632 (0.90)
+ H3K4me3 & DHS	6568	548	7.86e-12 (1.45)	191	9.887e-06 (1.49)
			REF (*): 0.4728 (1.00)		REF(*): 0.8134 (0.92)
+ All	5395	470	4.562e-13 (1.52)	142	0.001764 (1.35)
			REF (*): 0.2145 (1.05)		REF (*): 0.9564 (0.84)

Supplemental Table S4. LNCipedia lncRNAs presenting with neuron specific H3K4me3, REST binding or DNAse 1 hypersensitivity at their transcription start site. For each mark or combination of marks, the number of genes harbouring a GWAS hit for CNS disorders, presenting with this mark is shown. Using Fisher's exact test it was determined whether these GWAS hits were significantly enriched in the resulting gene sets (odds ratios (OR) between brackets). In the second half of the table, the values following REF (*) represent enrichment using the H3K4me3 sets as reference.

	Total	*+ GWAS hit*	*Fisher p-val (& OR)*
**LNCipedia 2.1**	32108	255	= REF
+ REST binding	11348	91	0.489 (1.01)
+ H3K4me3 (*)	4188	53	0.001957 (1.60)
+ DNAseI hypersens.	17023	153	0.1227 (1.13)
+ H3K4me3 & REST	3109	38	0.01086 (1.55)
			REF (*): 0.605 (0.97)
+ H3K4me3 & DHS	3832	51	0.0008729 (1.68)
			REF (*): 0.4361 (1.05)
+ All	2980	38	0.00597 (1.61)
			REF (*): 0.526 (1.01)

Supplemental Table S5. GO enrichment of the protein-coding genes that either overlap with the 4188 lncRNAs with a neuron-specific H3K4me3 mark or that are transcribed within 5kb of these lncRNAs.

GO term	*ID*	*fold enrichment*	*p-value*
regulation of receptor activity	GO:0010469	3.43	8.08E-03
locomotory behavior	GO:0007626	2.6	1.66E-03
regulation of ion transmembrane transporter activity	GO:0032412	2.56	2.02E-02
regulation of transmembrane transporter activity	GO:0022898	2.5	3.20E-02
regulation of transporter activity	GO:0032409	2.47	2.04E-02
telencephalon development	GO:0021537	2.4	7.64E-03
single-organism behavior	GO:0044708	2.21	9.27E-05
forebrain development	GO:0030900	2.21	4.70E-04
positive regulation of nervous system development	GO:0051962	2.09	6.32E-04
positive regulation of neurogenesis	GO:0050769	2.02	2.24E-02
behavior	GO:0007610	2.01	1.25E-04
head development	GO:0060322	1.95	5.36E-06
brain development	GO:0007420	1.91	4.92E-05
positive regulation of cell development	GO:0010720	1.91	1.51E-02
regulation of neuron differentiation	GO:0045664	1.87	1.34E-02
regulation of nervous system development	GO:0051960	1.84	2.17E-04
synaptic transmission	GO:0007268	1.84	5.19E-03
regulation of neurogenesis	GO:0050767	1.81	5.00E-03
central nervous system development	GO:0007417	1.8	2.07E-05
neuron projection development	GO:0031175	1.74	4.33E-04
negative regulation of transcription from RNA polymerase II promoter	GO:0000122	1.73	4.45E-03
regulation of cell development	GO:0060284	1.71	4.22E-03
cell projection morphogenesis	GO:0048858	1.7	5.21E-04
cell part morphogenesis	GO:0032990	1.68	8.62E-04
neuron projection morphogenesis	GO:0048812	1.67	2.89E-02
regulation of cell cycle	GO:0051726	1.67	8.77E-04
neuron development	GO:0048666	1.66	6.15E-04
cell-cell signaling	GO:0007267	1.66	2.30E-03
neuron differentiation	GO:0030182	1.66	4.52E-05
cell morphogenesis	GO:0000902	1.66	1.17E-04
cell projection organization	GO:0030030	1.65	5.56E-05
generation of neurons	GO:0048699	1.65	2.11E-07
neurogenesis	GO:0022008	1.65	7.15E-08
nervous system development	GO:0007399	1.65	1.97E-12
response to growth factor	GO:0070848	1.64	4.34E-02
cell morphogenesis involved in differentiation	GO:0000904	1.64	1.52E-02
positive regulation of cell differentiation	GO:0045597	1.63	2.79E-02
cellular component morphogenesis	GO:0032989	1.63	1.45E-04
negative regulation of transcription, DNA-templated	GO:0045892	1.54	1.51E-02

negative regulation of nucleic acid-templated transcription	GO:1903507	1.53	1.54E-02
negative regulation of RNA biosynthetic process	GO:1902679	1.53	1.81E-02
negative regulation of RNA metabolic process	GO:0051253	1.52	1.48E-02
negative regulation of cellular macromolecule biosynthetic process	GO:2000113	1.52	1.01E-02
negative regulation of macromolecule biosynthetic process	GO:0010558	1.5	9.65E-03
negative regulation of gene expression	GO:0010629	1.49	7.95E-03
cell development	GO:0048468	1.49	2.79E-04
negative regulation of cellular biosynthetic process	GO:0031327	1.48	1.23E-02
negative regulation of biosynthetic process	GO:0009890	1.47	1.71E-02
negative regulation of nucleobase-containing compound metabolic process	GO:0045934	1.47	4.18E-02
regulation of localization	GO:0032879	1.47	9.85E-06
regulation of cell differentiation	GO:0045595	1.46	1.48E-02
regulation of transport	GO:0051049	1.45	3.44E-03
negative regulation of cellular metabolic process	GO:0031324	1.42	3.71E-04
phosphate-containing compound metabolic process	GO:0006796	1.42	1.61E-03
regulation of transcription from RNA polymerase II promoter	GO:0006357	1.42	1.86E-02
phosphorus metabolic process	GO:0006793	1.42	1.61E-03
negative regulation of macromolecule metabolic process	GO:0010605	1.4	1.53E-03
regulation of cell communication	GO:0010646	1.39	1.34E-05
regulation of developmental process	GO:0050793	1.39	7.25E-03
anatomical structure morphogenesis	GO:0009653	1.39	1.72E-03
negative regulation of metabolic process	GO:0009892	1.38	1.05E-03
system development	GO:0048731	1.38	2.82E-08
regulation of cellular component organization	GO:0051128	1.37	1.07E-02
regulation of signaling	GO:0023051	1.37	1.23E-04
negative regulation of cellular process	GO:0048523	1.37	6.91E-08
multicellular organismal development	GO:0007275	1.36	6.90E-09
regulation of molecular function	GO:0065009	1.35	1.62E-03
anatomical structure development	GO:0048856	1.35	7.95E-09
cellular protein modification process	GO:0006464	1.35	1.60E-03
protein modification process	GO:0036211	1.35	1.60E-03
regulation of catalytic activity	GO:0050790	1.34	4.57E-02
regulation of signal transduction	GO:0009966	1.34	1.26E-02
developmental process	GO:0032502	1.33	1.22E-09
single-organism developmental process	GO:0044767	1.33	3.34E-09
macromolecule modification	GO:0043412	1.32	4.63E-03
negative regulation of biological process	GO:0048519	1.32	2.53E-06
cellular developmental process	GO:0048869	1.31	4.09E-04
cell differentiation	GO:0030154	1.3	2.39E-03
positive regulation of cellular process	GO:0048522	1.3	8.02E-06
positive regulation of metabolic process	GO:0009893	1.29	2.99E-03
regulation of gene expression	GO:0010468	1.29	2.69E-04
positive regulation of biological process	GO:0048518	1.29	8.52E-07

regulation of macromolecule metabolic process	GO:0060255	1.28	8.43E-07
regulation of metabolic process	GO:0019222	1.27	5.25E-09
regulation of RNA metabolic process	GO:0051252	1.27	1.27E-02
regulation of nucleobase-containing compound metabolic process	GO:0019219	1.27	5.02E-03
regulation of response to stimulus	GO:0048583	1.27	2.06E-02
regulation of RNA biosynthetic process	GO:2001141	1.27	2.80E-02
regulation of nucleic acid-templated transcription	GO:1903506	1.27	3.16E-02
cellular component organization	GO:0016043	1.26	6.37E-05
regulation of transcription, DNA-templated	GO:0006355	1.26	4.59E-02
regulation of macromolecule biosynthetic process	GO:0010556	1.26	9.66E-03
regulation of cellular metabolic process	GO:0031323	1.26	2.58E-06
regulation of cellular macromolecule biosynthetic process	GO:2000112	1.26	2.05E-02
regulation of primary metabolic process	GO:0080090	1.25	1.82E-05
cellular component organization or biogenesis	GO:0071840	1.25	1.47E-04
regulation of nitrogen compound metabolic process	GO:0051171	1.25	1.17E-02
regulation of biosynthetic process	GO:0009889	1.24	2.86E-02
regulation of cellular biosynthetic process	GO:0031326	1.24	3.33E-02
localization	GO:0051179	1.23	8.95E-03
regulation of biological process	GO:0050789	1.21	1.33E-12
regulation of cellular process	GO:0050794	1.2	7.00E-11
cellular macromolecule metabolic process	GO:0044260	1.2	6.10E-04
biological regulation	GO:0065007	1.19	2.99E-12
cellular metabolic process	GO:0044237	1.19	8.31E-06
metabolic process	GO:0008152	1.18	1.33E-07
macromolecule metabolic process	GO:0043170	1.18	1.78E-03
primary metabolic process	GO:0044238	1.17	1.37E-04
organic substance metabolic process	GO:0071704	1.15	7.52E-04
cellular process	GO:0009987	1.14	7.52E-13
single-organism cellular process	GO:0044763	1.14	1.87E-05
single-organism process	GO:0044699	1.13	3.48E-07
biological_process	GO:0008150	1.1	1.75E-13
Unclassified	UNCLASSIFIED	0.6	0.00E+00
detection of stimulus	GO:0051606	0.34	1.39E-04
detection of stimulus involved in sensory perception	GO:0050906	< 0.2	7.99E-06
sensory perception of chemical stimulus	GO:0007606	< 0.2	6.28E-09
detection of chemical stimulus	GO:0009593	< 0.2	7.58E-10

Supplemental Table S6. Set of 53 lncRNAs with a neuron-specific H3K4me3 mark that overlap with a GWAS hit for CNS disorders. Chromosomal coordinates based on build hg19. In red: GWAS hit overlaps with both lncRNA transcripts and RefSeq protein-coding transcripts. It is indicated whether or not expression data was generated for these transcripts.

On array?	*Gene symbol*	*Chrom*	*Start*	*End*	*Strand*	*SNP identifier*
No	lnc-GPR39-4:2	chr2	134023767	134042334	+	rs7577925
No	lnc-DPYD-4:1	chr1	98453556	98515161	-	rs1625579
No	lnc-FES-2:1	chr15	91413148	91579841	+	rs2677744
No	lnc-USP25-2:1	chr21	17442842	17982094	+	rs2823819
No	lnc-USP25-2:3	chr21	17443553	17979456	+	rs2823819
No	lnc-CCDC102B-7:2	chr18	65183783	65566853	+	rs17077540, rs11660238, rs114535501, rs74789784, rs75687134, rs11661646
No	lnc-ACTR2-2:2	chr2	65663864	65919319	+	rs3845817
No	lnc-SMG6-1:1	chr17	2207756	2211342	-	rs4523957
No	lnc-GDAP1L1-1:2	chr20	42839632	42854667	+	rs6017291
No	lnc-USP25-2:2	chr21	17443434	17979450	+	rs2823819
No	lnc-PRKAG2-1:1	chr7	151532802	151576297	-	rs7795096
No	lnc-GDAP1L1-1:1	chr20	42839600	42854667	+	rs6017291
No	lnc-GDAP1L1-1:3	chr20	42839722	42854667	+	rs6017291
Yes	lnc-ELF2-2:1	chr4	139741111	139933800	-	rs77803164
Yes	lnc-RP11-210M15.2.1-1:3	chr15	80690251	80696189	-	rs2278702
Yes	lnc-AC073043.2.1-1:1	chr2	200527510	200715899	-	rs2949006
Yes	lnc-RP11-210M15.2.1-1:1	chr15	80665712	80696455	-	rs2278702
Yes	lnc-C22orf32-1:4	chr22	42670646	42673855	+	rs134882
No	lnc-FGGY-5:2	chr1	59250832	59366668	+	rs4601609
Yes	lnc-MYO10-1:1	chr5	17169365	17217508	-	rs2962370
No	lnc-FGGY-5:1	chr1	59250823	59365384	+	rs4601609
Yes	lnc-C22orf32-1:2	chr22	42665766	42671202	+	rs134882
No	lnc-A1BG-1:1	chr19	58768639	58790174	-	rs260461
Yes	lnc-MYO10-1:2	chr5	17130137	17217156	-	rs2962370
Yes	lnc-C22orf32-1:3	chr22	42665774	42671039	+	rs134882
Yes	lnc-RASGRF1-1:1	chr15	79529223	79576287	-	rs8025118
Yes	lnc-ANKMY1-2:1	chr2	241522117	241526116	-	rs1133353
Yes	lnc-EIF6-1:8	chr20	33813403	33865906	-	rs1555322
Yes	lnc-RASGRF1-1:7	chr15	79484049	79576167	-	rs8025118
No	lnc-PTPRB-1:3	chr12	70297813	70637138	-	rs789560
No	lnc-CCDC102B-7:1	chr18	65183783	65566856	+	rs17077540, rs11660238, rs114535501, rs74789784, rs75687134, rs11661646
Yes	lnc-RASGRF1-1:5	chr15	79529048	79576183	-	rs8025118
No	lnc-SCN1A-1:1	chr2	166929812	166984523	-	rs11890028
Yes	lnc-FANCF-3:1	chr11	22305276	22359791	-	rs11827962

Yes	lnc-MRE11A-1:2	chr11	94278496	94473435	-	rs2212361
Yes	lnc-EIF6-1:7	chr20	33812323	33865907	-	rs1555322
Yes	lnc-RASGRF1-1:6	chr15	79528908	79576173	-	rs8025118
No	lnc-RASGEF1B-1:4	chr4	82380633	82965397	-	rs1822818
Yes	lnc-RASGRF1-1:8	chr15	79529085	79576156	-	rs8025118
No	lnc-COPZ2-1:3	chr17	46122503	46125412	-	rs72823592
Yes	lnc-EIF6-1:6	chr20	33804634	33865925	-	rs1555322
Yes	lnc-RASGRF1-1:4	chr15	79505439	79576197	-	rs8025118
Yes	lnc-EIF6-1:5	chr20	33805628	33865928	-	rs1555322
Yes	lnc-RASGRF1-1:2	chr15	79529189	79576287	-	rs8025118
Yes	lnc-EIF6-1:2	chr20	33812547	33865934	-	rs1555322
No	lnc-CNBD1-4:3	chr8	89340073	89721769	+	rs11995572
Yes	lnc-CHAC2-4:1	chr2	51259739	52635055	+	rs1206397
Yes	lnc-NDUFAF2-2:1	chr5	60457897	60527867	+	rs171748
Yes	lnc-EIF6-1:11	chr20	33843840	33859859	-	rs1555322
Yes	lnc-MRE11A-1:1	chr11	94279357	94473521	-	rs2212361
No	lnc-SDIM1-2:1	chr6	165957021	166400091	-	rs1039002
Yes	lnc-RASGRF1-1:3	chr15	79484313	79576213	-	rs8025118
Yes	lnc-AC073043.2.1-1:2	chr2	200625267	200715896	-	rs2949006

Supplemental Table S7. Distance to the nearest protein-coding gene for the 53 selected lncRNAs (neuronspecific H3K4me3 & GWAS hit). Includes gene ID of the nearest gene and orientation of the lncRNA transcript relative to the nearest gene.

lncRNA	***distance (bp)***	***gene ID***	***orientation***
lnc-NDUFAF2-2:1	0	SMIM15	bidirectional
lnc-SDIM1-2:1	0	PDE10A	sense direction, separate promoter
lnc-RASGRF1-1:1	0	ANKRD34C	bidirectional
lnc-RASGRF1-1:4	0	ANKRD34C	bidirectional
lnc-RASGRF1-1:2	0	ANKRD34C	bidirectional
lnc-RASGRF1-1:7	0	ANKRD34C	bidirectional
lnc-RASGRF1-1:5	0	ANKRD34C	bidirectional
lnc-RASGRF1-1:3	0	ANKRD34C	bidirectional
lnc-RASGRF1-1:8	0	ANKRD34C	bidirectional
lnc-RASGRF1-1:6	0	ANKRD34C	bidirectional
lnc-SCN1A-1:1	0	SCN1A	sense direction, intronic
lnc-MYO10-1:1	0	BASP1	bidirectional
lnc-MYO10-1:2	0	BASP1	bidirectional
lnc-FES-2:1	0	VPS33B	overlapping antisense
lnc-MRE11A-1:1	0	FUT4	overlapping antisense
lnc-MRE11A-1:2	0	FUT4	overlapping antisense

lnc-EIF6-1:6	0	MMP24	overlapping antisense
lnc-EIF6-1:8	0	MMP24	overlapping antisense
lnc-EIF6-1:11	0	MMP24	overlapping antisense
lnc-EIF6-1:5	0	MMP24	overlapping antisense
lnc-EIF6-1:2	0	MMP24	overlapping antisense
lnc-EIF6-1:7	0	MMP24	overlapping antisense
lnc-A1BG-1:1	0	ZNF544	overlapping antisense
lnc-PTPRB-1:3	0	CNOT2	bidirectional
lnc-PRKAG2-1:1	0	PRKAG2	sense direction, same promoter
lnc-FANCF-3:1	0	SLC17A6	bidirectional
lnc-SMG6-1:1	0	SRR	overlapping antisense
lnc-CCDC102B-7:2	0	DSEL	bidirectional
lnc-CCDC102B-7:1	0	DSEL	bidirectional
lnc-RASGEF1B-1:4	0	RASGEF1B	sense direction, separate promoter
lnc-GPR39-4:2	0	NCKAP5	overlapping antisense
lnc-ANKMY1-2:1	16	CAPN10	bidirectional
lnc-GDAP1L1-1:1	53	OSER1	bidirectional
lnc-CHAC2-4:1	64	NRXN1	bidirectional
lnc-GDAP1L1-1:2	85	OSER1	bidirectional
lnc-GDAP1L1-1:3	175	OSER1	bidirectional
lnc-RP11-210M15.2.1-1:1	236	ARNT2	bidirectional
lnc-COPZ2-1:3	273	NFE2L1	bidirectional
lnc-CNBD1-4:3	355	MMP16	bidirectional
lnc-RP11-210M15.2.1-1:3	502	ARNT2	bidirectional
lnc-FGGY-5:1	1037	JUN	bidirectional
lnc-FGGY-5:2	1046	JUN	bidirectional
lnc-ELF2-2:1	3112	CCRN4L	bidirectional
lnc-ACTR2-2:2	4207	SPRED2	bidirectional
lnc-C22orf32-1:2	54418	TCF20	free-standing promoter region
lnc-C22orf32-1:3	54426	TCF20	free-standing promoter region
lnc-C22orf32-1:4	59298	TCF20	free-standing promoter region
lnc-AC073043.2.1-1:1	60079	C2orf69	free-standing promoter region
lnc-AC073043.2.1-1:2	60082	C2orf69	free-standing promoter region
lnc-DPYD-4:1	66940	DPYD	free-standing promoter region
lnc-USP25-2:1	190451	USP25	free-standing promoter region
lnc-USP25-2:2	191043	USP25	free-standing promoter region
lnc-USP25-2:3	191162	USP25	free-standing promoter region

Supplemental Table S8. The total number of protein-coding genes and lncRNA transcripts that were covered on the expression array, subsets of coding genes and lncRNAs exhibiting expression in at least one brain region (normalized log2 expression value > 8) & subsets of coding genes and lncRNAs upregulated in neuronal vs nonneuronal tissues. For each subset, the number of ID genes or genes harbouring a GWAS hit for CNS disorders, present within these subsets is shown. Using Fisher's exact test it was determined whether these ID genes and GWAS hits were significantly enriched in the resulting gene sets (odds ratios between brackets).

	Total	***ID genes***	***Fisher p-val (& OR)***	***+GWAS hit***	***Fisher p-val (& OR)***
Coding genes on array	21863	1133	= REF	356	= REF
1 brain region > 8	11472	839	6.861e-15 (1.44)	243	0.0009 (1.31)
Upregulated (FDR 0.01)	1290	124	2.999e-10 (1.95)	65	7.497e-14 (3.21)
lncRNAs on array	22980	*NA*	*NA*	161	= REF
1 brain region > 8	1350	*NA*	*NA*	5	0.9566 (0.53)
Upregulated (FDR 0.01)	731	*NA*	*NA*	4	0.7524 (0.78)

Supplemental Table S9. Enrichment of ID genes among protein-coding genes highly correlated to the 30 selected lncRNAs. P-values were determined using Fisher's exact test. Odds ratios are indicated in column *OR*.

Gene Symbol	*p-value*	*OR*
lnc-AC073043.2.1-1:1	0.0277	1.27
lnc-AC073043.2.1-1:2	1.0000	0.00
lnc-ANKMY1-2:1	0.5844	1.00
lnc-C22orf32-1:2	0.5929	1.02
lnc-C22orf32-1:3	0.9282	0.55
lnc-C22orf32-1:4	1.03E-04	1.31
lnc-CHAC2-4:1	0.8171	0.64
lnc-EIF6-1:11	0.0043	1.92
lnc-EIF6-1:2	0.2369	1.16
lnc-EIF6-1:5	0.9901	0.47
lnc-EIF6-1:6	0.8392	0.80
lnc-EIF6-1:7	0.0140	1.31
lnc-EIF6-1:8	0.5927	0.96
lnc-ELF2-2:1	0.9858	0.23
lnc-FANCF-3:1	0.1541	1.33
lnc-MRE11A-1:1	0.1231	1.18
lnc-MRE11A-1:2	0.5322	1.14
lnc-MYO10-1:1	3.46E-05	1.32
lnc-MYO10-1:2	6.85E-04	1.36
lnc-NDUFAF2-2:1	0.2277	1.37
lnc-RASGRF1-1:1	4.11E-04	1.67
lnc-RASGRF1-1:2	3.08E-04	1.42
lnc-RASGRF1-1:3	0.1609	1.20
lnc-RASGRF1-1:4	0.9604	0.39
lnc-RASGRF1-1:5	1.25E-04	1.71
lnc-RASGRF1-1:6	0.0176	1.32
lnc-RASGRF1-1:7	0.1689	1.12
lnc-RASGRF1-1:8	2.20E-05	1.64
lnc-RP11-210M15.2.1-1:1	0.0485	1.26
lnc-RP11-210M15.2.1-1:3	0.2904	1.10

Supplemental Table S10. Top results gene set enrichment analysis. Tabular results of the gene set enrichment analysis for the 30 selected lncRNAs that were covered on the array. For each lncRNA, the top 10 positively and negatively enriched genes sets among highly correlated protein-coding genes are displayed, along with the size of the gene set (size), the enrichment score (ES), the normalized ES (NES) and the FDR q-value (FDR q-val).

lnc-ANKMY1-2-1

NAME	SIZE	ES	NES	FDR q-val
SYNAPTIC TRANSMISSION	172	0,4492896	2,2026694	0,009342879
POTASSIUM CHANNELS	98	0,4845476	2,1919734	0,00467144
TRANSMISSION OF NERVE IMPULSE	187	0,4358767	2,166768	0,005284713
NEURONAL SYSTEM	276	0,41253668	2,162646	0,004142908
VOLTAGE GATED POTASSIUM CHANNELS	43	0,56314206	2,1514227	0,00405011
POTASSIUM ION TRANSPORT	58	0,5062999	2,0794892	0,010003676
NEUROLOGICAL SYSTEM PROCESS	376	0,37792385	2,0424585	0,013833577
GABA SYNTHESIS RELEASE REUPTAKE AND DEGRADATION	17	0,6693574	2,0339305	0,013274495
TRANSMISSION ACROSS CHEMICAL SYNAPSES	184	0,40591577	2,030079	0,012194995
MONOVALENT INORGANIC CATION TRANSPORT	94	0,45359987	2,0143182	0,01278005
METABOLISM OF MRNA	210	-0,65845126	-3,5067534	0
INFLUENZA LIFE CYCLE	134	-0,7708557	-3,8659077	0
STRUCTURAL CONSTITUENT OF RIBOSOME	80	-0,861354	-3,8739047	0
TRANSLATION	145	-0,77748555	-3,905625	0
SRP DEPENDENT COTRANSLATIONAL PROTEIN TARGETING TO MEMBRANE	109	-0,8239559	-3,9250977	0
NONSENSE MEDIATED DECAY ENHANCED BY THE EXON JUNCTION COMPLEX	106	-0,82948095	-3,9402876	0
INFLUENZA VIRAL RNA TRANSCRIPTION AND REPLICATION	100	-0,84542394	-4,009516	0
3 UTR MEDIATED TRANSLATIONAL REGULATION	104	-0,8608798	-4,0592346	0
PEPTIDE CHAIN ELONGATION	85	-0,8989841	-4,1474376	0
RIBOSOME	87	-0,9138409	-4,2693453	0

lnc-C22orf32-1-2

NAME	SIZE	ES	NES	FDR q-val
VALINE LEUCINE AND ISOLEUCINE DEGRADATION	44	0,6606592	2,8007379	0
OLFACTORY SIGNALING PATHWAY	308	0,42818463	2,5269618	0
PROPANOATE METABOLISM	32	0,63421685	2,4949539	0
PENTOSE AND GLUCURONATE INTERCONVERSIONS	18	0,7269266	2,4593709	0
FATTY ACID METABOLISM	42	0,58199286	2,428918	2,17E-04
TCA CYCLE AND RESPIRATORY ELECTRON TRANSPORT	128	0,45774886	2,3905284	3,63E-04
BRANCHED CHAIN AMINO ACID CATABOLISM	17	0,7348639	2,3476574	7,47E-04
MITOCHONDRION	340	0,38499776	2,311383	9,22E-04
RESPIRATORY ELECTRON TRANSPORT ATP SYNTHESIS BY CHEMIOSMOTIC COUPLING AND HEAT PRODUCTION BY UNCOUPLING PROTEINS	92	0,47224486	2,3027787	9,38E-04
OXIDOREDUCTASE ACTIVITY	285	0,3921884	2,2905972	9,53E-04
CELL DIVISION	17	-0,6630015	-2,021353	0,009862444
NOTCH SIGNALING PATHWAY	47	-0,53272074	-2,0298333	0,009514707
SEMAPHORIN INTERACTIONS	64	-0,5084609	-2,0473073	0,007730591
PACKAGING OF TELOMERE ENDS	47	-0,5329114	-2,049381	0,007502937
AXON GUIDANCE	240	-0,4264629	-2,0705554	0,005868312
MELANOGENESIS	101	-0,47217825	-2,0754948	0,006146894
TRANSMISSION OF NERVE IMPULSE	187	-0,43780082	-2,0871692	0,005546508
NEURONAL SYSTEM	276	-0,42475882	-2,095293	0,005680386
RNA POL I PROMOTER OPENING	57	-0,5315667	-2,1032422	0,005365432
NERVOUS SYSTEM DEVELOPMENT	380	-0,41600665	-2,1075642	0,006828381
SYNAPTIC TRANSMISSION	172	-0,45915562	-2,1674378	0,004888581
AXON GUIDANCE	129	-0,5051517	-2,2970212	0

lnc-C22orf32-1-4

NAME	SIZE	ES	NES	FDR q-val
IMMUNOREGULATORY INTERACTIONS BETWEEN A LYMPHOID AND A NON LYMPHOID CELL	62	0,7329552	3,5076463	0
COMPLEMENT AND COAGULATION CASCADES	68	0,66209376	3,3282514	0
CYTOKINE CYTOKINE RECEPTOR INTERACTION	257	0,52831197	3,2472026	0
INTERFERON GAMMA SIGNALING	59	0,67056215	3,2356024	0
GRAFT VERSUS HOST DISEASE	40	0,73846674	3,22291	0
DEFENSE RESPONSE	266	0,5107085	3,21443	0
INTERFERON ALPHA BETA SIGNALING	59	0,65077454	3,1692665	0

IMMUNE RESPONSE	228	0,5244566	3,1527162	0
COMPLEMENT CASCADE	29	0,78760445	3,140831	0
ALLOGRAFT REJECTION	38	0,73689514	3,111945	0
ACTIVATION OF NMDA RECEPTOR UPON GLUTAMATE BINDING AND POSTSYNAPTIC EVENTS	37	-0,6449733	-2,504999	0
GLUTAMATE RECEPTOR ACTIVITY	20	-0,7679544	-2,55117	0
TRANSMISSION OF NERVE IMPULSE	187	-0,49958596	-2,5940652	0
MICROTUBULE ASSOCIATED COMPLEX	47	-0,63085616	-2,6209087	0
CENTRAL NERVOUS SYSTEM DEVELOPMENT	120	-0,54159236	-2,644754	0
SYNAPTIC TRANSMISSION	172	-0,52489626	-2,6922991	0
NEUROTRANSMITTER RECEPTOR BINDING AND DOWNSTREAM TRANSMISSION IN THE POSTSYNAPTIC CELL	135	-0,5506569	-2,7387538	0
NERVOUS SYSTEM DEVELOPMENT	380	-0,49280974	-2,783831	0
NEURONAL SYSTEM	276	-0,5236253	-2,8597486	0
TRANSMISSION ACROSS CHEMICAL SYNAPSES	184	-0,5545223	-2,8871763	0

lnc-CHAC2-4-1

NAME	SIZE	ES	NES	FDR q-val
NEURONAL SYSTEM	276	0,41390553	2,1872215	0,016085295
SYNAPTOGENESIS	17	0,7009822	2,146351	0,014784247
NERVOUS SYSTEM DEVELOPMENT	380	0,39221933	2,1410894	0,010159952
MITOTIC G2 G2 M PHASES	77	0,48405254	2,1404386	0,007844547
SYNAPTIC TRANSMISSION	172	0,4320134	2,135324	0,006275638
GLYCOSAMINOGLYCAN BIOSYNTHESIS HEPARAN SULFATE	26	0,60777277	2,13316	0,005532276
LOSS OF NLP FROM MITOTIC CENTROSOMES	55	0,5191105	2,1097896	0,006781406
RECRUITMENT OF MITOTIC CENTROSOME PROTEINS AND COMPLEXES	62	0,49604604	2,103856	0,00626963
PARKINSONS DISEASE	124	0,4328449	2,0595748	0,010252594
TRANSMISSION OF NERVE IMPULSE	187	0,40711954	2,0483224	0,011381611
NOD LIKE RECEPTOR SIGNALING PATHWAY	62	-0,58467156	-2,7566247	0
CHEMOKINE RECEPTORS BIND CHEMOKINES	53	-0,6112682	-2,810421	0
INTERFERON GAMMA SIGNALING	59	-0,62613505	-2,838279	0
HEMATOPOIETIC CELL LINEAGE	86	-0,5585061	-2,851793	0
DEFENSE RESPONSE	266	-0,47396094	-2,8630092	0
INTESTINAL IMMUNE NETWORK FOR IGA PRODUCTION	48	-0,64245707	-2,869996	0
AUTOIMMUNE THYROID DISEASE	49	-0,6600013	-2,936282	0
CYTOKINE CYTOKINE RECEPTOR INTERACTION	257	-0,50324106	-3,0360174	0
GRAFT VERSUS HOST DISEASE	40	-0,7042687	-3,0437925	0
ALLOGRAFT REJECTION	38	-0,73115945	-3,1256907	0

lnc-EIF6-1-2

NAME	SIZE	ES	NES	FDR q-val
RIBOSOME	87	0,7561491	3,9033473	0
PEPTIDE CHAIN ELONGATION	85	0,7499919	3,7743704	0
3 UTR MEDIATED TRANSLATIONAL REGULATION	104	0,6984991	3,7239025	0
STRUCTURAL CONSTITUENT OF RIBOSOME	80	0,7322333	3,6622694	0
INFLUENZA VIRAL RNA TRANSCRIPTION AND REPLICATION	100	0,67664856	3,5363176	0
SYSTEMIC LUPUS ERYTHEMATOSUS	128	0,6367532	3,5262158	0
NONSENSE MEDIATED DECAY ENHANCED BY THE EXON JUNCTION COMPLEX	106	0,66706914	3,5174553	0
TRANSLATION	145	0,60610205	3,4313452	0
GRAFT VERSUS HOST DISEASE	40	0,79738957	3,4069989	0
SRP DEPENDENT COTRANSLATIONAL PROTEIN TARGETING TO MEMBRANE	109	0,6440853	3,4017904	0
GABA SYNTHESIS RELEASE REUPTAKE AND DEGRADATION	17	-0,73636454	-2,3651638	6,76E-05
VOLTAGE GATED POTASSIUM CHANNELS	43	-0,5857396	-2,3772209	7,51E-05
POTASSIUM CHANNELS	98	-0,48629788	-2,3792913	8,45E-05
TRAFFICKING OF AMPA RECEPTORS	27	-0,65996015	-2,397866	0
GLUTAMATE RECEPTOR ACTIVITY	20	-0,760347	-2,5349715	0
TRANSMISSION OF NERVE IMPULSE	187	-0,48590535	-2,6010478	0
NEUROTRANSMITTER RECEPTOR BINDING AND DOWNSTREAM TRANSMISSION IN THE POSTSYNAPTIC CELL	135	-0,5096652	-2,6283383	0
SYNAPTIC TRANSMISSION	172	-0,50362706	-2,6716712	0
TRANSMISSION ACROSS CHEMICAL SYNAPSES	184	-0,51081866	-2,7425616	0
NEURONAL SYSTEM	276	-0,49733952	-2,8175073	0

lnc-EIF6-1-5

NAME	SIZE	ES	NES	FDR q-val
PEPTIDE CHAIN ELONGATION	85	0,6705369	3,487663	0
RIBOSOME	87	0,6730843	3,4553552	0
STRUCTURAL CONSTITUENT OF RIBOSOME	80	0,65288377	3,3135347	0
3 UTR MEDIATED TRANSLATIONAL REGULATION	104	0,60743934	3,2845488	0
INFLUENZA VIRAL RNA TRANSCRIPTION AND REPLICATION	100	0,6119382	3,251018	0
INTERFERON GAMMA SIGNALING	59	0,6877889	3,244344	0

GRAFT VERSUS HOST DISEASE	40	0,75264364	3,2382848	0
IMMUNOREGULATORY INTERACTIONS BETWEEN A LYMPHOID AND A NON LYMPHOID CELL	62	0,6650176	3,157807	0
ALLOGRAFT REJECTION	38	0,74584275	3,1458383	0
NONSENSE MEDIATED DECAY ENHANCED BY THE EXON JUNCTION COMPLEX	106	0,5765504	3,124287	0
POTASSIUM CHANNELS	98	-0,47843355	-2,254103	1,62E-04
ALANINE ASPARTATE AND GLUTAMATE METABOLISM	32	-0,61264676	-2,2602527	1,80E-04
CHOLESTEROL BIOSYNTHESIS	22	-0,6934862	-2,321338	1,01E-04
TRAFFICKING OF AMPA RECEPTORS	27	-0,65174544	-2,3239477	1,15E-04
NEUROTRANSMITTER RECEPTOR BINDING AND DOWNSTREAM TRANSMISSION IN THE POSTSYNAPTIC CELL	135	-0,47519687	-2,3258126	1,35E-04
TRANSMISSION OF NERVE IMPULSE	187	-0,47062042	-2,3879206	1,62E-04
TRANSMISSION ACROSS CHEMICAL SYNAPSES	184	-0,47164765	-2,3956897	2,02E-04
GLUTAMATE RECEPTOR ACTIVITY	20	-0,732676	-2,4001791	2,69E-04
SYNAPTIC TRANSMISSION	172	-0,49228594	-2,4573057	0
NEURONAL SYSTEM	276	-0,4692058	-2,496607	0

lnc-EIF6-1-6

NAME	SIZE	ES	NES	FDR q-val
RIBOSOME	87	0,67082834	3,45663	0
GRAFT VERSUS HOST DISEASE	40	0,77767926	3,3682132	0
PEPTIDE CHAIN ELONGATION	85	0,6489437	3,3259835	0
STRUCTURAL CONSTITUENT OF RIBOSOME	80	0,63682926	3,2494705	0
INTERFERON GAMMA SIGNALING	59	0,6831884	3,2444947	0
SRP DEPENDENT COTRANSLATIONAL PROTEIN TARGETING TO MEMBRANE	109	0,59497076	3,1972566	0
ALLOGRAFT REJECTION	38	0,75557363	3,195102	0
AUTOIMMUNE THYROID DISEASE	49	0,7124111	3,194067	0
INFLUENZA VIRAL RNA TRANSCRIPTION AND REPLICATION	100	0,59979796	3,1672084	0
DEFENSE RESPONSE	266	0,5114984	3,1548862	0
GLUTAMATE SIGNALING PATHWAY	17	-0,7070809	-2,2578552	1,45E-04
CENTRAL NERVOUS SYSTEM DEVELOPMENT	120	-0,4763757	-2,3267589	8,22E-05
VOLTAGE GATED POTASSIUM CHANNELS	43	-0,5754099	-2,3376143	9,25E-05
NERVOUS SYSTEM DEVELOPMENT	380	-0,42202437	-2,396868	0
NEUROTRANSMITTER RECEPTOR BINDING AND DOWNSTREAM TRANSMISSION IN THE POSTSYNAPTIC CELL	135	-0,48687452	-2,4264321	0
TRANSMISSION OF NERVE IMPULSE	187	-0,4861336	-2,5307875	0
TRANSMISSION ACROSS CHEMICAL SYNAPSES	184	-0,4836222	-2,5461872	0
SYNAPTIC TRANSMISSION	172	-0,5041648	-2,608696	0
NEURONAL SYSTEM	276	-0,47460014	-2,6294425	0
GLUTAMATE RECEPTOR ACTIVITY	20	-0,77562046	-2,6456475	0

lnc-EIF6-1-7

NAME	SIZE	ES	NES	FDR q-val
NEURONAL SYSTEM	276	0,5325068	2,9946516	0
TRANSMISSION ACROSS CHEMICAL SYNAPSES	184	0,55519164	2,984313	0
NEUROTRANSMITTER RECEPTOR BINDING AND DOWNSTREAM TRANSMISSION IN THE POSTSYNAPTIC CELL	135	0,54835016	2,7760565	0
SYNAPTIC TRANSMISSION	172	0,5221243	2,7742922	0
TRANSMISSION OF NERVE IMPULSE	187	0,49880457	2,6894011	0
NERVOUS SYSTEM DEVELOPMENT	380	0,45281532	2,6318405	0
GLUTAMATE RECEPTOR ACTIVITY	20	0,7823414	2,6309576	0
GABA SYNTHESIS RELEASE REUPTAKE AND DEGRADATION	17	0,7793669	2,5026765	7,85E-05
GLUTAMATE SIGNALING PATHWAY	17	0,76051503	2,4883523	6,98E-05
POTASSIUM CHANNELS	98	0,5026157	2,4426672	6,28E-05
DRUG METABOLISM CYTOCHROME P450	60	-0,575418	-2,739794	0
ALLOGRAFT REJECTION	38	-0,6476613	-2,7534277	0
TRANSLATION	145	-0,49873218	-2,8100846	0
3 UTR MEDIATED TRANSLATIONAL REGULATION	104	-0,5313889	-2,8205783	0
STRUCTURAL CONSTITUENT OF RIBOSOME	80	-0,5813272	-2,91949	0
PEPTIDE CHAIN ELONGATION	85	-0,5804402	-2,9546666	0
SRP DEPENDENT COTRANSLATIONAL PROTEIN TARGETING TO MEMBRANE	109	-0,5657558	-2,9959276	0
COMPLEMENT AND COAGULATION CASCADES	68	-0,62608886	-3,0232508	0
RIBOSOME	87	-0,5987458	-3,0673656	0
IMMUNOREGULATORY INTERACTIONS BETWEEN A LYMPHOID AND A NON LYMPHOID CELL	62	-0,6548619	-3,1543715	0

lnc-EIF6-1-8

NAME	SIZE	ES	NES	FDR q-val
NEURONAL SYSTEM	276	0,47255456	2,595698	0
POTASSIUM CHANNELS	98	0,52413005	2,4670517	0
SYNAPTIC TRANSMISSION	172	0,46871793	2,466003	0

VOLTAGE GATED POTASSIUM CHANNELS	43	0,6153997	2,446353	0
VOLTAGE GATED POTASSIUM CHANNEL ACTIVITY	36	0,6126445	2,4234934	0
TRANSMISSION ACROSS CHEMICAL SYNAPSES	184	0,46122453	2,4162872	0
TRANSMISSION OF NERVE IMPULSE	187	0,44834268	2,3368216	1,14E-04
NERVOUS SYSTEM DEVELOPMENT	380	0,4000112	2,2964237	1,99E-04
POTASSIUM CHANNEL ACTIVITY	50	0,5509626	2,2870524	1,77E-04
OXIDATIVE PHOSPHORYLATION	127	0,45773175	2,281822	1,60E-04
PEPTIDE CHAIN ELONGATION	85	-0,49979392	-2,411953	1,25E-04
BIOLOGICAL OXIDATIONS	124	-0,47659037	-2,432318	0
AMI PATHWAY	20	-0,7276858	-2,4523113	0
STEROID HORMONE BIOSYNTHESIS	44	-0,5905645	-2,4537325	0
TRANSLATION	145	-0,45794705	-2,4635801	0
COMPLEMENT CASCADE	29	-0,66316974	-2,51603	0
ASPARAGINE N LINKED GLYCOSYLATION	79	-0,52597183	-2,5192206	0
PHASE II CONJUGATION	57	-0,56715995	-2,5371485	0
RIBOSOME	87	-0,5139492	-2,5376158	0
SRP DEPENDENT COTRANSLATIONAL PROTEIN TARGETING TO MEMBRANE	109	-0,5027184	-2,5706449	0

lnc-FANCF-3-1

NAME	SIZE	ES	NES	FDR q-val
RIBONUCLEOPROTEIN COMPLEX	143	0,46319282	2,207569	0,008523552
RNA PROCESSING	170	0,4384772	2,1431103	0,009775279
PROTEIN FOLDING	51	0,5342803	2,1395175	0,006795866
GABA SYNTHESIS RELEASE REUPTAKE AND DEGRADATION	17	0,69953376	2,1350956	0,005305114
ANTIGEN PROCESSING UBIQUITINATION PROTEASOME DEGRADATION	199	0,4245395	2,1171787	0,005088627
NEURONAL SYSTEM	276	0,41008064	2,110393	0,004804246
NEUROTRANSMITTER RECEPTOR BINDING AND DOWNSTREAM TRANSMISSION IN THE POSTSYNAPTIC CELL	135	0,44412217	2,091993	0,005925484
MICROTUBULE ASSOCIATED COMPLEX	47	0,52427596	2,0879438	0,005610781
ASSOCIATION OF TRIC CCT WITH TARGET PROTEINS DURING BIOSYNTHESIS	26	0,6096941	2,0873125	0,004987361
SPLICEOSOME	51	0,51196665	2,0658329	0,006351464
HEMATOPOIETIC CELL LINEAGE	86	-0,5555286	-2,9042444	0
DEFENSE RESPONSE	266	-0,46633035	-2,9161003	0
IMMUNE RESPONSE	228	-0,4767162	-3,001252	0
ASTHMA	30	-0,7495004	-3,111482	0
INTESTINAL IMMUNE NETWORK FOR IGA PRODUCTION	48	-0,6653374	-3,1127195	0
CYTOKINE CYTOKINE RECEPTOR INTERACTION	257	-0,4928994	-3,1607933	0
GRAFT VERSUS HOST DISEASE	40	-0,72614217	-3,2044332	0
AUTOIMMUNE THYROID DISEASE	49	-0,7127383	-3,2695997	0
IMMUNOREGULATORY INTERACTIONS BETWEEN A LYMPHOID AND A NON LYMPHOID CELL	62	-0,66996145	-3,2718787	0
ALLOGRAFT REJECTION	38	-0,75752836	-3,2980666	0

lnc-MRE11A-1-1

NAME	SIZE	ES	NES	FDR q-val
NEURONAL SYSTEM	276	0,5599306	3,278764	0
TRANSMISSION ACROSS CHEMICAL SYNAPSES	184	0,5799643	3,1718495	0
NEUROTRANSMITTER RECEPTOR BINDING AND DOWNSTREAM TRANSMISSION IN THE POSTSYNAPTIC CELL	135	0,5624963	2,9945455	0
SYNAPTIC TRANSMISSION	172	0,5397499	2,9886765	0
TRANSMISSION OF NERVE IMPULSE	187	0,52580947	2,9135885	0
NEUROTRANSMITTER RELEASE CYCLE	34	0,7059252	2,8293784	0
GABA SYNTHESIS RELEASE REUPTAKE AND DEGRADATION	17	0,85144025	2,791937	0
VOLTAGE GATED POTASSIUM CHANNELS	43	0,6497839	2,7427402	0
POTASSIUM CHANNELS	98	0,549658	2,7128181	0
GLUTAMATE RECEPTOR ACTIVITY	20	0,7703693	2,6114326	0
FORMATION OF THE TERNARY COMPLEX AND SUBSEQUENTLY THE 43S COMPLEX	47	-0,691243	-3,0292542	0
INFLUENZA LIFE CYCLE	134	-0,5845372	-3,1549654	0
INFLUENZA VIRAL RNA TRANSCRIPTION AND REPLICATION	100	-0,6349278	-3,2166224	0
STRUCTURAL CONSTITUENT OF RIBOSOME	80	-0,6804455	-3,2950304	0
NONSENSE MEDIATED DECAY ENHANCED BY THE EXON JUNCTION COMPLEX	106	-0,6453501	-3,354439	0
3 UTR MEDIATED TRANSLATIONAL REGULATION	104	-0,6691233	-3,3868825	0
TRANSLATION	145	-0,6266278	-3,4117632	0
SRP DEPENDENT COTRANSLATIONAL PROTEIN TARGETING TO MEMBRANE	109	-0,6820578	-3,5083778	0
PEPTIDE CHAIN ELONGATION	85	-0,71298563	-3,5095487	0
RIBOSOME	87	-0,7316209	-3,6994789	0

lnc-MYO10-1-1

NAME	SIZE	ES	NES	FDR q-val
NEURONAL SYSTEM	276	0,5108852	2,7683668	0
NERVOUS SYSTEM DEVELOPMENT	380	0,48780444	2,7263505	0

TRANSMISSION ACROSS CHEMICAL SYNAPSES	184	0,52641445	2,7197227	0
SYNAPTIC TRANSMISSION	172	0,5291583	2,7154818	0
TRANSMISSION OF NERVE IMPULSE	187	0,5047259	2,6255517	0
NEUROTRANSMITTER RECEPTOR BINDING AND DOWNSTREAM TRANSMISSION IN THE POSTSYNAPTIC CELL	135	0,52557397	2,615042	0
CENTRAL NERVOUS SYSTEM DEVELOPMENT	120	0,5312716	2,6081436	0
MICROTUBULE ASSOCIATED COMPLEX	47	0,6341772	2,5966833	0
GLUTAMATE RECEPTOR ACTIVITY	20	0,78218687	2,573988	0
NOS1 PATHWAY	21	0,7418983	2,493137	7,64E-05
IMMUNE RESPONSE	228	-0,5303839	-3,2537968	0
HEMATOPOIETIC CELL LINEAGE	86	-0,625318	-3,3055272	0
REACTOME INTERFERON GAMMA SIGNALING	59	-0,6962692	-3,3068306	0
COMPLEMENT AND COAGULATION CASCADES	68	-0,66089076	-3,3069174	0
DEFENSE RESPONSE	266	-0,5273184	-3,3150814	0
AUTOIMMUNE THYROID DISEASE	49	-0,7240291	-3,3503437	0
ALLOGRAFT REJECTION	38	-0,7771535	-3,3684008	0
GRAFT VERSUS HOST DISEASE	40	-0,7912206	-3,3969917	0
CYTOKINE CYTOKINE RECEPTOR INTERACTION	257	-0,56107956	-3,4939928	0
IMMUNOREGULATORY INTERACTIONS BETWEEN A LYMPHOID AND A NON LYMPHOID CELL	62	-0,7680719	-3,8136096	0

lnc-MYO10-1-2

NAME	SIZE	ES	NES	FDR q-val
SYNAPTIC TRANSMISSION	172	0,54631215	2,6946228	0
TRANSMISSION OF NERVE IMPULSE	187	0,5215542	2,5772212	0
NEURONAL SYSTEM	276	0,49536383	2,5466194	0
NEUROTRANSMITTER RECEPTOR BINDING AND DOWNSTREAM TRANSMISSION IN THE POSTSYNAPTIC CELL	135	0,5278837	2,531246	0
TRANSMISSION ACROSS CHEMICAL SYNAPSES	184	0,5032906	2,4896314	0
NERVOUS SYSTEM DEVELOPMENT	380	0,46653384	2,4834394	0
GLUTAMATE RECEPTOR ACTIVITY	20	0,73146516	2,3262806	0
NOS1 PATHWAY	21	0,69105035	2,287771	0
TRAFFICKING OF AMPA RECEPTORS	27	0,6544913	2,2873108	0
GABA SYNTHESIS RELEASE REUPTAKE AND DEGRADATION	17	0,7426384	2,2871048	0
BIOCARTA COMP PATHWAY	18	-0,863451	-3,07538	0
INTERFERON GAMMA SIGNALING	59	-0,6497199	-3,1824539	0
CYTOKINE CYTOKINE RECEPTOR INTERACTION	257	-0,5104294	-3,1829123	0
IMMUNE RESPONSE	228	-0,5105859	-3,200105	0
ALLOGRAFT REJECTION	38	-0,74416745	-3,230301	0
DEFENSE RESPONSE	266	-0,5237793	-3,2543633	0
AUTOIMMUNE THYROID DISEASE	49	-0,69577426	-3,3231287	0
GRAFT VERSUS HOST DISEASE	40	-0,7309264	-3,345213	0
COMPLEMENT AND COAGULATION CASCADES	68	-0,64185274	-3,354882	0
IMMUNOREGULATORY INTERACTIONS BETWEEN A LYMPHOID AND A NON LYMPHOID CELL	62	-0,71117914	-3,496974	0

lnc-RASGRF1-1-1

NAME	SIZE	ES	NES	FDR q-val
NEURONAL SYSTEM	276	0,5095883	2,8691492	0
TRANSMISSION ACROSS CHEMICAL SYNAPSES	184	0,52892214	2,8193378	0
NEUROTRANSMITTER RECEPTOR BINDING AND DOWNSTREAM TRANSMISSION IN THE POSTSYNAPTIC CELL	135	0,52106607	2,689993	0
GLUTAMATE RECEPTOR ACTIVITY	20	0,79629976	2,6489882	0
NERVOUS SYSTEM DEVELOPMENT	380	0,44969776	2,6179297	0
SYNAPTIC TRANSMISSION	172	0,4802008	2,563889	0
ACTIVATION OF NMDA RECEPTOR UPON GLUTAMATE BINDING AND POSTSYNAPTIC EVENTS	37	0,6497302	2,5437822	0
GLUTAMATE SIGNALING PATHWAY	17	0,768705	2,452052	0
TRANSMISSION OF NERVE IMPULSE	187	0,4582889	2,4447563	0
POST NMDA RECEPTOR ACTIVATION EVENTS	33	0,6387999	2,4359734	0
ANTIGEN PRESENTATION FOLDING ASSEMBLY AND PEPTIDE LOADING OF CLASS I MHC	20	-0,7160905	-2,6043792	0
ENDOPLASMIC RETICULUM PART	96	-0,49678168	-2,6260192	0
DRUG METABOLISM OTHER ENZYMES	40	-0,5979301	-2,6441278	0
GRAFT VERSUS HOST DISEASE	40	-0,61624956	-2,6679847	0
ALLOGRAFT REJECTION	38	-0,6395975	-2,7420337	0
DRUG METABOLISM CYTOCHROME P450	60	-0,5732239	-2,7765138	0
STEROID HORMONE BIOSYNTHESIS	44	-0,6292503	-2,8326738	0
COMPLEMENT CASCADE	29	-0,73935866	-2,9579976	0
IMMUNOREGULATORY INTERACTIONS BETWEEN A LYMPHOID AND A NON LYMPHOID CELL	62	-0,6188209	-3,0005894	0

COMPLEMENT AND COAGULATION CASCADES	68	-0,64007527	-3,1817524	0

lnc-RASGRF1-1-2

NAME	SIZE	ES	NES	FDR q-val
NEURONAL SYSTEM	276	0,5150513	2,5752485	0
TRANSMISSION ACROSS CHEMICAL SYNAPSES	184	0,5208638	2,5206332	0
NEUROTRANSMITTER RECEPTOR BINDING AND DOWNSTREAM TRANSMISSION IN THE POSTSYNAPTIC CELL	135	0,540023	2,512656	0
SYNAPTIC TRANSMISSION	172	0,5148335	2,4581447	0
TRANSMISSION OF NERVE IMPULSE	187	0,49662387	2,3979893	0
GLUTAMATE RECEPTOR ACTIVITY	20	0,7324359	2,381719	0
POTASSIUM CHANNELS	98	0,52699715	2,3539839	0
NERVOUS SYSTEM DEVELOPMENT	380	0,4596973	2,3354268	0
VOLTAGE GATED POTASSIUM CHANNELS	43	0,59961784	2,3179212	0
TRAFFICKING OF AMPA RECEPTORS	27	0,6666043	2,3099594	0
COMPLEMENT CASCADE	29	-0,7392081	-3,0627737	0
IMMUNE RESPONSE	228	-0,5014276	-3,1184354	0
CYTOKINE CYTOKINE RECEPTOR INTERACTION	257	-0,49804777	-3,1685035	0
INTESTINAL IMMUNE NETWORK FOR IGA PRODUCTION	48	-0,6691475	-3,2039764	0
COMPLEMENT AND COAGULATION CASCADES	68	-0,6349524	-3,2088306	0
INTERFERON GAMMA SIGNALING	59	-0,6541066	-3,2488196	0
GRAFT VERSUS HOST DISEASE	40	-0,7411012	-3,3097954	0
IMMUNOREGULATORY INTERACTIONS BETWEEN A LYMPHOID AND A NON LYMPHOID CELL	62	-0,6886013	-3,3920655	0
ALLOGRAFT REJECTION	38	-0,772475	-3,3987052	0
AUTOIMMUNE THYROID DISEASE	49	-0,7144517	-3,4029238	0

lnc-RASGRF1-1-3

NAME	SIZE	ES	NES	FDR q-val
OXIDATIVE PHOSPHORYLATION	127	0,5523169	2,791969	0
RESPIRATORY ELECTRON TRANSPORT ATP SYNTHESIS BY CHEMIOSMOTIC COUPLING AND HEAT PRODUCTION BY UNCOUPLING PROTEINS	92	0,5713636	2,7089381	0
NEURONAL SYSTEM	276	0,47978264	2,6871743	0
RESPIRATORY ELECTRON TRANSPORT	74	0,5888121	2,6696894	0
PARKINSONS DISEASE	124	0,52657175	2,6665132	0
TCA CYCLE AND RESPIRATORY ELECTRON TRANSPORT	128	0,5164519	2,6160595	0
TRANSMISSION ACROSS CHEMICAL SYNAPSES	184	0,49291807	2,6056545	0
SYNAPTIC TRANSMISSION	172	0,46768284	2,4915519	0
TRANSMISSION OF NERVE IMPULSE	187	0,45733008	2,4359808	0
GABA SYNTHESIS RELEASE REUPTAKE AND DEGRADATION	17	0,7664698	2,4075835	0
SYSTEMIC LUPUS ERYTHEMATOSUS	128	-0,46555507	-2,576911	0
CHEMOKINE RECEPTORS BIND CHEMOKINES	53	-0,5650499	-2,603788	0
SRP DEPENDENT COTRANSLATIONAL PROTEIN TARGETING TO MEMBRANE	109	-0,49311808	-2,6488562	0
COMPLEMENT CASCADE	29	-0,68509555	-2,6876872	0
GRAFT VERSUS HOST DISEASE	40	-0,63184935	-2,7302632	0
IMMUNOREGULATORY INTERACTIONS BETWEEN A LYMPHOID AND A NON LYMPHOID CELL	62	-0,5756311	-2,7478297	0
ALLOGRAFT REJECTION	38	-0,654568	-2,7482953	0
COMPLEMENT AND COAGULATION CASCADES	68	-0,56408995	-2,781741	0
PEPTIDE CHAIN ELONGATION	85	-0,5727199	-2,904941	0
RIBOSOME	87	-0,5919026	-3,0238607	0

lnc-RASGRF1-1-4

NAME	SIZE	ES	NES	FDR q-val
NEURONAL SYSTEM	276	0,4926603	2,8340044	0
TRANSMISSION ACROSS CHEMICAL SYNAPSES	184	0,49018005	2,6894443	0
GABA SYNTHESIS RELEASE REUPTAKE AND DEGRADATION	17	0,80236477	2,6407573	0
NEUROTRANSMITTER RELEASE CYCLE	34	0,64541185	2,5374608	0
TRAFFICKING OF AMPA RECEPTORS	27	0,6992311	2,5220644	0
TRANSMISSION OF NERVE IMPULSE	187	0,45522615	2,5019724	0
NOS1 PATHWAY	21	0,71547997	2,46303	0
SYNAPTIC TRANSMISSION	172	0,45862764	2,4577708	0
VOLTAGE GATED POTASSIUM CHANNELS	43	0,5938165	2,4389112	0
NEUROTRANSMITTER RECEPTOR BINDING AND DOWNSTREAM TRANSMISSION IN THE POSTSYNAPTIC CELL	135	0,4518666	2,3567796	2,99E-04
FORMATION OF THE TERNARY COMPLEX AND SUBSEQUENTLY THE 43S COMPLEX	47	-0,63310605	-2,6315522	0
INFLUENZA LIFE CYCLE	134	-0,5200267	-2,6322355	0
INFLUENZA VIRAL RNA TRANSCRIPTION AND REPLICATION	100	-0,5755219	-2,7755113	0
TRANSLATION	145	-0,54644746	-2,8346577	0
SRP DEPENDENT COTRANSLATIONAL PROTEIN TARGETING TO MEMBRANE	109	-0,5965674	-2,868404	0
NONSENSE MEDIATED DECAY ENHANCED BY THE EXON JUNCTION COMPLEX	106	-0,5843568	-2,875583	0

STRUCTURAL CONSTITUENT OF RIBOSOME	80	-0,624833	-2,8776844	0
3 UTR MEDIATED TRANSLATIONAL REGULATION	104	-0,59877706	-2,9249547	0
PEPTIDE CHAIN ELONGATION	85	-0,65538675	-3,0567884	0
RIBOSOME	87	-0,67672527	-3,2443612	0

lnc-RASGRF1-1-5

NAME	SIZE	ES	NES	FDR q-val
NEURONAL SYSTEM	276	0,5280783	2,776153	0
TRANSMISSION ACROSS CHEMICAL SYNAPSES	184	0,53047985	2,6859453	0
NEUROTRANSMITTER RECEPTOR BINDING AND DOWNSTREAM TRANSMISSION IN THE POSTSYNAPTIC CELL	135	0,5325789	2,5664766	0
SYNAPTIC TRANSMISSION	172	0,51135755	2,557506	0
NERVOUS SYSTEM DEVELOPMENT	380	0,46310708	2,5251746	0
GLUTAMATE RECEPTOR ACTIVITY	20	0,76177484	2,4901214	0
TRANSMISSION OF NERVE IMPULSE	187	0,4904069	2,4792995	0
GLUTAMATE SIGNALING PATHWAY	17	0,7652626	2,4449508	0
GABA SYNTHESIS RELEASE REUPTAKE AND DEGRADATION	17	0,7833629	2,4435952	0
POTASSIUM CHANNELS	98	0,5282204	2,4244823	0
DEFENSE RESPONSE	266	-0,4564999	-2,9035823	0
CHEMOKINE RECEPTORS BIND CHEMOKINES	53	-0,6075739	-2,9253848	0
IMMUNE RESPONSE	228	-0,4717226	-2,951231	0
AUTOIMMUNE THYROID DISEASE	49	-0,63360447	-2,9795623	0
DRUG METABOLISM CYTOCHROME P450	60	-0,61110324	-3,009392	0
CYTOKINE CYTOKINE RECEPTOR INTERACTION	257	-0,4671559	-3,013795	0
GRAFT VERSUS HOST DISEASE	40	-0,68782234	-3,0239723	0
ALLOGRAFT REJECTION	38	-0,696607	-3,0452628	0
IMMUNOREGULATORY INTERACTIONS BETWEEN A LYMPHOID AND A NON LYMPHOID CELL	62	-0,65241826	-3,2182226	0
COMPLEMENT AND COAGULATION CASCADES	68	-0,6387078	-3,235392	0

lnc-RASGRF1-1-6

NAME	SIZE	ES	NES	FDR q-val
TRANSMISSION ACROSS CHEMICAL SYNAPSES	184	0,5825224	3,168566	0
NEURONAL SYSTEM	276	0,5422234	3,092173	0
NEUROTRANSMITTER RECEPTOR BINDING AND DOWNSTREAM TRANSMISSION IN THE POSTSYNAPTIC CELL	135	0,55298847	2,8513508	0
GABA SYNTHESIS RELEASE REUPTAKE AND DEGRADATION	17	0,82517743	2,6759195	0
SYNAPTIC TRANSMISSION	172	0,48488033	2,6213176	0
TRANSMISSION OF NERVE IMPULSE	187	0,46951872	2,5629668	0
TRAFFICKING OF AMPA RECEPTORS	27	0,6744128	2,5303166	0
POTASSIUM CHANNELS	98	0,50901306	2,5027328	0
NEUROTRANSMITTER RELEASE CYCLE	34	0,6453958	2,4735582	0
NOS1 PATHWAY	21	0,7073753	2,4552023	0
INFLUENZA VIRAL RNA TRANSCRIPTION AND REPLICATION	100	-0,6290029	-3,2893555	0
SYSTEMIC LUPUS ERYTHEMATOSUS	128	-0,5953049	-3,3201392	0
NONSENSE MEDIATED DECAY ENHANCED BY THE EXON JUNCTION COMPLEX	106	-0,64059734	-3,4176452	0
IMMUNOREGULATORY INTERACTIONS BETWEEN A LYMPHOID AND A NON LYMPHOID CELL	62	-0,70648205	-3,4177415	0
STRUCTURAL CONSTITUENT OF RIBOSOME	80	-0,6927568	-3,4950724	0
3 UTR MEDIATED TRANSLATIONAL REGULATION	104	-0,66440463	-3,4963417	0
TRANSLATION	145	-0,6363334	-3,5955067	0
PEPTIDE CHAIN ELONGATION	85	-0,72165877	-3,689618	0
SRP DEPENDENT COTRANSLATIONAL PROTEIN TARGETING TO MEMBRANE	109	-0,69219124	-3,7239754	0
RIBOSOME	87	-0,7300353	-3,794266	0

lnc-RASGRF1-1-7

NAME	SIZE	ES	NES	FDR q-val
NEURONAL SYSTEM	276	0,55467325	2,9057152	0
TRANSMISSION ACROSS CHEMICAL SYNAPSES	184	0,5689692	2,8682	0
NEUROTRANSMITTER RECEPTOR BINDING AND DOWNSTREAM TRANSMISSION IN THE POSTSYNAPTIC CELL	135	0,55421644	2,7108214	0
SYNAPTIC TRANSMISSION	172	0,54177314	2,7067745	0
TRANSMISSION OF NERVE IMPULSE	187	0,52888286	2,672622	0
GABA SYNTHESIS RELEASE REUPTAKE AND DEGRADATION	17	0,8155969	2,5428946	0
POTASSIUM CHANNELS	98	0,54934233	2,5361316	0
POTASSIUM ION TRANSPORT	58	0,59180665	2,4876883	0
VOLTAGE GATED POTASSIUM CHANNELS	43	0,62589306	2,4744718	0
AMINE RECEPTOR ACTIVITY	34	0,65470856	2,4671028	0
DRUG METABOLISM CYTOCHROME P450	60	-0,575418	-2,739794	0
ALLOGRAFT REJECTION	38	-0,6476613	-2,7534277	0
TRANSLATION	145	-0,49873218	-2,8100846	0

3 UTR MEDIATED TRANSLATIONAL REGULATION	104	-0,5313889	-2,8205783	0
STRUCTURAL CONSTITUENT OF RIBOSOME	80	-0,5813272	-2,91949	0
PEPTIDE CHAIN ELONGATION	85	-0,5804402	-2,9546666	0
SRP DEPENDENT COTRANSLATIONAL PROTEIN TARGETING TO MEMBRANE	109	-0,5657558	-2,9959276	0
COMPLEMENT AND COAGULATION CASCADES	68	-0,62608886	-3,0232508	0
RIBOSOME	87	-0,5987458	-3,0673656	0
IMMUNOREGULATORY INTERACTIONS BETWEEN A LYMPHOID AND A NON LYMPHOID CELL	62	-0,6548619	-3,1543715	0

lnc-RASGRF1-1-8

NAME	SIZE	ES	NES	FDR q-val
NERVOUS SYSTEM DEVELOPMENT	380	0,49730083	2,6395676	0
NEURONAL SYSTEM	276	0,503877	2,6073053	0
CENTRAL NERVOUS SYSTEM DEVELOPMENT	120	0,5426788	2,5517056	0
TRANSMISSION ACROSS CHEMICAL SYNAPSES	184	0,5098273	2,5274472	0
SYNAPTIC TRANSMISSION	172	0,51764774	2,5189335	0
TRANSMISSION OF NERVE IMPULSE	187	0,49942327	2,4854226	0
NEUROTRANSMITTER RECEPTOR BINDING AND DOWNSTREAM TRANSMISSION IN THE POSTSYNAPTIC CELL	135	0,51675856	2,4622724	0
GABA SYNTHESIS RELEASE REUPTAKE AND DEGRADATION	17	0,78901225	2,4550467	0
GLUTAMATE RECEPTOR ACTIVITY	20	0,75912017	2,4288495	0
RAS ACTIVATION UOPN CA2 INFUX THROUGH NMDA RECEPTOR	17	0,74125516	2,3580825	8,57E-05
COMPLEMENT AND COAGULATION CASCADES	68	-0,6167165	-3,0524247	0
HEMATOPOIETIC CELL LINEAGE	86	-0,58039284	-3,054816	0
IMMUNE RESPONSE	228	-0,49759072	-3,056011	0
DEFENSE RESPONSE	266	-0,49383706	-3,1041288	0
INTESTINAL IMMUNE NETWORK FOR IGA PRODUCTION	48	-0,66470754	-3,109922	0
CYTOKINE CYTOKINE RECEPTOR INTERACTION	257	-0,49506795	-3,1314957	0
ALLOGRAFT REJECTION	38	-0,74973756	-3,2494938	0
IMMUNOREGULATORY INTERACTIONS BETWEEN A LYMPHOID AND A NON LYMPHOID CELL	62	-0,6714284	-3,2860618	0
GRAFT VERSUS HOST DISEASE	40	-0,73685324	-3,2953813	0
AUTOIMMUNE THYROID DISEASE	49	-0,70901954	-3,3488352	0

lnc-RP11-210M15.2.1-1:1

NAME	SIZE	ES	NES	FDR q-val
SYNAPTIC TRANSMISSION	172	0,49690756	2,4929347	0
TRANSMISSION ACROSS CHEMICAL SYNAPSES	184	0,48821443	2,4884653	0
NEURONAL SYSTEM	276	0,46760586	2,4872355	0
NEUROTRANSMITTER RECEPTOR BINDING AND DOWNSTREAM TRANSMISSION IN THE POSTSYNAPTIC CELL	135	0,50766677	2,4798405	0
TRANSMISSION OF NERVE IMPULSE	187	0,47939348	2,4299593	0
NERVOUS SYSTEM DEVELOPMENT	380	0,44616374	2,419105	0
GABA RECEPTOR ACTIVATION	52	0,56860185	2,3522737	0
GABA SYNTHESIS RELEASE REUPTAKE AND DEGRADATION	17	0,73394364	2,3121202	2,69E-04
CENTRAL NERVOUS SYSTEM DEVELOPMENT	120	0,47261986	2,2662876	4,01E-04
GLUTAMATE RECEPTOR ACTIVITY	20	0,6929328	2,253059	5,06E-04
COMPLEMENT CASCADE	29	-0,7658274	-3,1677203	0
CYTOKINE CYTOKINE RECEPTOR INTERACTION	257	-0,5020189	-3,1705048	0
DEFENSE RESPONSE	266	-0,5100667	-3,206695	0
STRUCTURAL CONSTITUENT OF RIBOSOME	80	-0,6208614	-3,2505422	0
3 UTR MEDIATED TRANSLATIONAL REGULATION	104	-0,6049427	-3,2548769	0
TRANSLATION	145	-0,55716175	-3,2715225	0
PEPTIDE CHAIN ELONGATION	85	-0,6612059	-3,4298434	0
SRP DEPENDENT COTRANSLATIONAL PROTEIN TARGETING TO MEMBRANE	109	-0,6241357	-3,4451823	0
RIBOSOME	87	-0,66689104	-3,4881854	0
IMMUNOREGULATORY INTERACTIONS BETWEEN A LYMPHOID AND A NON LYMPHOID CELL	62	-0,7482783	-3,6365867	0

lnc-RP11-210M15.2.1-1:3

NAME	SIZE	ES	NES	FDR q-val
NEURONAL SYSTEM	276	0,53040344	2,9240122	0
TRANSMISSION ACROSS CHEMICAL SYNAPSES	184	0,54912496	2,8868659	0
NEUROTRANSMITTER RECEPTOR BINDING AND DOWNSTREAM TRANSMISSION IN THE POSTSYNAPTIC CELL	135	0,5498614	2,7624788	0
SYNAPTIC TRANSMISSION	172	0,49870792	2,5994108	0
TRANSMISSION OF NERVE IMPULSE	187	0,49400225	2,5764365	0
NERVOUS SYSTEM DEVELOPMENT	380	0,45374078	2,561003	0
GABA RECEPTOR ACTIVATION	52	0,6070946	2,5206556	0
GABA SYNTHESIS RELEASE REUPTAKE AND DEGRADATION	17	0,75957394	2,5028698	0
POTASSIUM CHANNELS	98	0,5178671	2,4711702	0

TRAFFICKING OF AMPA RECEPTORS	27	0,6752573	2,4353335	0
ACTIVATION OF THE MRNA UPON BINDING OF THE CAP BINDING COMPLEX AND EIFS AND SUBSEQUENT BINDING TO 43S	55	-0,7079256	-3,4128215	0
FORMATION OF THE TERNARY COMPLEX AND SUBSEQUENTLY THE 43S COMPLEX	47	-0,75256723	-3,5278435	0
NONSENSE MEDIATED DECAY ENHANCED BY THE EXON JUNCTION COMPLEX	106	-0,65437883	-3,642373	0
INFLUENZA VIRAL RNA TRANSCRIPTION AND REPLICATION	100	-0,6632747	-3,6523795	0
STRUCTURAL CONSTITUENT OF RIBOSOME	80	-0,70521957	-3,6925917	0
TRANSLATION	145	-0,6883876	-3,9447055	0
3 UTR MEDIATED TRANSLATIONAL REGULATION	104	-0,7208144	-3,9524035	0
PEPTIDE CHAIN ELONGATION	85	-0,7557258	-3,974038	0
RIBOSOME	87	-0,7556359	-4,0405803	0
SRP DEPENDENT COTRANSLATIONAL PROTEIN TARGETING TO MEMBRANE	109	-0,73904026	-4,078234	0

lnc-AC073043.2.1-1:1

NAME	SIZE	ES	NES	FDR q-val
RESPIRATORY ELECTRON TRANSPORT	74	0,6232613	2,784768	0
TCA CYCLE AND RESPIRATORY ELECTRON TRANSPORT	128	0,56573856	2,7465146	0
NEURONAL SYSTEM	276	0,51473707	2,7427146	0
RESPIRATORY ELECTRON TRANSPORT ATP SYNTHESIS BY CHEMIOSMOTIC COUPLING AND HEAT PRODUCTION BY UNCOUPLING PROTEINS	92	0,5882098	2,733511	0
TRANSMISSION ACROSS CHEMICAL SYNAPSES	184	0,5324716	2,7187345	0
OXIDATIVE PHOSPHORYLATION	127	0,5499766	2,6850266	0
TRANSMISSION OF NERVE IMPULSE	187	0,5146008	2,6646593	0
SYNAPTIC TRANSMISSION	172	0,52399445	2,652044	0
NEUROTRANSMITTER RECEPTOR BINDING AND DOWNSTREAM TRANSMISSION IN THE POSTSYNAPTIC CELL	135	0,52514946	2,559072	0
MITOCHONDRIAL PART	141	0,51111627	2,52754	0
ALLOGRAFT REJECTION	38	-0,7502839	-3,4783254	0
TRANSLATION	145	-0,57841206	-3,5004728	0
STRUCTURAL CONSTITUENT OF RIBOSOME	80	-0,6742036	-3,5967963	0
SYSTEMIC LUPUS ERYTHEMATOSUS	128	-0,59650874	-3,6393387	0
INFLUENZA VIRAL RNA TRANSCRIPTION AND REPLICATION	100	-0,66178	-3,656961	0
SRP DEPENDENT COTRANSLATIONAL PROTEIN TARGETING TO MEMBRANE	109	-0,6413804	-3,6574192	0
NONSENSE MEDIATED DECAY ENHANCED BY THE EXON JUNCTION COMPLEX	106	-0,66429484	-3,7502458	0
3 UTR MEDIATED TRANSLATIONAL REGULATION	104	-0,68626463	-3,9804149	0
RIBOSOME	87	-0,74280244	-4,0627794	0
PEPTIDE CHAIN ELONGATION	85	-0,75524724	-4,080634	0

lnc-EIF6-1:11

NAME	SIZE	ES	NES	FDR q-val
ALLOGRAFT REJECTION	38	0,6327306	2,6245372	0
GENERIC TRANSCRIPTION PATHWAY	340	0,40881148	2,4539282	4,20E-04
INTESTINAL IMMUNE NETWORK FOR IGA PRODUCTION	48	0,5268729	2,3118262	0,00309427
GRAFT VERSUS HOST DISEASE	40	0,5519435	2,2695625	0,003795879
AUTOIMMUNE THYROID DISEASE	49	0,51055354	2,210769	0,007224588
TH1TH2 PATHWAY	19	0,61271906	2,113179	0,019164687
PRIMARY IMMUNODEFICIENCY	35	0,5030808	2,0490139	0,029891184
CTLA4 PATHWAY	19	0,5843495	2,0283751	0,031605784
TASTE RECEPTOR ACTIVITY	15	0,62740976	2,0232694	0,029867284
INTERLEUKIN RECEPTOR ACTIVITY	20	0,5750369	1,991342	0,035371393
MITOCHONDRIAL ENVELOPE	96	-0,5809441	-2,530953	0
MITOCHONDRIAL MEMBRANE PART	52	-0,65229875	-2,5519626	0
OXIDATIVE PHOSPHORYLATION	127	-0,58016753	-2,62372	0
PARKINSONS DISEASE	124	-0,5772941	-2,6414669	0
TCA CYCLE AND RESPIRATORY ELECTRON TRANSPORT	128	-0,59410954	-2,6810308	0
MITOCHONDRIAL MATRIX	46	-0,70482385	-2,6825721	0
MITOCHONDRIAL LUMEN	46	-0,70482385	-2,7138076	0
RESPIRATORY ELECTRON TRANSPORT	74	-0,6611902	-2,7716105	0
RESPIRATORY ELECTRON TRANSPORT ATP SYNTHESIS BY CHEMIOSMOTIC COUPLING AND HEAT PRODUCTION BY UNCOUPLING PROTEINS	92	-0,6479525	-2,7998962	0
MITOCHONDRIAL PART	141	-0,60367405	-2,804802	0

lnc-MRE11A-1:2

NAME	SIZE	ES	NES	FDR q-val
RETINOL METABOLISM	53	0,53062415	2,1576102	0,034606356
MRNA PROCESSING	155	-0,44568676	-2,0079448	0,035654243
MRNA SPLICING	108	-0,47529444	-2,0366406	0,03257659
GLYCOSAMINOGLYCAN BIOSYNTHESIS CHONDROITIN SULFATE	22	-0,67038286	-2,040397	0,04505841
PROCESSING OF CAPPED INTRON CONTAINING PRE MRNA	137	-0,4662675	-2,0811987	0,048211273

lnc-AC073043.2.1-1:2

NAME	SIZE	ES	NES	FDR q-val
TCA CYCLE AND RESPIRATORY ELECTRON TRANSPORT	128	0,625049	2,8047073	0
RESPIRATORY ELECTRON TRANSPORT ATP SYNTHESIS BY CHEMIOSMOTIC COUPLING AND HEAT PRODUCTION BY UNCOUPLING PROTEINS	92	0,60847133	2,6081276	0
PYRUVATE METABOLISM AND CITRIC ACID TCA CYCLE	40	0,70293707	2,5064566	0
OXIDATIVE PHOSPHORYLATION	127	0,5565894	2,4810305	0
RESPIRATORY ELECTRON TRANSPORT	74	0,60463786	2,4477117	0
MITOCHONDRIAL PART	141	0,5398109	2,4349961	0
MITOCHONDRION	340	0,48492625	2,4318006	0
PARKINSONS DISEASE	124	0,5316232	2,3583496	0
MITOCHONDRIAL ENVELOPE	96	0,5596055	2,3492477	0
CITRIC ACID CYCLE TCA CYCLE	19	0,78021616	2,3214364	0
MEIOSIS	108	-0,5177374	-2,3935907	7,75E-05
MEIOTIC SYNAPSIS	70	-0,5649756	-2,4627306	8,61E-05
RNA POL I TRANSCRIPTION	80	-0,56929225	-2,5211954	0
CHROMOSOME MAINTENANCE	117	-0,55323786	-2,5892012	0
TELOMERE MAINTENANCE	74	-0,6041145	-2,6237702	0
AMYLOIDS	76	-0,6071018	-2,6336126	0
MEIOTIC RECOMBINATION	80	-0,59823626	-2,6490374	0
PACKAGING OF TELOMERE ENDS	47	-0,6987213	-2,7592237	0
RNA POL I PROMOTER OPENING	57	-0,7087754	-2,9058263	0
DEPOSITION OF NEW CENPA CONTAINING NUCLEOSOMES AT THE CENTROMERE	61	-0,728423	-3,0327184	0

lnc-NDUFAF2-2:1

NAME	SIZE	ES	NES	FDR q-val
INTERFERON GAMMA SIGNALING	59	0,4608759	2,0463896	0,14284958
HEMATOPOIETIN INTERFERON CLASSD200 DOMAIN CYTOKINE RECEPTOR BINDING	26	0,54847735	1,9969741	0,1184564
OLFACTORY SIGNALING PATHWAY	308	0,34604433	1,9840016	0,08964363
AUTOIMMUNE THYROID DISEASE	49	0,45570788	1,9160954	0,12033231
OLFACTORY TRANSDUCTION	375	0,31612185	1,8319495	0,20015746
ANTIGEN PROCESSING AND PRESENTATION	80	0,38229662	1,8135067	0,19225027
CELL RECOGNITION	19	0,5446645	1,7972555	0,18737084
PROTEIN KINASE INHIBITOR ACTIVITY	24	0,48134083	1,7298776	0,2800796
AMYLOIDS	76	0,37610057	1,7186875	0,26936686
KINASE INHIBITOR ACTIVITY	25	0,47018656	1,6948724	0,2899446
MITOCHONDRIAL MEMBRANE PART	52	-0,7074642	-2,5768769	0
MITOCHONDRIAL ENVELOPE	96	-0,63598734	-2,5783067	0
PARKINSONS DISEASE	124	-0,6337025	-2,5923946	0
MITOCHONDRIAL INNER MEMBRANE	66	-0,690031	-2,6050467	0
MITOCHONDRIAL MEMBRANE	85	-0,6681851	-2,6336365	0
MITOCHONDRIAL PART	141	-0,6371959	-2,6570425	0
RESPIRATORY ELECTRON TRANSPORT	74	-0,7024981	-2,6827335	0
MITOCHONDRION	340	-0,591047	-2,6992908	0
RESPIRATORY ELECTRON TRANSPORT ATP SYNTHESIS BY CHEMIOSMOTIC COUPLING AND HEAT PRODUCTION BY UNCOUPLING PROTEINS	92	-0,69385064	-2,785895	0
TCA CYCLE AND RESPIRATORY ELECTRON TRANSPORT	128	-0,6985176	-2,8910687	0

lnc-ELF2-2:1

NAME	SIZE	ES	NES	FDR q-val
RIBOSOME	87	0,68392104	3,2032273	0
SRP DEPENDENT COTRANSLATIONAL PROTEIN TARGETING TO MEMBRANE	109	0,65894055	3,1799173	0
TRANSLATION	145	0,6142308	3,1395187	0
INFLUENZA VIRAL RNA TRANSCRIPTION AND REPLICATION	100	0,6512656	3,0985	0
PEPTIDE CHAIN ELONGATION	85	0,66726196	3,0860004	0
3 UTR MEDIATED TRANSLATIONAL REGULATION	104	0,63524985	3,0448453	0
INFLUENZA LIFE CYCLE	134	0,5916454	2,9727683	0
STRUCTURAL CONSTITUENT OF RIBOSOME	80	0,6359608	2,9459584	0
NONSENSE MEDIATED DECAY ENHANCED BY THE EXON JUNCTION COMPLEX	106	0,6069873	2,9103959	0
METABOLISM OF MRNA	210	0,50759816	2,7296011	0
VOLTAGE GATED CALCIUM CHANNEL ACTIVITY	18	-0,6320633	-1,8806955	0,07078257
VOLTAGE GATED CALCIUM CHANNEL COMPLEX	15	-0,66779095	-1,8835179	0,076212175
POTASSIUM ION TRANSPORT	58	-0,48233262	-1,8966202	0,07231462
CATION CHANNEL ACTIVITY	119	-0,4299729	-1,8968878	0,08264528
PHOSPHATIDYLINOSITOL SIGNALING SYSTEM	76	-0,47158888	-1,9117377	0,07987229
CALCIUM CHANNEL ACTIVITY	33	-0,5713332	-1,9725524	0,03835697
CALCIUM SIGNALING PATHWAY	177	-0,42531225	-1,9982458	0,031896036
AMINE RECEPTOR ACTIVITY	34	-0,59413224	-2,032536	0,024936378
CHANNEL REGULATOR ACTIVITY	24	-0,6485927	-2,0743277	0,021557484
AUXILIARY TRANSPORT PROTEIN ACTIVITY	26	-0,6361249	-2,1395562	0,011466905

lnc-C22orf32-1:3

NAME	SIZE	ES	NES	FDR q-val
AMYLOIDS	76	0,6228138	2,7081153	0
RNA POL I PROMOTER OPENING	57	0,6234721	2,5769315	0
TELOMERE MAINTENANCE	74	0,5770284	2,525705	0
PACKAGING OF TELOMERE ENDS	47	0,6526617	2,4919922	0
SYSTEMIC LUPUS ERYTHEMATOSUS	128	0,5021381	2,3989668	0
GRAFT VERSUS HOST DISEASE	40	0,5471143	2,1411178	0,007702211
RESPIRATORY ELECTRON TRANSPORT	74	0,47871444	2,1291344	0,008389808
STRUCTURAL MOLECULE ACTIVITY	240	0,40201703	2,1141708	0,009101516
LEISHMANIA INFECTION	70	0,49132612	2,1087863	0,008713789
RESPIRATORY ELECTRON TRANSPORT ATP SYNTHESIS BY CHEMIOSMOTIC COUPLING AND HEAT PRODUCTION BY UNCOUPLING PROTEINS	92	0,46164063	2,1076038	0,007985538
MICROTUBULE MOTOR ACTIVITY	16	-0,61156887	-1,8001049	0,16060363
PEROXISOMAL LIPID METABOLISM	20	-0,56669426	-1,8126085	0,15657842
DNA HELICASE ACTIVITY	25	-0,5542787	-1,8504013	0,119339205
CHROMATIN ASSEMBLY	16	-0,64177835	-1,876639	0,10116896
RNA DEGRADATION	57	-0,47089094	-1,8776776	0,11640002
HELICASE ACTIVITY	51	-0,48968336	-1,8875506	0,124746226
CHROMATIN ASSEMBLY OR DISASSEMBLY	26	-0,58605075	-1,9229771	0,10704415
CENTROSOME	57	-0,4769275	-1,9420035	0,11611539
BIOSYNTHESIS OF THE N GLYCAN PRECURSOR DOLICHOL LIPID LINKED OLIGOSACCHARIDE LLO AND TRANSFER TO A NASCENT PROTEIN	28	-0,5862363	-1,9814057	0,09863212
GENERIC TRANSCRIPTION PATHWAY	340	-0,5222872	-2,707673	0
